# Expression of clock genes tracks daily and tidal time in brains of intertidal crustaceans *Eurydice pulchra* and *Parhyale hawaiensis*

**DOI:** 10.1016/j.cub.2025.04.047

**Published:** 2025-05-08

**Authors:** Andrew Oliphant, Chee Y. Sia, Charalambos P. Kyriacou, David C. Wilcockson, Michael H. Hastings

**Affiliations:** 1 Neurobiology Division, MRC Laboratory of Molecular Biology, Francis Crick Avenue, Cambridge CB2 0QH, UK; 2 Department of Genetics, Genomics and Cancer Sciences, University of Leicester, University Road, Leicester LE1 7RH, UK; 3 Department of Life Sciences, Aberystwyth University, Penglais, Aberystwyth SY23 3DA, UK

## Abstract

Intertidal organisms, such as the crustaceans *Eurydice pulchra* and *Parhyale hawaiensis*, express daily and tidal rhythms of physiology and behavior to adapt to their temporally complex environments. Although the molecular-genetic basis of the circadian clocks driving daily rhythms in terrestrial animals is well understood, the nature of the circatidal clocks driving tidal rhythms remains a mystery. Using *in situ* hybridization, we identified discrete clusters of ∼60 putative “clock” cells co-expressing canonical circadian clock genes across the protocerebrum of *E. pulchra* and *P. hawaiensis* brains. In field-collected, tidally rhythmic *E. pulchra* sampled under a light:dark (LD) cycle, the expression of *period* (*per*) and *cryptochrome 2* (*cry2*) exhibited daily rhythms in particular cell groups, whereas *timeless* (*tim*) showed 12-h rhythms in others. In tidally rhythmic laboratory-reared *P. hawaiensis*, previously entrained to 12.4-h cycles of agitation under LD and sampled under continuous darkness, several cell groups (e.g., medioposterior cells) exhibited circadian expression of *per* and *cry2*. In contrast, dorsal-lateral cells in the protocerebrum exhibited robust ∼12-h, i.e., circatidal, rhythms of *per* and *cry2*, phased to the prior tidal agitation but not the prior LD. In *P. hawaiensis* exhibiting daily behavior under LD without tidal agitation, robust daily rhythms of *per* and *cry2* expression were evident in medioposterior and other cells, whereas expression in dorsal-lateral cells was not rhythmic, underlining their essentially tidal periodicity. These results implicate canonical circadian molecules in circatidal timekeeping and reveal conserved brain networks as potential neural substrates for the generation of daily and tidal rhythms appropriate to intertidal habitats.

## Graphical abstract

**Graphical abstract:**
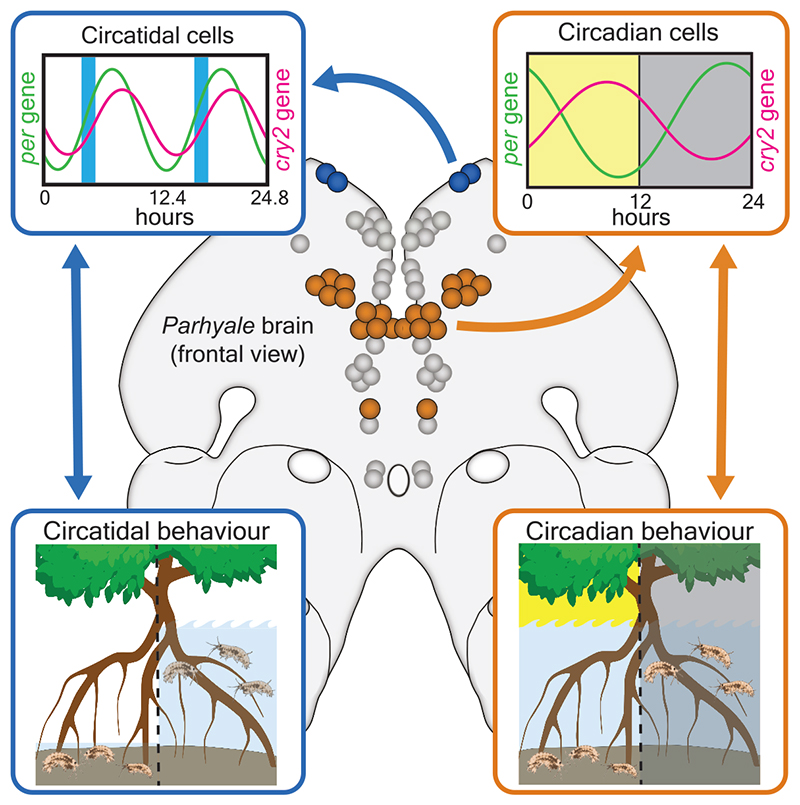

## Introduction

Organisms adapt to their cyclical environments through rhythms of metabolism, physiology, and behavior that are driven by internal biological clocks.^1^ Terrestrial environmental cycles are predominantly daily, leading to the evolution of clocks with an autonomous period of approximately 1 day (hence *circa*-*dian*) and entrained to the cycle of light and darkness. In contrast, coastal environments present greater temporal complexity and marine organisms have additionally evolved a range of circatidal (∼12.4 h), circasemilunar (∼14.8 days), and circalunar (∼29.5 days) timing mechanisms^2,3^ that co-ordinate adaptive rhythms across these time domains. Despite their biological relevance, the mechanistic underpinnings of circatidal clocks remain a mystery and three principal hypotheses have been proposed.^2^ First, circatidal and circadian rhythms are driven by a single oscillatory system, the period of which is plastic, adapting to the period of the dominant environmental cue.^4^ Second, paired circalunidian clocks with an intrinsic period of ∼24.8 h (i.e., a slightly modified circadian period) run in antiphase to create sequential peaks 12.4-h apart.^5^ Third, separate 12.4- and 24-h oscillators co-exist, independently driving circatidal and daily phenotypes.^6^ Testing these models requires identification of the clock cells and genes responsible for tidal timekeeping.

Circadian clocks in animals pivot around a transcriptional/translational feedback loop (TTFL) in which positive factors CLOCK (CLK) and BMAL1 (or CYC in flies), acting through E-box promoter sites, transactivate genes that encode negative regulators, PERIOD (PER), CRYPTOCHROME2 (CRY2), and/or TIMELESS (TIM), depending on species.^7^ Accumulating negative regulators progressively suppress transactivation and, following their degradation, the cycle restarts after ∼24 h.^8,9^ The intertidal peracarid crustaceans *Eurydice pulchra* (Isopoda, the speckled sea louse) and *Parhyale hawaiensis* (Amphipoda) can express autonomous circadian and/or circatidal rhythms of locomotor behavior in the absence of environmental cues.^10–13^ However, the only molecular markers of circatidal timekeeping relate to mitochondrial gene expression and cellular redox state in *E. pulchra*.^14^ Nevertheless, genes encoding CLOCK, BMAL1, PER, and CRY2 (i.e., invertebrate type 2 CRY) are present in both species, although the photosensitive invertebrate type 1 CRY is absent from both.^12,15^ Furthermore, *tim* expression in the head of *E. pulchra* is circadian,^12,16^ whereas *P. hawaiensis* lacks *tim*.^15^ *E. pulchra* CLOCK:BMAL1 heterodimers can transactivate E-boxes and are suppressed by PER and CRY2,^12^ whereas PhBMAL1 co-expressed with mouse CLK can also transactivate E-boxes.^13^ Finally, genomic deletion (*P. hawaiensis*) or RNAi-knockdown (*E. pulchra*) of *Bmal1* compromises tidal activity rhythms,^13,16^ raising the intriguing possibility of interactions between circadian and circatidal clocks at the molecular-genetic level.

The current aim was to describe the expression of *Bmal1, Clk, per*, and *cry2* (together with *Eptim*) across the brain to identify putative “clock” cells and then to test their relationship to daily, circadian, and circatidal behavior. We reveal ∼60 such cells arranged in multiple, discrete cell groups in the brain and show that particular groups are capable of tracking daily/circadian time (CT), encoded as the rhythmic expression of canonical circadian genes. Furthermore, we also identify cells with 12-h rhythms of expression of *tim* in *E. pulchra* and of *per* and *cry2* in *P. hawaiensis* and show that the latter are capable of tracking circatidal time. These results implicate canonical circadian mechanisms in circatidal timekeeping. They also reveal brain networks that may represent neural substrates for the generation of interactive daily and tidal rhythms appropriate to local intertidal habitats.

## Results

### The brains of *E. pulchra* and *P. hawaiensis* express canonical circadian clock genes

Our neuroanatomical descriptions of *E. pulchra* are based on the isopod *Saduria entomon*.^17^ The brain structure of *P. hawaiensis* has been described.^18,19^ To map gene expression across the brain, we first generated reference brains for *E. pulchra* and *P. hawaiensis* by averaging confocal image stacks of brains immunolabeled with anti-synapsin (SYNORF1) to reveal neuropils (Figure S1). We segmented the neuropils to facilitate description of gross brain anatomy and naming of clock cell groups relative to the body axis (Figures S1 and S2; Videos S1 and S2).

*Bmal1* is implicated in circadian and circatidal timekeeping in both *E. pulchra* and *P. hawaiensis*. We therefore used whole-mount HCR-FISH to map *Bmal1* transcripts as a first step to identify putative clock cells. This revealed widespread expression across the brain of *E. pulchra* (Figure S3A) and, consistent with qPCR studies,^12^ its intensity did not change over time in light:dark (LD) (Figure S4A). The expression of *Clk* mRNA was similarly widespread in the brain of *E. pulchra*, with most cells expressing low levels although there was a condensation of higher-intensity signals in ∼60 cells, located principally in the median protocerebrum (Figure S3B). These unexpected, dispersed *Bmal1* and *Clk* expression patterns in *E. pulchra* were confirmed using an alternative RNA detection technology, RNAscope, on wax-embedded brain sections (Figure S5). There were no differences in the expression patterns of *EpBmal1* (dispersed), *EpClk* (dispersed with some cellular enrichment), and *Epper* (cellular enrichment, see below) detected by the two methods, but the HCR-FISH on whole-mounts was a more practical and convenient approach and so it was adopted for further studies.

Pan-cellular expression of *Bmal1* was also evident in the brain of *P. hawaiensis* (Figure S3C) and, again, this did not change across the LD cycle (Figure S4B). In contrast, the expression of *Clk* in *P. hawaiensis* was less widespread than in *E. pulchra*, with enrichment in cells in the median protocerebrum (Figure S3D). The enrichment was clearer than in *E. pulchra* and these enriched cells were comparable in number and location to those in *E. pulchra*. Thus, notwithstanding the requirement for *Bmal1* to sustain circatidal behavior in both species,^13,16^ the pattern of its expression did not specifically signpost any populations of putative clock cells. In contrast, the expression of *Clk* was significantly elevated in discrete cell populations in the median protocerebrum of both species.

We then mapped the expression of genes encoding negative regulators *per, cry2*, and *tim* across the brain of *E. pulchra*. This revealed strong, specific signals for all three transcripts restricted to discrete cell clusters in the median protocerebrum (Figures S3E–S3G). In *P. hawaiensis* brains, the expression of *per* and *cry2* exhibited a pattern similar to that of *E. pulchra*, with high levels of both transcripts localized to ∼60 cells in the median protocerebrum (Figures S3I and S3J). The HCR-FISH was specific, insofar as probes against *deGFP* gave no signal (Figures S3H and S3K).

### Co-expression of circadian clock genes defines groups of putative clock cells in *E. pulchra* and *P. hawaiensis* brains

**Figure 1:**
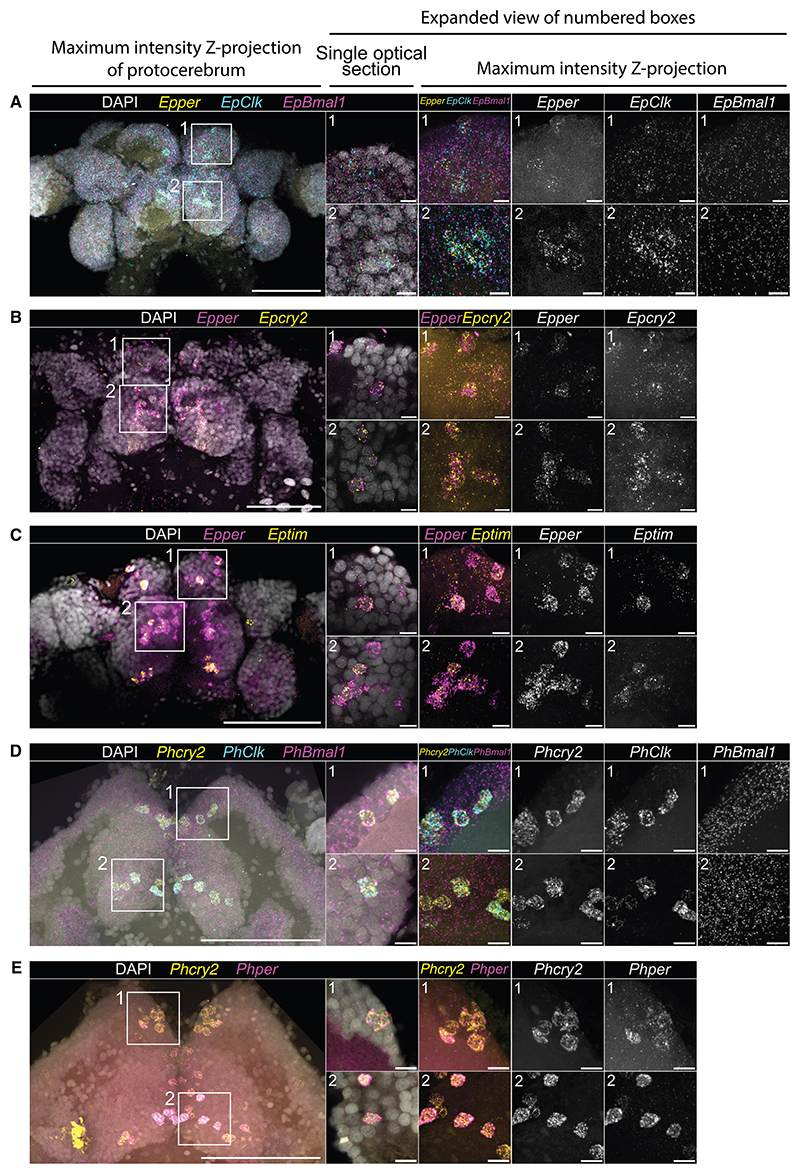
Cellular co-expression of circadian clock genes in the brains of *E. pulchra* and *P. hawaiensis* (A) Representative maximum intensity Z-projections and single optical sections of *E. pulchra* brain probed for *Epper, EpClk*, and *EpBmal1* using HCR-FISH. Left: merged low-power (25×) view of protocerebrum of *E. pulchra*, center: merged high-power (40×) single optical sections in boxes labeled 1 and 2, right: single channel high-power maximum intensity Z-projections. *EpBmal1* is widely and uniformly expressed in both putative clock cells and other cells across the brain, but it is not enriched in cells enriched for *EpClk*, whereas *Epper* is enriched in *EpClk*-enriched cells. (B) As in (A) for *Epper* and *Epcry2*, expression of which is co-enriched in putative clock cells but not in other cells in the brain of *E. pulchra*. (C) As in (A) for *Epper* and *Eptim*, of *Epper*-enriched cells in *E. pulchra*. (D) As in (A) but for *P. hawaiensis* brain probed for *PhBmal1, PhClk*, and *Phcry2* using HCR-FISH. *PhBmal1* is widely and uniformly expressed in both putative clock cells and other cells across the brain, but it is not enriched in cells enriched for *PhClk*, whereas *Phcry2* is enriched in *PhClk*-enriched cells. (E) As in (A) but for *P. hawaiensis* brain labeled for *Phcry2* and *Phper. Phcry2* and *Phper* are co-enriched in putative clock cells, but not other cells, in the brain. Animals were sampled across various points in the light phase of a 14L:10D (*E. pulchra*) or 12L:12D (*P. hawaiensis*) cycle in the laboratory without reference to activity rhythms. Scale bars: 100 μm (left-most), 10 μm (other). See also Figures S1–S6 and Table S1.

**Figure 2:**
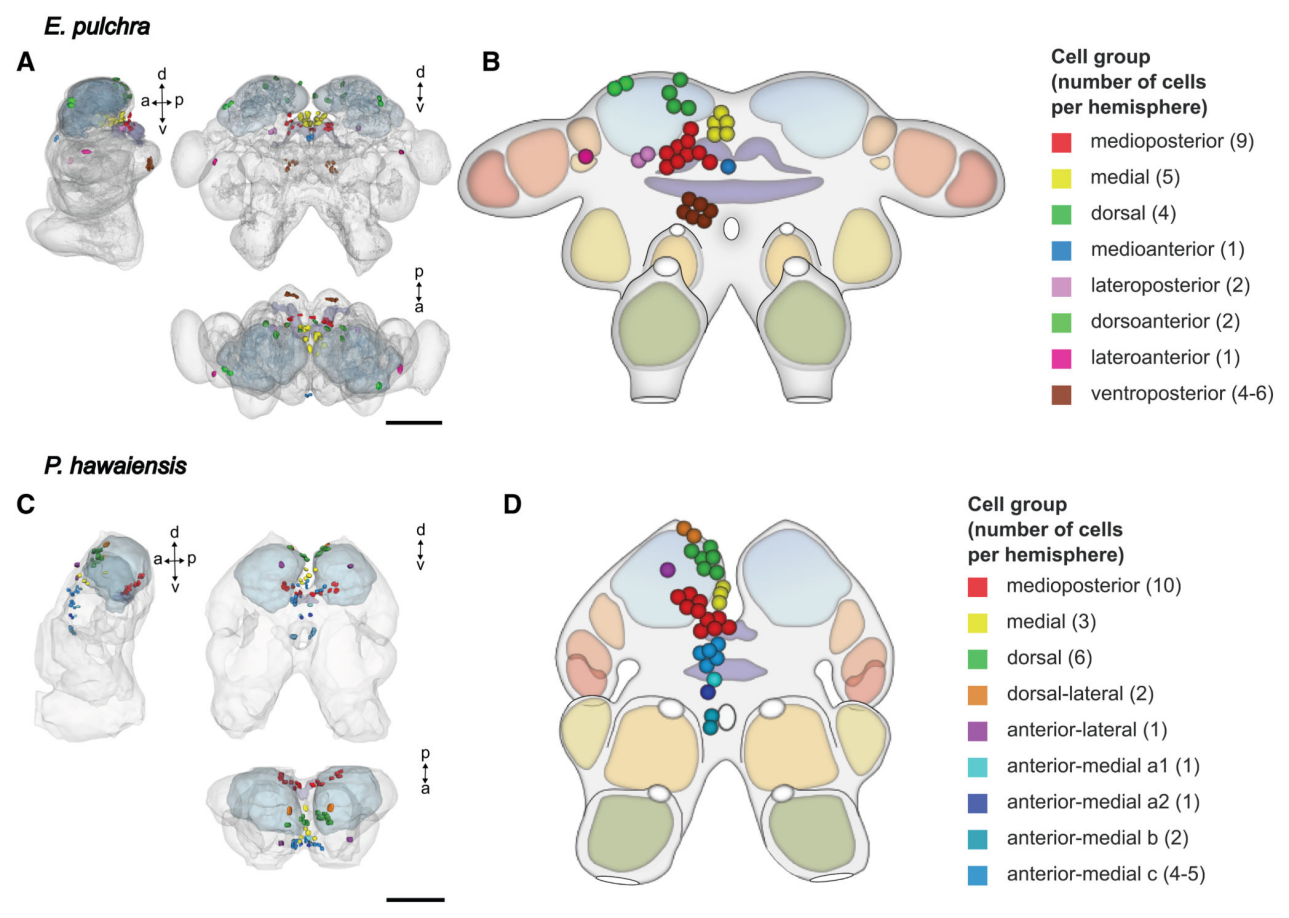
Putative clock cells in the brains of *E. pulchra* and *P. hawaiensis* revealed by co-expression of canonical circadian clock genes (A) Lateral, frontal, and dorsal views of the *E. pulchra* reference brain model (from Figure S2A) on to which somata enriched for expression of *Epper* and *Epcry2* are registered and segmented. The neuropils used in Figure S2 to signpost putative clock cells are here made translucent. (B) Diagrammatic representation of the *E. pulchra* brain summarizing the locations of cells co-enriched for *Epper* and *Epcry2*, based on the 3D reconstruction in (A). (C) Lateral, frontal, and dorsal views of the *P. hawaiensis* reference brain model (from Figure S2C) on to which somata enriched for expression of *Phper* and *Phcry2* are registered and segmented. The neuropils used in Figure S2 to signpost putative clock cells are here made translucent. (D) Diagrammatic representation of the *P. hawaiensis* brain summarizing the locations of cells co-enriched for *Phper* and *Phcry2* based on the 3D reconstruction in (C). Compass markers in (A) and (C) show anterior (a), posterior (p), dorsal (d), and ventral (v) directions. Discrete cell groups are color coded (see key). Cell numbers within each group (per hemisphere) in parenthesis. Scale bars: 100 μm. See also Figures S1–S6, Table S1, and Videos S1 and S2.

CLOCK:BMAL1 heterodimers are required for transactivation within the TTFL and so we compared the co-expression of *Bmal1* and *Clk* by multiplexed HCR-FISH of *E. pulchra* brains dissected in the light phase. The *Clk*-enriched cells expressed *Bmal1* at the low level seen in other cells across the brain. They also expressed high levels of *per* (Figure 1A) and the *per*-expressing cells also expressed *cry2* and *tim* (Figures 1B and 1C). Given that *per* and *cry2* encode the principal negative regulators of E-box-mediated transcription in *E. pulchra*,^12^ this small population of cells in the median protocerebrum co-expressed the full complement of genes required for a functional TTFL, highlighting them as putative clock cells. Using a similar approach in *P. hawaiensis*, we found, again, that *Clk*-enriched cells expressed *Bmal1* and high levels of *cry2* (Figure 1D). Furthermore, the *cry2*-enriched (and hence, *Clk*-enriched) cells also expressed *per* (Figure 1E), indicating that they are likely TTFL-competent cells. By combining HCR-FISH with anti-synapsin (SYNORF1) immunofluorescence (Figure S6; Videos S1 and S2), the putative clock cell clusters were registered to the reference brains (Figure 2). The groups were named by general location: 8 groups in *E. pulchra* and 9 in *P. hawaiensis*, with a total of ∼30 cells per hemisphere, distributed symmetrically. Cell groups and their given nomenclature are summarized in Table S1 and their distribution is described in Figure S6. Having defined the neuroanatomical distribution of putative clock cells, we then explored their capacity for rhythmic expression of clock genes and asked whether any of them can track daily or tidal time?

### Circadian clock genes are expressed rhythmically in putative clock cells of *E. pulchra* brain

**Figure 3:**
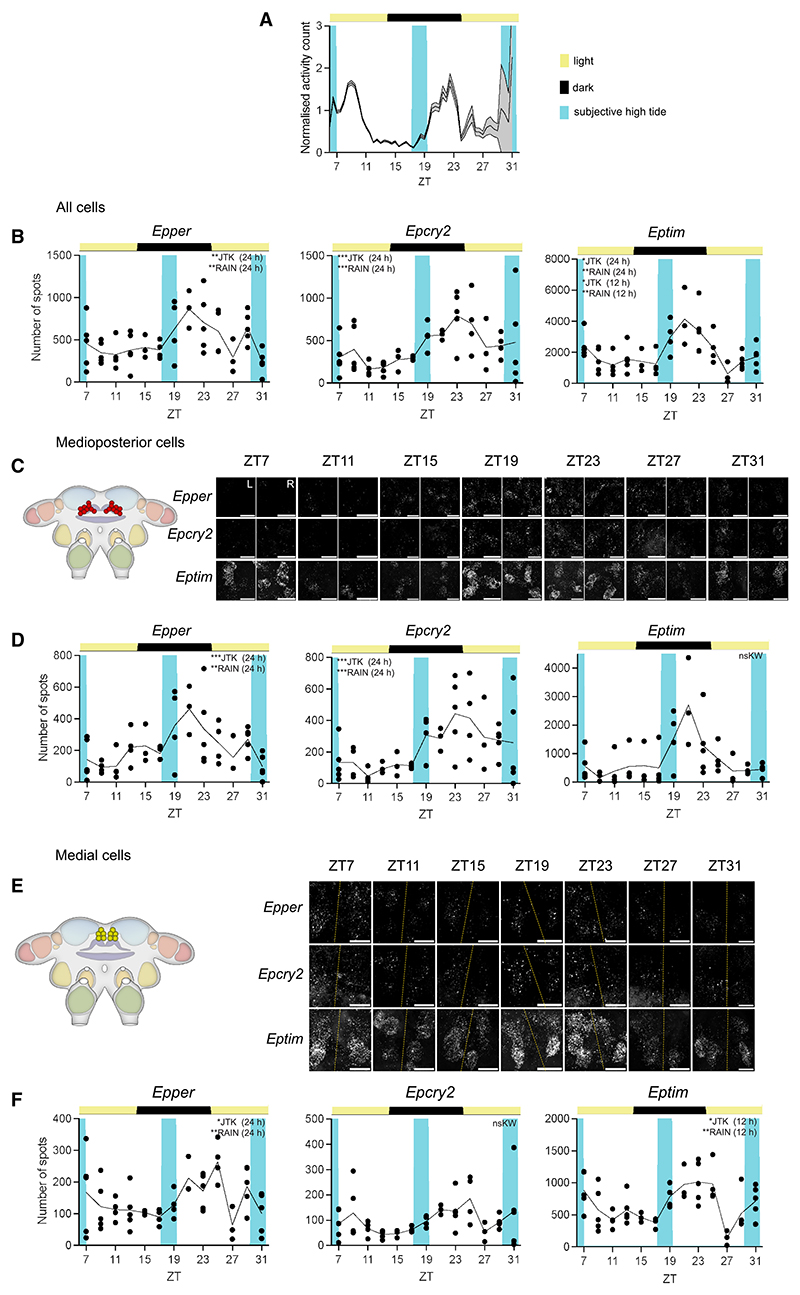
Time course of expression of *per, cry2*, and *tim* in cells of tidally rhythmic *E. pulchra* held on a LD cycle (A) Normalized swimming activity (mean + SEM, shading) of beach-collected *E. pulchra* harvested under LD (16L:8D) for the HCR-FISH time course (initial group size at start of harvest at ZT7 = 65). See also Figure S7. (B) Mean aggregate transcript abundance, quantified as the number of FISH spots, of *Epper, Epcry2*, and *Eptim* across all cells plotted across time in beach-collected animals held under LD. (C) Left: cartoon to show location of medioposterior cell group in the brain. Right: representative maximum intensity Z-projections of *Epper, Epcry2*, and *Eptim* expression in both hemispheres (L: left, R: right) across ZT (individual HCR-FISH channels in rows). Scale bars: 20 μm. (D) Mean transcript abundance, quantified as the number of FISH spots, of *Epper* and *Epcry2* in medioposterior cell group plotted across time in beach-collected animals held under LD. (E and F) As (C) and (D) but for medial cells group. Yellow dashed lines: midlines. Scale bars: 10 μm. Each point is from one brain, total across hemispheres. *n* = 3–5, *N* = 55. Inset text indicates statistical tests (JTK-cycle and RAIN) performed to determine 24- and 12-h rhythmicity, with the tested periods in parentheses, following significant time effect by ANOVA or Kruskal-Wallis. ***p < 0.001, **p < 0.01, *p ≤ 0.05, ^ns^*p* > 0.05. See also Figure S8 for clock gene expression in lateroposterior cell group. For statistics and data shown in this figure, refer to Table S2. See also Figure S15 and Table S10.

When placed into free-running conditions (DD) in the laboratory, field-collected *E. pulchra* displayed strong circatidal activity rhythms, with peak activity corresponding to the expected time of high water on the home beach (Figure S7). Across 11 independent field collections, 54.5% ± 6.5% of animals were tidally rhythmic (range: 20%–76%), with a mean period of 12.36 ± 0.02 h (±SEM, *N* = 478). To test whether some or all putative clock cells of *E. pulchra* can track daily or tidal time, we performed whole-mount, multiplexed HCR-FISH on dissected brains of circatidally rhythmic beach-collected *E. pulchra* sampled every 2 h under LD (16L:8D) for 24 h (Figure 3A). The number of HCR-FISH spots was used to quantify transcript abundance, and we considered expression to be rhythmic only when Kruskal-Wallis/ANOVA identified a significant time effect and both JTK_CYC^20^ and RAIN^21^ were significant (*p* < 0.05) at a period of 12 or 24 h for circatidal and daily rhythms, respectively. The aggregated counts across all cells, as a measure of total brain expression, showed strong daily rhythms for *per, cry2*, and *tim*, with peak levels in the late dark phase (Figure 3B). Aggregate levels of *tim* also showed a statistically significant 12-h rhythm, although the overall profiles for aggregate *per, cry2*, and *tim* were similar and most clearly tracked daily time.

To explore whether circatidal and circadian behavioral phenotypes are generated by different cell populations that contain ∼12- and ∼24-h molecular timers, we analyzed clock gene expression at the level of individual cell groups. Of the 7 groups examined, 5 showed significant rhythms of one or multiple transcripts (Table S2). Daily patterning was evident for *per* and *cry2* expression in the medioposterior cells, with peaks in late night (Figures 3C and 3D), whereas medial cells had a daily rhythm of *per* but a 12-h rhythm of *tim* (Figures 3E and 3F). Lateroposterior cells also had a significant 12-h cycle of *tim* (Figure S8), whereas *per* and *cry2* levels in these cells were not significantly rhythmic. In summary, in circatidally behaving *E. pulchra* sampled under LD, aggregate expression of *per* and *cry2* showed a daily rhythm, whereas aggregate levels of *tim* were more complex, reflecting both daily and circatidal time. When examined across distinct cell groups, daily cycles of *per* and *cry2* were evident in 4 of 7 groups, whereas a 12-h, putatively circatidal, pattern of *tim* expression was detected in two. Thus, distinct cell groups in *E. pulchra* appear able to track either daily or tidal time.

### Circadian and circatidal expression of clock genes in separate cell groups of *P. hawaiensis* brain

To establish circatidal behavioral rhythms, *P. hawaiensis* laboratory-reared under a 12L:12D cycle were entrained by a cycle of mechanical agitation for 2 h every 12.4 h for >30 days. On transfer to free-running conditions of DD and no further agitation, they showed circatidal rhythms with peak activity phased to anticipated agitation (i.e., subjective high tide) (Figures S9A and S9B). Across 13 experiments, 52.8% ± 2.8% of animals showed circatidal behavior (range 40%–70%) with a mean period of 12.40 ± 0.02 h (±SEM, *N* = 456) (Figures S9C and S9D). To confirm that the circatidal behavior was phased by the agitation and not by the LD cycle, a sub-group of animals were subjected to a phase-shifted regime for the final 6 days of entrainment in which agitation occurred every 12.9 h rather than every 12.4 h as in matched controls. It therefore accumulated a relative delay of ∼6 h (0.5 h per cycle over ∼12 cycles). On release to DD, control and phase-shifted animals exhibited circatidal behavioral rhythms with peak activity at projected high tide and periods of ∼12.4 h that were not significantly different between groups (Kolmogorov-Smirnov test: *D* = 0.1779, *p* = 0.59, n.s.) (Figures S9E–S9G). Over four replications of the control and shifted regimes, their effectiveness (45.7% vs. 49.8% circatidal animals) was comparable (paired t test: t = 0.27, df = 3, *p* = 0.80, n.s., Cohen’s d = 0.136). Importantly, the phase of the circatidal behavior was delayed by 4.94 ± 0.51 h (±SEM, *N* = 4) in phase-shifted animals relative to the controls (Figures S9E, S9F, and S9H). This not only confirms the capacity of *P. hawaiensis* to exhibit circatidal behavior^13^ but also demonstrates that the circatidal clockwork of *P. hawaiensis* is sensitive to mechanical agitation, as is the case for *E. pulchra*.^11,12^

**Figure 4:**
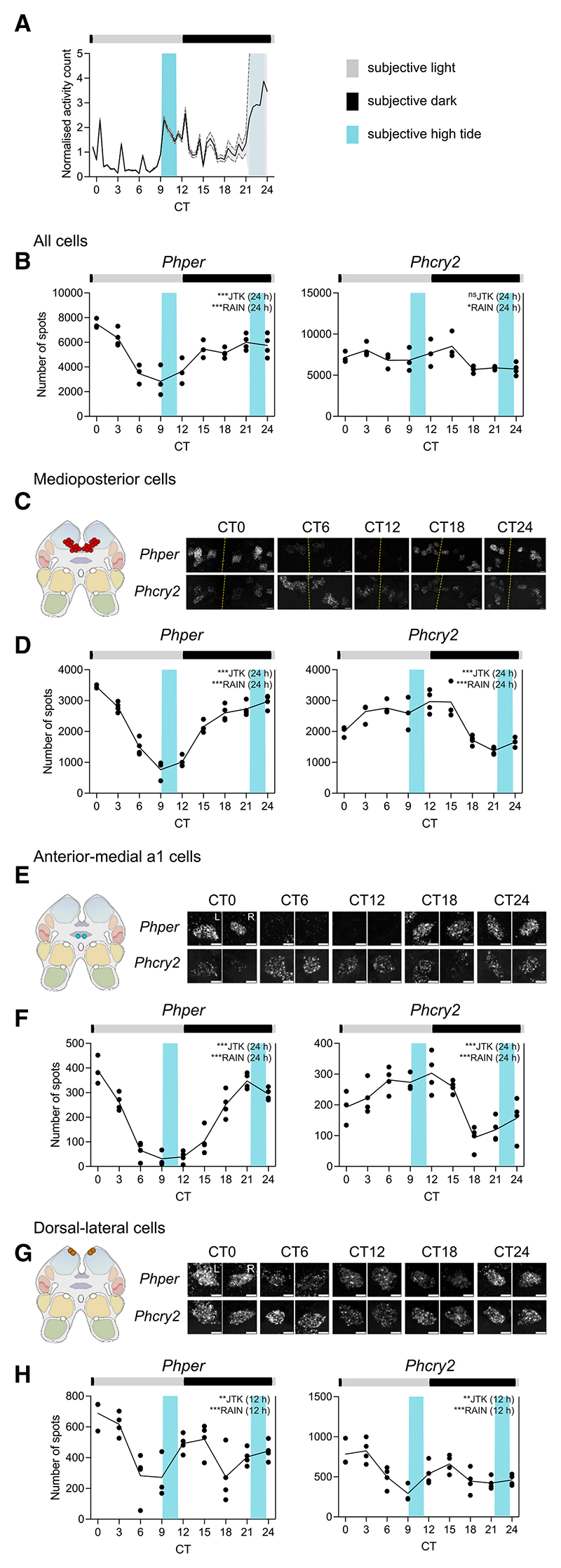
Free-running clock gene expression rhythms in tidally entrained *P. hawaiensis* (tidal phase θ) (A) Normalized swimming activity (mean + SEM, shading) of *P. hawaiensis* synchronized to the prior agitation cycle and harvested for the HCR-FISH time course (initial group size at start of harvest at CT0 = 37). (B) Mean aggregate transcript abundance, quantified as the number of FISH spots, of *Phper* and *Phcry2* across all putative clock cells plotted across time in tidally entrained animals sampled under DD. *n* = 3–4, *N* = 31. (C) Left: cartoon to show location of medioposterior cell group in the brain. Right: representative maximum intensity Z-projections of *Phper* and *Phcry2* expression in both hemispheres (yellow dashed lines: midlines) across CT (individual HCR-FISH channels in rows). (D) Plots of mean transcript abundance, quantified as the number of FISH spots, across CT for *Phper* (left) and *Phcry2* (right). *n* = 3–4, *N* = 33. (E and F) As in (C) and (D) for anterior-medial a1 cells. *n* = 3–4, *N* = 34. L, left; R, right. (G and H) As in (C) and (D) for dorsal-lateral cells. *n* = 3–4, *N* = 34. L, left; R, right. Each point is from one brain, total across hemispheres. Inset text indicates statistical tests (JTK-cycle and RAIN) performed to determine 24- and 12-h rhythmicity, with the tested periods in parentheses, following significant time effect by ANOVA or Kruskal-Wallis. ***p < 0.001, **p < 0.01, *p ≤ 0.05, ^ns^*p* > 0.05. For statistics shown in this figure, refer to Table S3. Scale bars: 10 μm (C), 5 μm (E and G). See also Figures S9 and S15 and Tables S7 and S10.

To test whether cells in *P. hawaiensis* could track circadian and/or tidal time, whole-mount HCR-FISH was applied to the brains of animals entrained by tidal agitation and exhibiting circatidal activity before harvest (Figure 4A). Aggregate expression of *per* was circadian, peaking in subjective night, whereas aggregate expression of *cry2* did not vary with time (Figure 4B). Across the 10 cell groups examined, 3 groups showed significant rhythms of one or multiple transcripts (Table S3). Notably, expression of *per* was strongly circadian in medioposterior cells and anterior-medial a1 cells (Figures 4C–4F). Moreover, these cells also showed circadian cycles of *cry2* expression, running in antiphase to *per*. Thus, *per* and *cry2* expression in these cells tracked CT rather than tidal time in circatidally rhythmic animals. In contrast, dorsal-lateral cells in the same animals showed a significant 12-h cycle of *per* and *cry2* transcripts (Figures 4G and 4H). Furthermore, in the dorsal-lateral cells, unlike the circadian cells, *per* and *cry2* oscillated almost in phase, both peaking after subjective high tides (∼CT00-03 and CT15).

**Figure 5:**
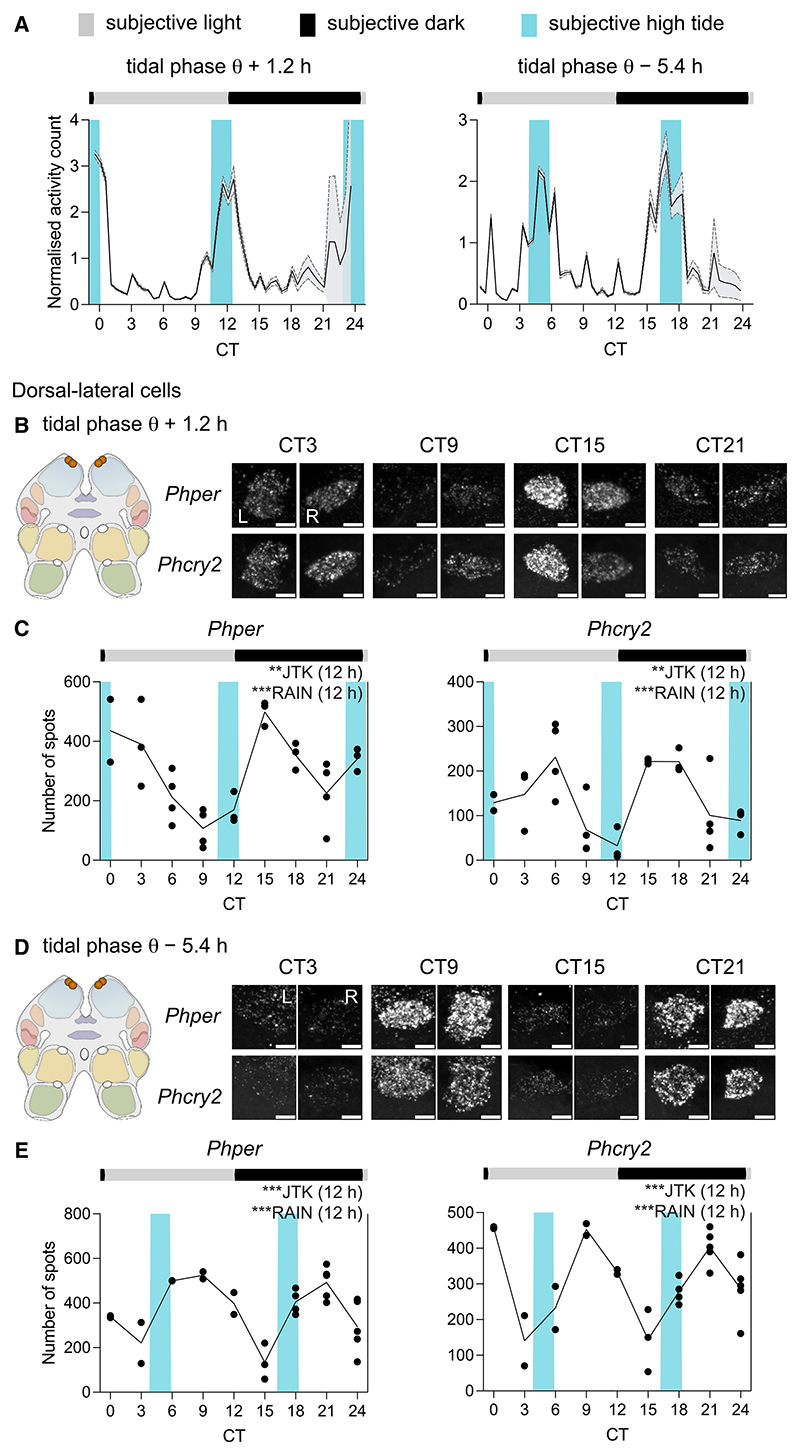
Circatidal rhythms of expression of *Phper* and *Phcry2* in dorsal-lateral cells in the brain of tidally entrained *P. hawaiensis* (A) Normalized swimming activity (mean + SEM, shading) of *P. hawaiensis* synchronized to the prior agitation cycle, phase-shifted by +1.2 h (left) or –5.4 h (right) and harvested for the HCR-FISH time course (initial group sizes at start of harvest at CT0 = 35). (B) Left: cartoon to show location of dorsal-lateral cell group in the brain. Right: representative maximum intensity Z-projections of *Phper* and *Phcry2* in both hemispheres (L, left; R, right) across CT (individual HCR-FISH channels in rows). Scale bars: 5 μm. (C) Plots of mean transcript abundance, quantified as the number of FISH spots, across CT for *Phper* (left) and *Phcry2* (right) (blue shadings: subjective high water). *n* = 2–4, *N* = 29. (D and E) As in (B) and (C) for animals entrained to a tidal cycle advanced, relative to the LD cycle, by 5.4 h. *n* = 2–5, *N* = 27. Each point is from one brain, total across hemispheres. Inset text indicates statistical tests (JTK-cycle and RAIN) performed to determine 24- and 12-h rhythmicity, with the tested periods in parentheses, following significant time effect by ANOVA or Kruskal-Wallis. ***p < 0.001, **p < 0.01, *p ≤ 0.05, ^ns^*p* > 0.05. For statistics and data of FISH time course experiments on tidally entrained animals, refer to Tables S4 and S5 for tidal phases θ + 1.2 and θ − 5.4 h, respectively. See also Figures S10, S11, S12, and S15 and Tables S7 and S10.

The experiment was repeated, with the final tidal agitation occurring slightly later (+1.2 h) relative to the LD cycle. The animals again exhibited robust circatidal activity rhythms in DD, phased to anticipated high water (Figure 5A). Aggregate expression of *per* was circadian (Figure S10A) and, across the 10 cell groups examined, the same three groups (medioposterior, anterior-medial a1, and dorsal-lateral) showed significant rhythms of one or multiple transcripts (Table S4). The expression of *per* and *cry2* in the medioposterior and anterior-medial a1 cells was circadian (Figures S10B–S10E), whereas the dorsal-lateral cells again showed a strong circatidal, 12-h pattern of expression of *per* and *cry2*. The transcripts were also oscillating in phase and peaking after subjective high tide (Figures 5B, 5C, and S10F). To test whether we could phase-shift these putative tidal cells even further, and to differentiate them from light-entrained circadian cells exhibiting bimodal 24-h rhythms, we entrained animals such that the final bout of tidal agitation occurred 5.4 h phase-shifted relative to the 12:12LD cycle (i.e., subjective high tides at ∼CT3.8 and ∼CT16.2). On release into constant conditions, the animals exhibited appropriately phased circatidal rhythms of activity peaking at subjective high tide (Figure 5A). The aggregate expression of *per* (but not *cry2*) was circadian, peaking in subjective night (Figure S11A). Of the 10 cell groups examined, 4 (the original three plus anterior-lateral cells) showed an effect of time on transcript levels (Table S5). Medioposterior cells showed antiphasic circadian rhythms of *per* and *cry2*, anterior-medial a1 cells showed a circadian cycle of *per* (Figures S11B–S11E), and anterior-lateral cells were circadian for *cry2*. In contrast, the dorsal-lateral cells again showed 12-h cycles of expression of *per* and *cry2* (Figures 5D, 5E, and S11F). Moreover, the peaks occurred after subjective high tide and, therefore, the profiles were phase-shifted relative to the prior LD cycle by ∼6 h when compared with the previous tidal experiments. Comparison across the three experiments with circatidally active animals showed conservation of the phase relationship between *per* and *cry2* expression across studies as well as adoption of the phase of the prior regime of tidal agitation (Figure S12). Thus, in the same brains of *P. hawaiensis* exhibiting free-running circatidal behavior, medioposterior and anterior-medial a1 cells consistently tracked CT, expressed by antiphasic rhythms of *per* and *cry2* expression, whereas the dorsal-lateral cells consistently tracked tidal time, independently of CT, and did so with *per* and *cry*2 rhythms in phase.

### Loss of circatidal but not daily expression of clock genes in putative clock cells of *P. hawaiensis* under a LD cycle

To test whether dorsal-lateral cells have any capacity to follow daily time, we entrained *P. hawaiensis* to LD cycles without tidal agitation. As reported,^10,13^ *P. hawaiensis* exhibited rhythmic activity under LD, peaking at night (Figures S13A, S13B, S13D, and S13E). Across 7 experiments 58.6% ± 4.4% were rhythmic (range: 45%–80%, total *N* = 189). When released into constant darkness, ∼50% of rhythmic animals (i.e., 22.9% ± 7.5% of total) showed circadian rhythms in their locomotor activity (Figures S13A, S13C, S13D, and S13F), with a mean (±SEM) period of 24.17 ± 0.17 h (*N* =39). Activity levels were significantly higher during the subjective night than in the subjective day.

**Figure 6:**
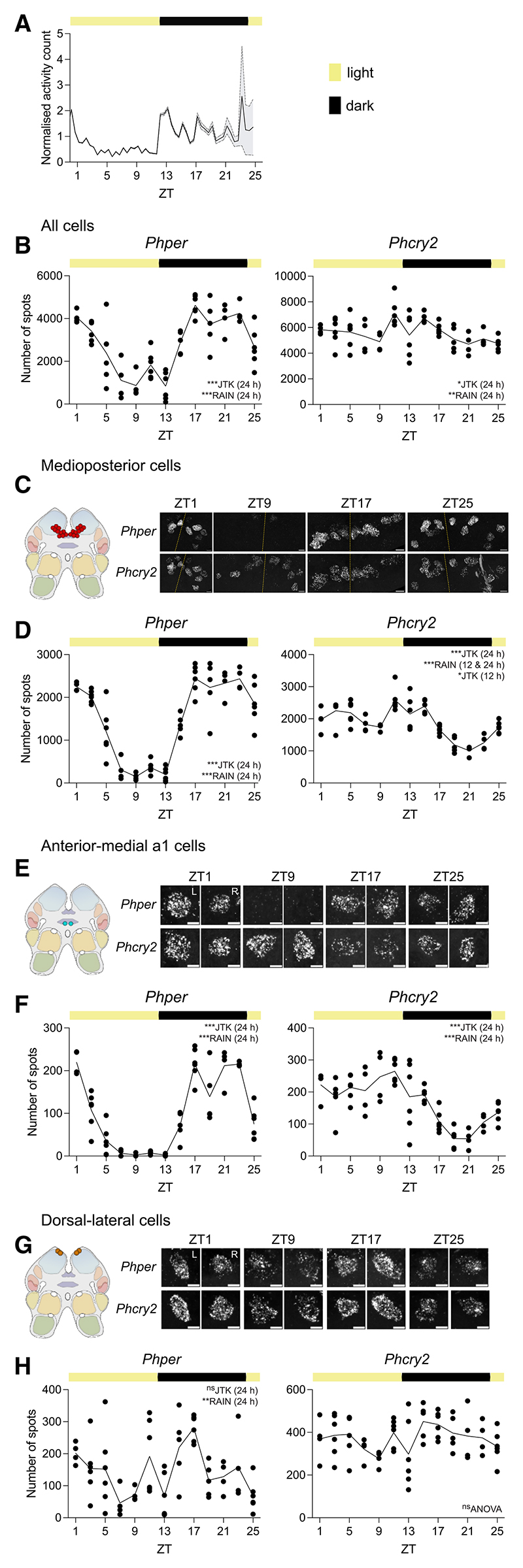
Time course of expression of *per* and *cry2* in cells of *P. hawaiensis* synchronized to the LD cycle (A) Normalized swimming activity (mean + SEM, shading) of *P. hawaiensis* synchronized to the LD cycle and harvested for the HCR-FISH time course (initial group size at start of harvest at ZT1 = 79). (B) Mean aggregate transcript abundance, quantified as the number of FISH spots, of *Phper* and *Phcry2* across all cells plotted across time in animals entrained to, and sampled under, a 12 h:12 h LD cycle. *n* = 4–6, *N* = 66. (C) Left: cartoon to show location of medioposterior cell group in the brain. Right: representative maximum intensity Z-projections of *Phper* and *Phcry2* expression in both hemispheres (yellow dashed lines: midlines) across ZT (individual HCR-FISH channels in rows). (D) Plots of mean transcript abundance, quantified as the number of FISH spots, across daily time for *Phper* (left) and *Phcry2* (right). *n* = 4–6, *N* = 66. (E and F) As for (C) and (D) for anterior-medial 1a cells. *n* = 4–6, *N* = 66. L, left; R, right. (G and H) As for (C) and (D) for dorsal-lateral cells. *n* = 4–6, *N* = 66. Each point is from one brain, totaled across hemispheres. Inset text indicates statistical tests (JTK-cycle and RAIN) performed to determine 24- and 12-h rhythmicity, with the tested periods in parentheses, following significant time effect by ANOVA or Kruskal-Wallis. ***p < 0.001, **p < 0.01, *p ≤ 0.05, ^ns^*p* > 0.05. See also Figure S14 for other cell groups with 24-h cycling in clock gene expression. For statistics and data shown in this figure, refer to Table S6. Scale bars: 10 μm (C), 5 μm (E and G). See also Figures S13 and S15 and Table S10.

In animals confirmed to be showing daily behavioral activity under 12L:12D prior to harvest of their brains for HCR-FISH (Figure 6A), the aggregate expression of *per* was highly rhythmic, peaking at night (Figure 6B). Aggregate expression of *cry2* was also significantly rhythmic, in antiphase to *per*, but with a small amplitude. Of the 10 cell groups examined, 5 groups showed a daily pattern of one or both transcripts (Table S6). In both the medioposterior cell group and the anterior-medial a1 cells, *per* and *cry2* were rhythmic and in antiphase, with nocturnal and diurnal peaks, respectively (Figures 6C–6F), echoing the circadian patterns seen in these cells in tidally active animals under DD. Rhythmic expression of *per* was also evident in the dorsal cells and the anterior-medial b cells (Figures S14A–S14D), again peaking in the night, and *cry2* was rhythmic in anterior-medial c cells. In contrast, there was no statistically significant rhythmic expression of either *per* or *cry2* in the dorsal-lateral cells of the daily-behaving animals (Figures 6G and 6H). Thus, several cell groups can exhibit daily cycles of gene expression in daily-behaving *P. hawaiensis* under LD, including the medioposterior and anterior-medial a1 cells that are also circadian under DD. In contrast, the circatidal gene expression observed in dorsal-lateral cells in tidally rhythmic animals under DD was specific to that condition: in the absence of tidal cues this population of cells did not show tidal patterning nor did they adopt the daily rhythms of expression seen in other cell groups under LD.

**Figure 7:**
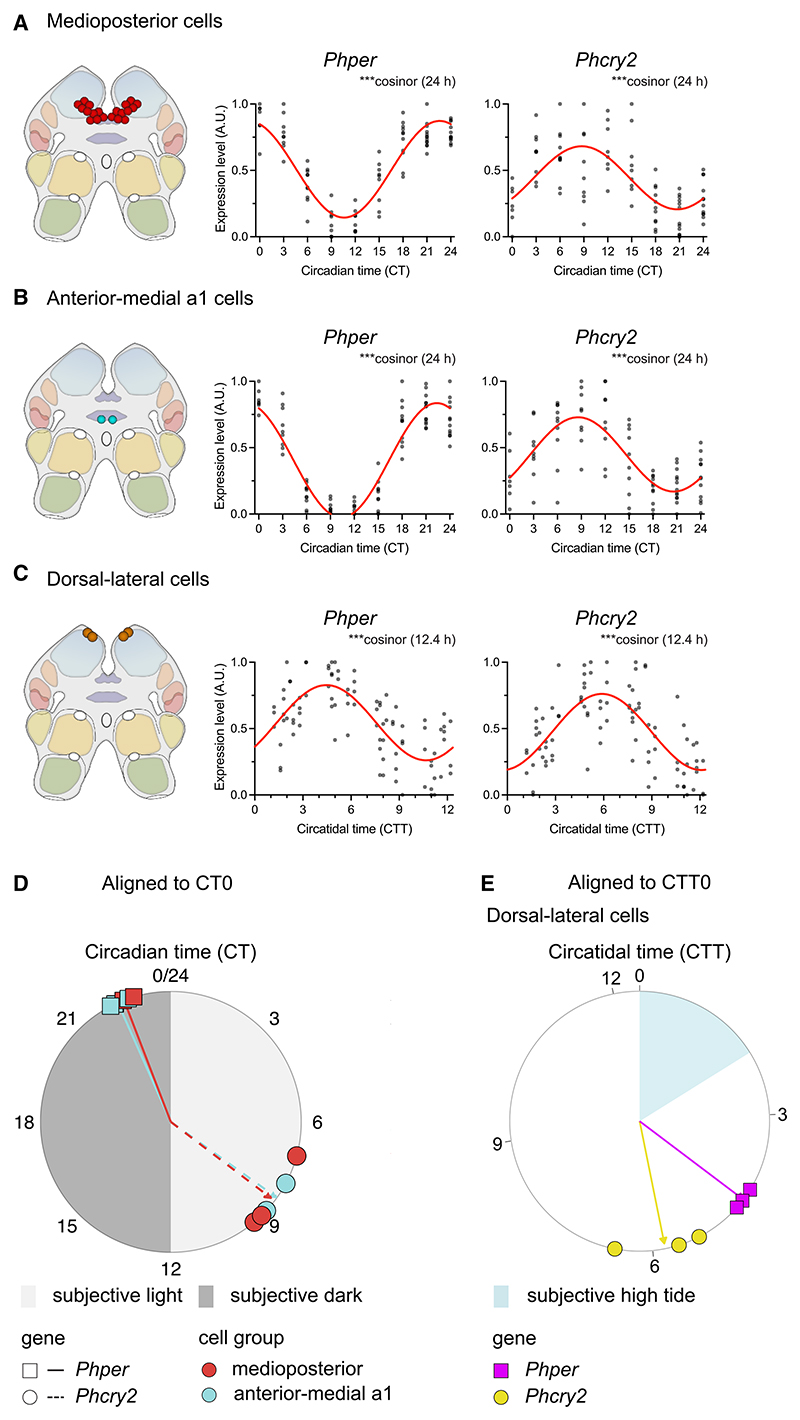
Summary meta-analysis of circatidal and circadian rhythms of clock gene expression in medioposterior, anterior-medial a1, and dorsal-lateral cell groups of *P. hawaiensis* (A) Left: cartoon to show location of the medioposterior cell group in the brain. Right: scatter-plot of expression of *Phper* (left) and *Phcry2* (right) across three independent experiments registered to subjective CT (CT = 0). Red lines indicate 24-h cosinor fits. (B) As in (A) for anterior-medial a 1 cell group. (C) As in (A) for dorsal-lateral cell group, but plots are registered to subjective high tide (circatidal time [CTT] = 0). Red lines indicate 12.4-h cosinor fits. Inset text in (A)–(C) indicate statistical significance of cosinor. (D) Circular plot of circadian peak expression phase of *Phper* and *Phcry2* in selected rhythmic cell groups of *P. hawaiensis* exhibiting circatidal activity rhythms. Each color-shape combination represents data from across *N* = 3 experiments. Arrowed lines indicate mean phase for each gene-cell group combination, and length of arrowed lines indicates the power of the vector at each mean. (E) Circular plots of circatidal peak expression phase of *Phper* and *Phcry2* in the dorsal-lateral cells of *P. hawaiensis* exhibiting circatidal activity rhythms, with the circatidal acrophase aligned to CTT0 within each individual experiment. Arrowed lines indicate mean phase for each gene-cell group combination, and length of arrowed lines indicates the power of the vector at each mean. For mean phase and mean vector lengths for each gene-cell group combination in (D) and (E), see Table S7.

The distinctions between circadian and circatidal rhythms of gene expression are evident in a combined meta-analysis across the three tidal entrainment studies, registered alternatively in either circadian or circatidal time (Figure 7). When examined across all experiments in the same circatidally behaving animals, the expression of *per* and *cry2* was highly circadian in the medioposterior cells and anterior-medial a1 cells (Figures 7A and 7B), with *cry2* oscillating in antiphase to *per*. On the other hand, the expression of *per* and *cry2* in dorsal-lateral cells was highly circatidal and co-phasic (Figure 7C). Circular plots registered to prior LD and tidal agitation emphasize these differences in periodicity and acrophase between the circadian and the circatidal cells (Figures 7D and 7E). Moreover, this mapping of the tidal oscillations to the phase of tidal entrainment in all three experiments confirmed that the dorsal-lateral cells were able to express a free-running molecular signature of tidal time that was independent of CT and revealed, thereby, that the brain of *P. hawaiensis* contains independent circadian and circatidally rhythmic cell populations.

## Discussion

Examination of the expression of canonical circadian clock genes across the brains of both *E. pulchra* and *P. hawaiensis* identified ∼60 cells co-expressing *Clk, Bmal1, cry2*, and *per* (and *tim* in *E. pulchra*), arranged into distinct groups across the protocerebrum. Although the loss of *Bmal1* compromises circatidal behavior in *E. pulchra*^16^ and in *P. hawaiensis*,^13^ its brain-wide expression showed no local enrichment and so did not highlight any potential circatidal clock cells. This broad distribution is, however, consistent with BMAL1 having diverse, pleiotropic actions beyond timekeeping.^22^ On the other hand, the observed enrichment of *Clk, per*, and *cry2* expression (alongside *tim* in *E. pulchra*) in specific cell groups indicated their potential function as circadian oscillators.

In circatidally active *E. pulchra* sampled under LD cycles, the aggregated signals for *per* and *cry2* were daily, reflective of their putative circadian roles, and both peaked in the late night, as expected for negative co-regulators within the TTFL. In contrast, aggregate brain expression of *tim* showed significant patterning not only at 24 h, consistent with circadian cycling of *tim* in whole heads assayed by qPCR,^12^ but also at 12 h. Analysis at the level of individual cell groups provided more granular insight, revealing 24-h rhythms of *per* and *cry2* in medioposterior cells and of *per* in medial cells, with peak expression in late night. This absence of circatidal rhythmicity of *per* expression resonates with the finding that knockdown of *per* to ∼20% of wild-type levels, had no effect on circatidal activity in *E. pulchra*, although it did disrupt circadian phenotypes.^12^ Alternatively, *tim* showed 12-h accumulation in both the medial and the later-oposterior cells, peaking ∼2 h after expected high water. These-cell-specific differences in *tim* expression likely underlie the compound periodicity evident in the aggregate expression. Furthermore, the 12-h patterns in the medial and the lateroposterior cells indicate potential tidal timekeeping in these cells associated with circatidal behavior.

In *P. hawaiensis*, aggregate expression of *per* tracked daily time under LD and CT under DD, with elevated levels during the dark phase and subjective night, respectively. Daily and circadian patterning were even more pronounced when examined in individual cell groups, with rhythmic expression of *per* evident in medioposterior, anterior-medial a1, dorsal, and anterior-medial b cells. Notably, whereas *per* and *cry2* were in phase in daily cells of *E. pulchra*, expression of *per* and *cry2* in daily and circadian cells in *P. hawaiensis* was antiphasic, suggesting an inter-species difference in the underlying molecular architecture of the circadian TTFL. This suggests that *cry2* and *per* may carry different circadian regulatory sequences to direct their specific phases, and future analyses of the *P. hawaiensis* and *E. pulchra* genomes may illuminate this mechanism. It also raises questions about protein levels; for example, are PER and CRY2 expressed in antiphase? A precedent comes from work in the mouse SCN, where the levels of mPER2 and mCRY1 proteins are temporally segregated, with mPER2 peaking ∼6 h before peak mCRY1.^23^

Remarkably, following entrainment of *P. hawaiensis* to artificial tidal cycles under LD and then subsequent transfer to free-running conditions, *per* and *cry2* expression in dorsal-lateral cells was robustly rhythmic, with a circatidal period that reflected the circatidal behavior of the animals. Across independent trials, these cells tracked experimental tidal time, regardless of prior LD phase. In contrast, clock gene expression in these cells was arrhythmic when analyzed across individuals sampled under LD without tidal cues and with daily, but not tidal, behavior. These observations place the dorsal-lateral cells as strong candidates for dedicated circatidal oscillators, especially when other cell groups in the same brains (e.g., medioposterior and anterior-medial a1 cells) ignored tidal cues and remained circadian, phased to prior LD. Notably, *per* and *cry2* in these tidal cells oscillated in phase, in contrast to the antiphasic rhythms of *per* and *cry2* in daily/circadian cells. The functional significance of these varying relationships, which are surprising in terms of the canonical TTFL whereby *per* and *cry2* are typically co-phasic, is not clear, but they suggest that a common molecular architecture may have diverged, evolving to operate under different time-bases in different cells. Of the canonical clock genes in *E. pulchra*, only *tim* expression was associated with tidal time in 2 of the 4 neuronal groups studied. Future analysis of protein expression in daily and tidal cell groups will better inform understanding of cell-specific TTFL structures. In addition, examination of clock gene expression in the different cell groups of animals with genetic deletion or knockdown of BMAL1 (and CLK) would indicate mechanistic bridges between the TTFL and tidal behavior. It is possible that the circatidal expression patterns of *Phper, Phcry2*, and *Eptim* are driven by feedback from a cryptic tidal clock that exists within, or even outside, those cells. Consequently, these patterns may reflect cellular activity but are not in themselves part of the clock nor are they causal to BMAL1-dependent tidal behavior. Such a scenario could explain why RNAi of canonical negative regulators had no effect on tidal behavior in *E. pulchra*.^12,16^ The genetic tractability of *P. hawaiensis* may enable this to be tested directly.

Notably, even though numerous cells oscillated at ∼24 h (22 in *P. hawaiensis*) and only a few at ∼12 h (4 in *P. hawaiensis*), both *E. pulchra* and tidally entrained *P. hawaiensis* were strongly circatidal in terms of behavior. This raises the question of why strong tidal phenotypes are reflected by relatively few tidal cells compared with the strong aggregate cycles of daily/circadian gene expression. Of course, day/night cycles are highly predictable in nature, and it is therefore expected that circadian systems will represent a prominent and robust feature. Nevertheless, coastal animals have evolved temporally complex behaviors to match their habitats, cued by multiple stimuli.^10–13^ It is therefore possible that gene expression may be circatidal in additional cell groups in *E. pulchra* and/or *P. hawaiensis*, but it evaded statistical detection in the current study because of the high variance intrinsic to population-based measures. Alternatively, different tidal cell groups may respond to distinct entraining cues and so only a subset of these groups is revealed in any single experimental setting. Examination of gene expression in the brain of *P. hawaiensis* entrained by tidal immersion/emersion,^13^ with and without tidal agitation, may indeed reveal additional putative circatidal clock cells: can subsets of cells we describe here as non-rhythmic be recruited by tidal stimuli of other modalities? Alternatively, is there a unique tidal role for dorsal-lateral cells? Such a cell- and stimulus-specific entrainment repertoire raises the question of how tidal stimuli are transduced to the central brain and whether a distributed multi-modal network broadens the capacity for tidal entrainment, reinforcing it in diverse natural settings.^2,10,11^

In considering the three hypotheses of circatidal time-keeping,^4,6,24^ our data are most consistent with the model of dedicated tidal and daily timers proposed by Naylor,^6^ with canonical circadian genes in distinct cell groups tuning to either circatidal or circadian periodicities. Our demonstration that dual circatidal and circadian systems operate in parallel echoes other work on circatidal clocks. For example, surgical removal of the optic lobes of the mangrove cricket, *Apteronemobius asahinai*, abrogates circadian but not circatidal locomotor phenotypes, indicative of two anatomically separable timers.^25,26^ In contrast, evidence in favor of a circadian/tidal timer based on a single “plastic” clock, as proposed by Enright,^4^ received support from studies on the coastal oyster *Crassostrea gigas*, which showed ∼12-h and/or ∼24-h expression of circadian gene homologs.^27^ Importantly, these models are not mutually exclusive. Given the evolutionary distance between mollusks and arthropods, it is entirely possible that tidal timekeeping machineries have evolved independently. In fact, a parsimonious explanation for the evolution of clock cells with different periodicities would be that pre-existing circadian clock components were co-opted to track relevant tidal cues. The cells we identified invariably co-expressed the rudiments of a circadian TTFL but with either circadian or circatidal frequencies in the same brain, and it is possible that they evolved in parallel, diverging under the influence of daily (e.g., photosensory) and tidal (e.g., mechanosenory) inputs, respectively.

Kwiatkowski et al.^10^ asked how *P. hawaiensis* “chooses” between 12.4- and 24-h environmental rhythms during entrainment. Our observation that distinct circadian and circatidal cell groups entrain independently to light and mechanical stimuli, respectively, provides a solution to this problem. Similarly, these authors also questioned whether neurons coordinating rhythmic behavior in *P. hawaiensis* have two different timers, with differential sensitivity to light and tides, or whether two cell types exist. Although we cannot currently exclude the former, our results align better with the latter. This does not preclude interactions of the two systems, however, and the genetic and behavioral malleability of *P. hawaiensis* should enable their decipherment. Thus, our findings may facilitate the examination of input and output pathways of tidal and daily cues at cellular resolution, progressing toward defining the nature and connectivity of circadian and circatidal neural clockworks. In conclusion, our results implicate canonical circadian mechanisms in circatidal timekeeping and reveal brain networks that may represent neural substrates for the generation of interactive daily and tidal rhythms.

## Resource Availability

### Lead contact

Requests for further information, resources and code should be directed to, and will be fulfilled by, the lead contact, Michael H. Hastings (mha@mrc-lmb.cam.ac.uk).

### Materials availability

This study did not generate new unique reagents.

## Star★Methods

### Key Resources Table

**Table:** 

REAGENT or RESOURCE	SOURCE	IDENTIFIER
Antibodies		
3C11 (anti SYNORF1)	Developmental Studies Hybridoma Bank (DSHB)	RRID: AB_528479
anti-mouse Alexa 488	ThermoFisher	Cat#A-21141
F(ab′)2-goat anti-mouse IgG-Alexa Fluor 488	ThermoFisher	Cat#A-11017
Alexa Fluor Plus 555	ThermoFisher	Cat#A48287
Fab goat anti-mouse IgG-Alexa Fluor 488	Jackson ImmunoResearch	Cat#115-547-003
Chemicals, peptides, and recombinant proteins		
Sea salt	Reef Salt, Tropical Marine Centre	N/A
RapiClear 1.49	SunJin Lab Optical Clearing Innovation	Cat#RC149001
DPX	Sigma Aldrich	Cat#06522
Prolong Glass Antifade Mountant	ThermoFisher	Cat#P36980
RNase inhibitor	New England BioLabs	Cat#M0314L
RNaseOUT™ ribonuclease inhibitor	Invitrogen	Cat#10777019
RNAscope® Multiplex Fluorescent Reagent Kit v2 Assay	ACD Biotechne	Cat#323100
Vectashield® Antifade mounting fluid	Vector Laboratories, 2B Scientific	Cat#H-1000-10ML
HCR™ Probe Hybridization Buffer	Molecular Instruments Inc.	LOT: BPH03725
HCR™ Probe Wash Buffer	Molecular Instruments Inc.	LOT: BPW03026
HCR™ Amplification Buffer	Molecular Instruments Inc.	LOT: BAM03526
Leica Paraplast Plus	Leica Biosystems	39602004
Experimental models: Organisms/strains		
*Eurydice pulchra,* wild caught		N/A
*Parhyale hawaiensis,* Chicago-F strain	Aziz Aboobaker	N/A
Oligonucleotides		
*EpBmall* HCR™ probe (v3.0)	Molecular Instruments, Inc., this paper	LOT: PRP939
*EpBmall* additional HCR™ probe (v3.0)	Molecular Instruments, Inc.	LOT: RTA402
*EpCIk 1-9* HCR™ probe (v3.0)	Molecular Instruments, Inc., this paper	LOT: RTA400
*EpClk 1-9* additional HCR™ probe (v3.0)	Molecular Instruments, Inc., this paper	LOT: RTE285
*Epcry2* HCR™ probe (v3.0)	Molecular Instruments, Inc., this paper	LOT: RTA399
*Epcry2* HCR™ probe (v3.0)	Molecular Instruments, Inc., this paper	LOT: RTO453
*Epper* HCR™ probe (v3.0)	Molecular Instruments, Inc., this paper	LOT: RTO454
*Eptim* HCR™ probe (v3.0)	Molecular Instruments, Inc., this paper	LOT: PRP937
*PhBmal1* HCR™ probe (v3.0)	Molecular Instruments, Inc., this paper	LOT: RTC080
*Phper* HCR™ probe (v3.0)	Molecular Instruments, Inc., this paper	LOT: RTK407; LOT: RTC082
*PhClk*-fragment 1 HCR™ probe (v3.0)	Molecular Instruments, Inc., this paper	LOT: RTG497
*PhClk*-fragment 2 HCR™ probe (v3.0)	Molecular Instruments, Inc., this paper	LOT: RTG498
*Phcry2* HCR™ probe (v3.0)	Molecular Instruments, Inc., this paper	LOT: RTC083
*deGFP* HCR™ probe (v3.0)	Molecular Instruments, Inc.	LOT: RTA210
*deGFP* HCR™ probe (v3.0)	Molecular Instruments, Inc.	LOT: PRQ740
*Epper-C4* RNAScope™ probe	ACD Biotechne, this paper	Cat#578211-C4
*EpClk5-C1* RNAScope™ probe	ACD Biotechne, this paper	Cat#578151-C1
*Epcry2-C1* RNAScope™ probe	ACD Biotechne, this paper	Cat#1039411-C1
*Eptim-C3* RNAScope™ probe	ACD Biotechne, this paper	Cat#1099401-C3
*EpBmall*-C2 RNAScope™ probe	ACD Biotechne, this paper	Cat#578191-C2
-ve control RNAScope™ probe	ACD Biotechne	N/A
Software and algorithms		
FIJI ImageJ	Schneider et al., (2012) Nat Methods^28^	https://imagej.net/software/fiji/
Computational Morphometry Toolkit (CMTK)		https://www.nitrc.org/projects/cmtk
MakeAverageBrain		https://github.com/jefferislab/MakeAverageBrain
3DSlicer	Kikinis et al. (2014)^29^	https://www.slicer.org/
DAMSystem3 software	TriKinetics Inc.	N/A
R Studio	RStudio Team (2020)^30^	http://www.rstudio.com/
Rethomics	Geissmann et al.^28^	https://rethomics.github.io/
Cellpose 2.0	Pachitariu and Stringer^32^	https://www.cellpose.org/
napari	napari contributors	https://napari.org/
Fusion software	Andor, Oxford Instruments	N/A
RS-FISH ImageJ plugin	Bahry et al.^33^	https://github.com/PreibischLab/RS-FISH
Graphpad Prism		N/A
Other		
DAM10, LAM10, LAM25 activity monitors	Trikinetics Inc.	N/A
double-sided adhesive tape	Radio Spares	Cat#770-3422

### Experimental Model and Study Participant Details

#### *Eurydice pulchra* collection

Adult males and females were collected using a hand-towed net from Llanddona beach, Anglesey, UK, during spring high tides from May to October.^12^

#### *Parhyale hawaiensis* husbandry

Male and female Chicago F strain *P. hawaiensis* were maintained in 6.5 L polypropylene containers (h: 125 mm, w: 260 mm, d: 260 mm) containing 30–35 psu ASW and a layer of crushed coral substrate (grain size 2–20 mm). Animals were reared at 25°C under 12h light:dark (LD) cycles. The containers were covered with clear Perspex lids to prevent evaporation and aerated using aquarium air pumps and air stones. Animals were fed carrots, goldfish flakes (Tetra GmbH, Germany), algae wafers (Kyorin Food Ind. Ltd., Japan), freeze-dried brine shrimps, and/or freeze-dried Tubifex (Interpret, UK) once weekly. Full water changes were performed fortnightly.

### Method Details

#### *Eurydice pulchra* behavior analysis

Swimming behaviour of adult females and males was recorded in DAM10 and LAM10 activity monitors (Trikinetics Inc., USA) under DD at 16°C with animals housed individually in 3 ml (Ø: 11 mm) plastic vials containing 33 psu artificial seawater (ASW, Reef Salt, Tropical Marine Centre, Germany) and ∼5 mm depth of fine sand. Infrared beam interruptions were recorded using the DAMSystem3 software (TriKinetics Inc., USA) in one-minute bins. The first peak in tidal swimming activity was omitted from analysis and behavioural rhythmicity was assessed over the subsequent six tidal cycles using chi-squared periodogram analysis in Rethomics R package.^31^

#### *Parhyale hawaiensis* behavior analysis

For locomotor activity monitoring, adult female and male animals were placed individually into 23 mL polystyrene vials (h: 75 mm, Ø: 23.5 mm) containing a layer (∼2 mm) of crushed coral substrate (grain size 2–5 mm) and approximately 6 cm of 30 psu ASW at 25°C. Tubes were loosely covered with parafilm and loaded into two-tier activity monitors modified from the LAM25 Locomotor Activity Monitors (TriKinetics Inc., USA) configured to record swimming and/or roaming activity. Infrared beam interruptions were recorded using the DAMSystem3 software (TriKinetics Inc., USA).

Beam interruptions were collected into 30-minute bins and normalised within each animal by dividing the count in each bin by the average count across all bins for that animal. Autocorrelation periodogram was used to determine whether animals were rhythmic, with a significance threshold of *α* = 0.05.^34^ The period and the power of rhythmicity were determined using Lomb-Scargle periodogram analysis^35^ of binned and normalised data at the significance level *α* =0.05. For circatidal experiments, the first subjective high tide after loading was omitted from analysis and behavioural rhythmicity was assessed over the subsequent six tidal cycles. For circadian entrainment experiments, animals loaded into the monitor were subjected to at least four 12:12 LD (ZT0/lights-on at 0600 local time) cycles before being allowed to free run in DD. For tidal entrainment, animals were kept under LD as above but mechanically agitated on an orbital shaker set at 50–60 rpm for 2 hours at 12.4-hour intervals. For behavioural monitoring, animals were taken from entrainment regimes during the agitation phase and placed in the activity monitor as described above to free-run without agitation in DD.

### Brain collection and processing

Animals were anaesthetised in ice-cold seawater, and their brains dissected using sharpened watch-maker’s forceps in ice-chilled physiological saline^36^ (*E. pulchra*) or 0.22 μm filter-sterilised ASW (30 -35 psu, FASW; *P. hawaiensis*). Tissues were immediately fixed in 4% paraformaldehyde (made in PBS for *E. pulchra* and FASW for *P. hawaiensis*) for 1 hour at room temperature, dehydrated serially in 33%, 66%, 100% methanol in PBST (PBS supplemented with 0.1% Tween20), and stored in 100% methanol at -20°C for at least 16 h before hybridisation or immunohistochemistry. Immediately prior to use, brains were rehydrated in 66/33% methanol and washed/equilibrated in PBST (3×10 min).

### Immunohistochemistry

Neuropils were immunolabelled using anti-SYNORF1 antibody (3C11, Developmental Studies Hybridoma Bank (DSHB)) following.^37,38^ 3c11 (anti SYNORF1) is a mouse monoclonal anti-body against *Drosophila* synapsin and with confirmed reactivity against crustaceans (as well as insects, cephalopods, leech and planaria). For *E. pulchra*, after rehydration to PBST, brains were washed in PTx (PBS supplemented with 0.1% Triton X-100), permeabilised in detergent solution (50 mM Tris-HCl pH 7.5, 150 mM NaCl, 1 mM EDTA pH 8, 1% SDS, 0.5% Tween-20) for 30 min at room temperature^39^ and then incubated with 1:50 anti-SYN-ORF1 in PTx containing 5% normal goat serum (NGS) overnight at room temperature, followed by 5 days at 4°C, with rotation. After extensive washing in PTx, brains were incubated with anti-mouse Alexa 488 (ThermoFisher #A-21141) at 1:1000 in PTx containing 5% NGS for 3 days at 4°C, with rotation. After extensive washing with PTx, nuclei were stained with 0.1 μg/mL DAPI int PTx for 1h. Brains were then bridge-mounted in RapiClear 1.49 (SunJin Lab Optical Clearing Innovation, #RC149001) using No. 2 thickness coverslips to maintain 3D morphology. For *P. hawaiensis*, brains were incubated in blocking buffer (1x PBS, 0.5% Tween-20, 5% NGS, 1% bovine serum albumin) containing 1:50 anti-SYNORF1 for 3 days at 4°C with gentle shaking, followed by extensive washing in PTw (PBS supplemented with 0.5% Tween-20) and incubation in secondary antiserum F(ab^′^)2-goat anti-mouse IgG-Alexa Fluor 488 (ThermoFisher #A-11017) or Alexa Fluor Plus 555 (ThermoFisher #A48287) at 1:250 made in blocking buffer for 2 days at 4°C with gentle agitation. Nuclei were stained with 0.1 μg/mL DAPI for 30 min prior to final washing in PTw and preparations mounted in DPX (Sigma Aldrich #06522) according to.^38^

### Reference brains

Whole-mount, immunolabelled (see above) brain samples used for generating reference brains and describing the neuroanatomy of putative clock cells were imaged on a Nikon CSU-W1 spinning disk confocal microscope equipped with a 25x/1.05NA silicone oil CFI Plan Apochromat Lambda S objective and a Prime 95B 22mm sCMOS camera. To make an *E. pulchra* reference brain, two sex-specific references were made by registering and then averaging 7 male brains to one male “seed” brain and 6 female brains to a female “seed” brain. These sex-specific references were then averaged to generate a final intersex reference brain (an average of 15 brains) used for all subsequent analyses. For *P. hawaiensis*, 10 male brains were registered and then averaged to a seed brain to generate a reference brain. Registration and averaging were done using: https://github.com/jefferislab/MakeAverageBrain.^40,41^ Briefly, Computational Morphometry Toolkit (CMTK) was used to register image stacks against a seed image stack; first with a rigid and then a non-rigid registration.^42,43^ Image stacks of brains with co-immuno-RNA-FISH labelling of neuropils and circadian clock genes (*per*/ *cry2*/ *tim*) were registered to reference brains based on the anti-SYNORF1 signal. Three-dimensional surface models of neuropils were based on reference brains and generated using the Segmentation Editor in 3DSlicer software (www.slicer.org). 3D surface models of putative clock cells were based on circadian clock gene HCR-FISH labelling (*Epcry2* or *Phcry2*) in an image stack registered to the reference brain and using the Segmentation Editor in 3DSlicer.

### Hcr™ RNA-Fish

HCR™ probes (v3.0) were designed and synthesised by Molecular Instruments Inc, USA against user-supplied sequences (Table S8). HCR-FISH followed the manufacturer’s protocol^44^ with some minor alterations following.^39^ For HCR-FISH, rehydrated brains were permeabilised in detergent solution for 30 min at room temperature, and equilibrated in pre-warmed probe hybridisation buffer for 30 min at 37°C. Brains were then incubated in probe hybridisation solution containing 10 nM of each primary probe set for 2 days (*E. pulchra*) or 3 days (*P. hawaiensis*) at 37°C. For *P. hawaiensis*, 1:1000 murine RNase inhibitor (NEB #M0314L) was added to the probe hybridisation buffer. Signal amplification with 60 nM fluorescently tagged hairpins was allowed to proceed for 2 days, followed by staining with 0.1 μg/mL DAPI in 5x SSC supplemented with 0.5% Tween-20 (30 min) during one of the final washing steps. *E. pulchra* brains were bridge-mounted using No. 1.5 thickness coverslips in RapiClear 1.49 whilst *P. hawaiensis* brains were dehydrated and mounted in DPX according to.^38^ The target genes used for probe design, their accession numbers, and the Molecular Instruments probe lot numbers and concentrations used are listed in Table S8. For experiments investigating co-expression of clock genes, a 40x/1.3NA Plan Fluorite oil immersion objective was used to image with an optical section interval of 1 μm.

### Co-immuno-RNA-FISH

To co-localise putative clock cells relative to major neuropils, immunostaining with anti-SYNORF1 antibody was followed by HCR-FISH. Reference brains were generated from confocal image stacks of brains in which neuropils were labelled with anti-SYNORF1 (see above). For co-immuno-RNA-FISH staining, following rehydration and permeabilization in detergent solution, *E. pulchra*, brains were incubated with 1:50 anti-SYNORF1 in PTx containing 1.6 U/μL RNaseOUT™ ribonuclease inhibitor (Invitrogen #10777019) over-night at room temperature, followed by 3 days at 4°C, with rotation. After extensive washing in PTx, brains were incubated with anti-mouse Alexa 488 (ThermoFisher #A-21141) at 1:1000 in PTx containing RNase inhibitor for 2 days at 4°C, with rotation. After washing in PTx/w, brains were fixed in 4% PFA (in PBS) for 1 hour at room temperature. After washing in PTx/w, the neuropil-stained brains were processed for HCR-FISH as above and mounted as described above. *P. hawaiensis* brains were washed in PTw and subjected to one-step immunostaining^45^ by incubating in PTw containing pre-mixed 1:250 murine RNase inhibitor, 1:50 anti-SYNORF1, 3.33 ng/μL Fab goat anti-mouse IgG-Alexa Fluor 488 (Jackson ImmunoResearch #115-547-003) for 2 days at room temperature.

### RNAscope methods for formalin-fixed, paraffin-wax embedded *E.pulchra* brains

*E. pulchra* brains were dissected in ice-chilled crustacean saline^36^ and fixed immediately in 10% normal buffered formalin (10%) overnight (16h) at RT. After fixing, brains were washed extensively in PBS, dehydrated through an ethanol series (50%, 70%, 80%, 90%, 95%, 100%), cleared in xylene (1h at RT) and infiltrated in molten wax (Leica Paraplast Plus®, Leica Biosystems, Sheffield UK) 3x, each for 1h, before embedding in wax such that the brains could be mounted for frontal sections to be cut. Serial sections (6μm) were cut on a standard wax microtome and mounted on Superfrost™ Plus (Thermo Fisher, UK). After drying, sections were baked at 60°C for 2h to adhere fully to the slides. Slides were de-waxed in clean xylene and taken to water through an ethanol series before drying at 60C for 1h. Following preparation, manufacturer’s instructions were followed exactly, except that target retrieval was done in 700 mL Target Retrieval buffer in a 1L beaker, for 15 minutes at 98-104°C, over a hot-plate. Protease Plus was applied for 30 minutes at 40°C. Signal amplification was done using Opal™ fluors (Opal 520 and Opal 570). All reagents and kit components were as recommended by ACD Biotechne (Abingdon, Oxford, UK. RNAscope® Multiplex Fluorescent Reagent Kit v2 Assay). Target probes were designed by ACD Biotechne and probe details provided in Table S9. Negative control probes were supplied by ACD Biotechne and are designed against the *DapB2* gene of *Bacillus subtilis*. Following hybridisation and amplification, sections were counterstained with DAPI provided with the kit and mounted under Vectashield® Antifade mounting fluid (Vector Laboratories, supplied by 2B Scientific, Oxford, UK). Images were captured on a Leica SP8 confocal microscope at 1μm z-stack intervals and using Leica LAX proprietary software. Images were re-sized, cropped and adjusted for brightness and contrast using ImageJ software.

### Time-course sampling design

To investigate clock gene expression pattern in the brain of *E. pulchra*, animals were collected from Llanddona beach, Anglesey, UK, during spring high tides in October 2024 and loaded into activity monitors. Prior to sampling, animals were maintained under 18 h: 6 h L:D cycle for 24 hours. Animals showing peaks of swimming activity during the two subjective high waters during this period were sampled every two hours, beginning at ZT7. To investigate daily clock gene expression pattern in the brain of *P. hawaiensis*, animals were loaded into activity monitors and subjected to 12 h: 12 h LD cycle for 6 days as described. Individuals showing significant 24-h rhythmicity in locomotor activity (as described) over this entrainment period up to the day before sampling were marked for sampling. On the day of sampling, marked animals were sampled every 2 hours, beginning at ZT1 and ending at ZT25, for a total of 13 timepoints.

To investigate circatidal clock gene expression pattern in the brain of tidally entrained *P. hawaiensis*, animals which have been subjected to tidal entrainment were loaded into activity monitors as described. Sampling occurred every 3 hours, beginning at CT0 on the day after monitor loading and ending at CT24, for a total of 9 timepoints. Individuals were only sampled if they showed peaks of locomotor activity during the subjective high tides preceding their sampling timepoint.

Dissected brains were processed for digital HCR-FISH as described. HCR-FISH probes used for time-course experiments are described in Table S10.

### Whole brain digital HCR-FISH

For quantification of diffraction-limited spots,^44^ signal amplification with 60 nM of each hairpin was performed for 1 h, followed by staining with 1 μg/mL DAPI (as above). Brains were mounted in Prolong Glass Antifade Mountant (ThermoFisher #P36980). *E. pulchra* brains were mounted with the posterior surface apposed to the coverslip whilst *P. hawaiensis* brains were mounted with the anterior surface apposed. To minimise signal loss with imaging depth, brains were compressed to within 40 μm by mounting the coverslips containing brains onto spacers made from double-sided adhesive tape (Radio Spares #770-3422). An Andor BC43 CF benchtop microscope equipped with a 60x/1.42NA Plan Apochromat oil immersion objective was used to image each brain with an optical section interval of 0.3 μm.

To prepare images for segmentation of cell groups enriched for expression of *per, cry2*, and/ or *Clk/ tim*, 2D maximum filters were applied to each HCR-FISH channel, followed by summation of the filtered images across channels and 2D gaussian filtering. The resulting images, together with the corresponding raw DAPI image as the auxiliary channel, were subjected to segmentation using Cellpose^46^ and a custom model (available at GitHub) trained via the human-in-the-loop functionality of the Cellpose 2.0 graphical user interface.^32^ The resulting masks were manually curated as required in napari^47^ and assigned to distinct cell groups. For spot detection, images were deconvolved using ClearView™ Deconvolution with 10 iterations after being acquired in the Fusion software. RNA foci were then detected in deconvolved images and assigned to cell groups using the RS-FISH ImageJ plugin.^33^

### Quantification and Statistical Analysis

Statistical details of test performed are reported in figure legends and Tables S2–S7. Graphs present data as mean ±SEM (unless otherwise stated). Chi-squared (*E. pulchra*) and Lomb-Scargle (*P. hawaiensis*) periodogram analysis were used to determine power and period of locomotor activity. For quantification of transcript abundance by FISH spot counts, Shapiro-Wilk test was used to determine normal distribution of data and Levene’s test was used to determine if the variances of all groups are equal. An overall time effect was tested by ANOVA for normally distributed data and by Kruskal-Wallis for non-normal datasets. Where this yielded a statistically significant effect, rhythmicity in the data was analysed further by JTK CYCLE^20^ and RAIN^21^ at 12-hour or 24-hour periods. JTK-cycle p-values are adjusted using Bonferroni’s method, and RAIN p-values are adjusted using the adaptive Benjamini-Hochberg (default) method. Gene expression was considered rhythmic when ANOVA was significant, and both JTK CYCLE and RAIN were significant for the same period (12 hours or 24 hours).

## Supplementary Material

Document S1.

Document S2.

Supplemental information

## Data Availability

All original code has been deposited at https://github.com/HastingsLab-LMB/fish_pipeline and is publicly available at https://figshare.com/projects/2025_crustacean_clock_cell_rhythms/240869 as of the date of publication. HCR-FISH and behavioral data reported in this paper will be shared by the lead contact upon request. Any additional information required to reanalyze the data reported in this paper is available from the lead contact upon request.

## Highlights

- Circadian clock gene expression mapped in brains of two tidally rhythmic crustaceans
- Discrete populations of ∼60 putative clock cells identified in both species
- Separate cells in same brain exhibit either daily or tidal clock gene expression
- Canonical circadian clock genes implicated in circatidal clockwork

## In brief

Oliphant and Sia et al. explore the basis of tidal clocks in crustaceans by mapping canonical circadian clock gene expression across *Eurydice* and *Parhyale* brains, revealing comparable clusters of ∼60 putative clock cells. Daily and tidal expression rhythms in different cells in the same brains implicate circadian clock genes in circatidal clockwork.
