# Supplementary material for: Expression of clock genes tracks daily and tidal time in brains of intertidal crustaceans Eurydice pulchra and Parhyale hawaiensis
Source: Curr Biol. Author manuscript; Available in PMC 2025 Jul 30. (PMC7617966; doi:10.1016/j.cub.2025.04.047)
Supplement: Document S1. [file EMS207343-supplement-Document_S1_.pdf]

Current Biology, Volume *35*

Supplemental Information

Expression of clock genes tracks daily and tidal time in brains of intertidal crustaceans *Eurydice pulchra* and *Parhyale hawaiensis*

Andrew Oliphant, Chee Y. Sia, Charalambos P. Kyriacou, David C. Wilcockson, and Michael H. Hastings

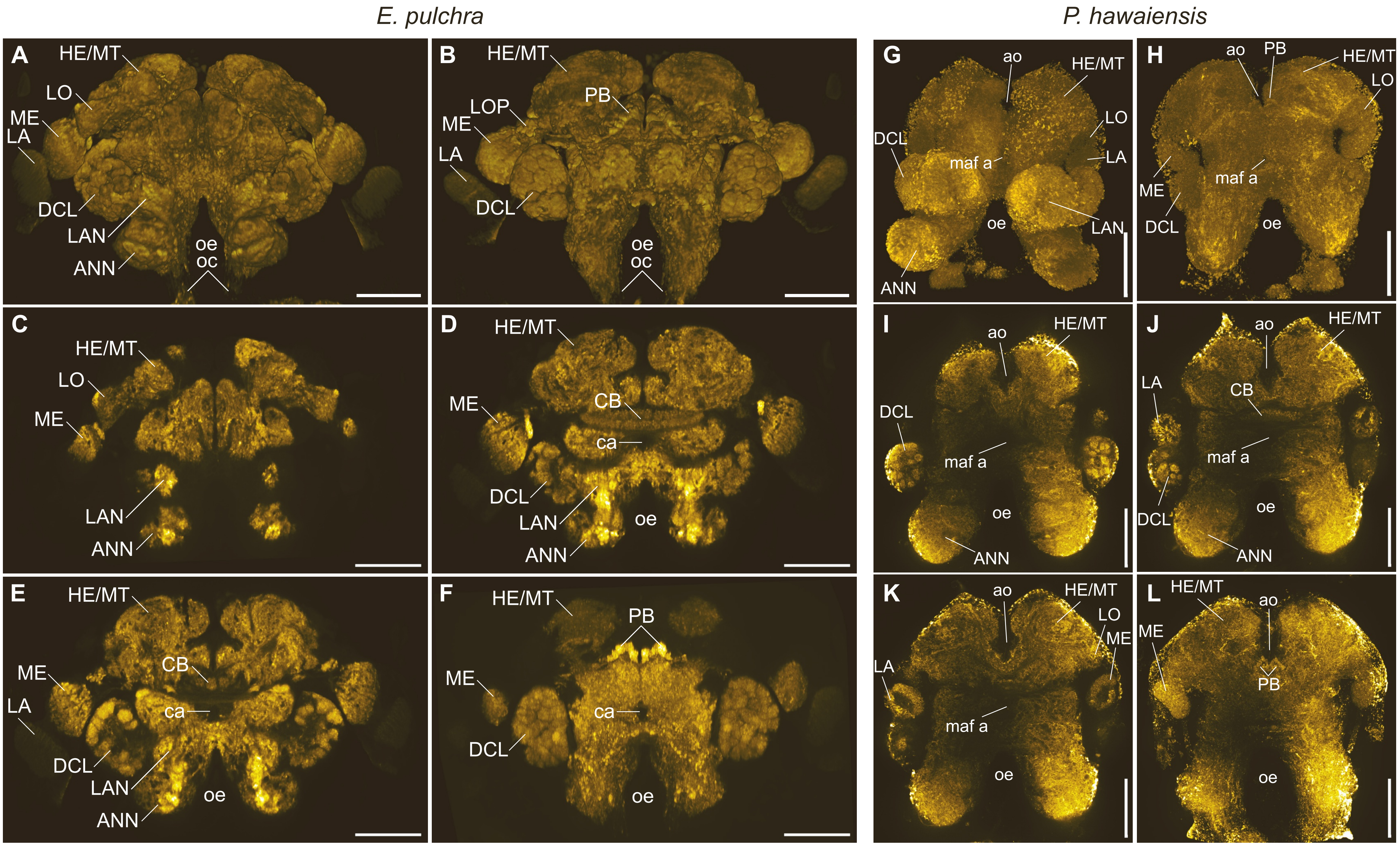
E. pulchra
P. hawaiensis
A
HE/MT
LO
ME
LA
DCL
LAN
ANN
oe
oc
B
PB
LOP
C
D
CB
ca
E
F
G
ao
maf a
H
I
J
K
L

Figure S1. Overview of representative *E. pulchra* and *P. hawaiensis* brains, immunostained with anti-SYNORF1 to highlight the neuropils. Related to Figures 1 and 2.

(A) Anterior view of a *E. pulchra* brain (3D volume projection), with optic lobes, deutocerebral chemosensory lobes, antenna neuropils and hemi-ellipsoid body/medulla terminalis complexes visible on the anterior surface of the brain.

(B) Posterior view of a *E. pulchra* brain (3D volume projection). The optic lobes, deutocerebral chemosensory lobes and hemi-ellipsoid body/medulla terminalis complexes are visible on the posterior surface. The protocerebral bridge appears as a distinct arch-like structure under the hemi-ellipsoid body/medulla terminalis complexes.

(C- E) Selected optical sections of a *E. pulchra* brain from anterior to posterior, showing locations of distinct neuropils throughout the depth of the brain. The optic lobes span the anterior-posterior axis, with the lobula constrained to the anterior aspect (C), and the medulla and lamina (D, E) occupying the medial and posterior aspects. The cigar-shaped central body is at its most expansive towards the middle of the brain.

(F) Maximum intensity Z-projection through the protocerebral bridge of a *E. pulchra* brain.

(G) Anterior view of a *P. hawaiensis* brain (3D volume projection), with parts of the optic lobes (lamina and lobula), deutocerebral chemosensory lobes, antenna neuropils and hemi-ellipsoid body/medulla terminalis complexes visible on the anterior surface of the brain.

(H) Posterior view of a *P. hawaiensis* brain (3D volume projection). Parts of the optic lobes (medulla and lobula), deutocerebral chemosensory lobes and hemi-ellipsoid body/medulla terminalis complexes are visible on the posterior surface. As in *E. pulchra,* the protocerebral bridge of *P. hawaiensis* is located posteriorly under the hemi-ellipsoid body/medulla terminalis complexes.

(I- K) Selected optical sections of a *P. hawaiensis* brain from anterior to posterior, showing locations of distinct neuropils throughout the depth of the brain. The hemi-ellipsoid body/medulla terminalis complexes and the foramina accommodating two of the brain arteries, the anterior aorta and myoarterial formation a, are visible throughout the thickness of the brain. The former also constitute the most dorsal neuropils of the brain. The deutocerebral chemosensory lobes are located anterior to the rest of the optic lobes (I, J). The lamina is the most anteriorly positioned optic neuropils (J), followed almost simultaneously by the medulla and lobula (K). The sausage-shaped central body is at its most expansive towards the middle of the brain (J).

(I) Maximum intensity Z-projection through the protocerebral bridge of a *P. hawaiensis* brain.

Abbreviations: ANN, antenna 2 neuropil; ao, anterior aorta; ca, cerebral artery; CB, central body; DCL, deutocerebral chemosensory lobe; HE/MT, hemi-ellipsoid body/medulla terminalis; LA, lamina; LAN, lateral antenna 1 neuropil; LO, lobula; LOP, lobula plate; maf a,

myoarterial formation a; ME, medulla; oc, oesophageal connective; oe, oesophageal foramen; PB, protocerebral bridge. Scale bars*:* 100 μm.

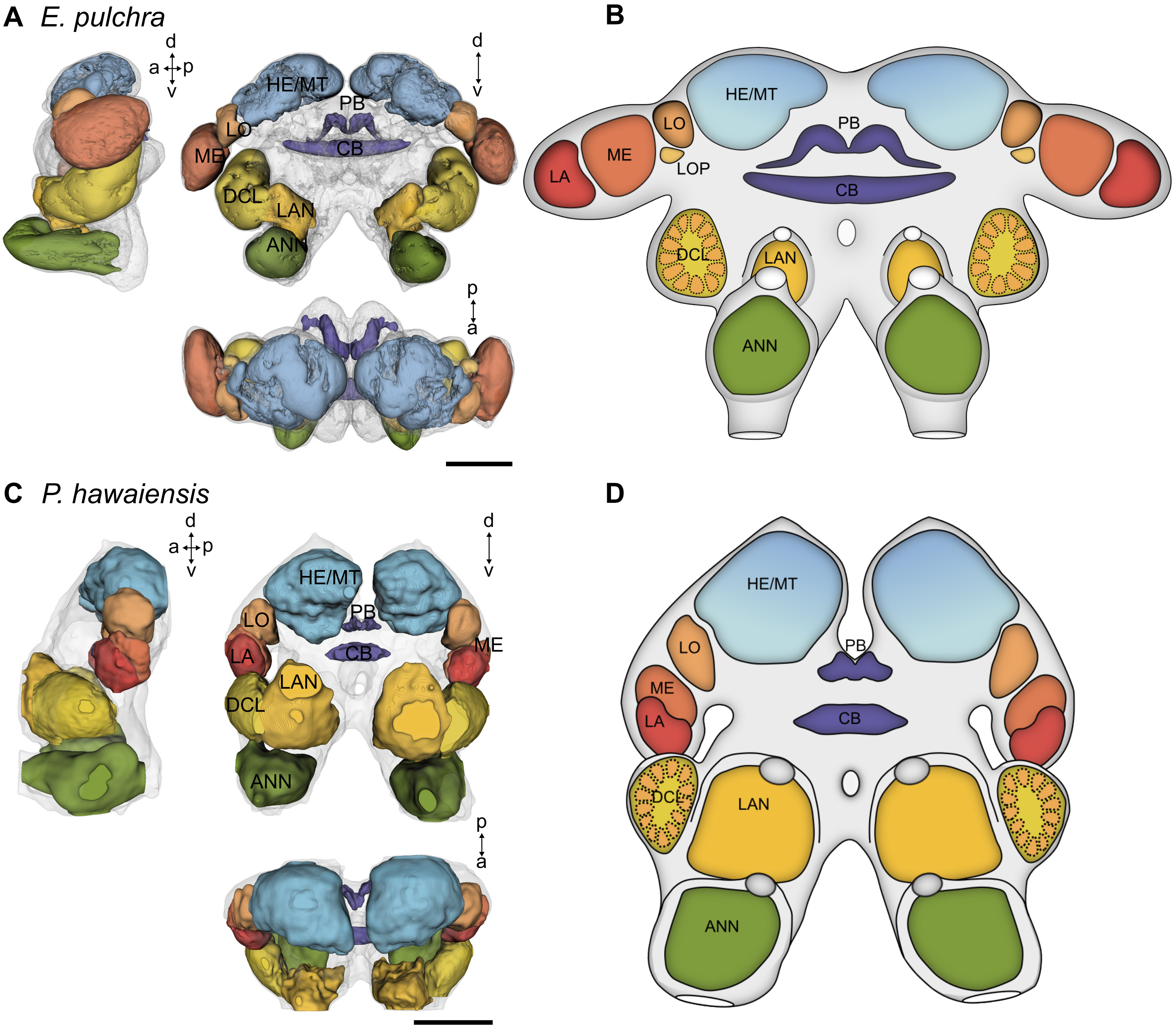

A E. pulchra
B
C P. hawaiensis
D
d
a
p
v
HE/MT
PB
LO
CB
ME
LA
LOP
DCL
LAN
ANN

Figure S2. Gross anatomy of *E. pulchra* and *P. hawaiensis* brains revealed by anti-SYNORF1 immunostaining, registered to reference brain. Related to Figures 1 and 2.

(A) Lateral, anterior and dorsal views of the *E. pulchra* surface-rendered 3D average reference brain, with major neuropils demarcated. See also Video S1.

(B) Diagrammatic representation of the of *E. pulchra* brain, based on A.

(C) Lateral, anterior and dorsal views of *P. hawaiensis* surface-rendered 3D average reference brain with major neuropils demarcated. See also Video S2.

(D) Diagrammatic representation of the *P. hawaiensis* brain, based on C.

Compass markers in A and C show anterior (a), posterior (p), dorsal (d) and ventral (v) directions.

Abbreviations: ANN antenna 2 neuropil, CB central body, DCL deutocerebral chemosensory lobe, HE/MT medulla terminalis/ hemi-ellipsoid body, LA lamina, LAL lateral accessory lobe, LAN lateral antenna 1 neuropil, LO lobula, LOP lobula plate, ME medulla, PB protocerebral bridge. Scale bars: 100 µm.

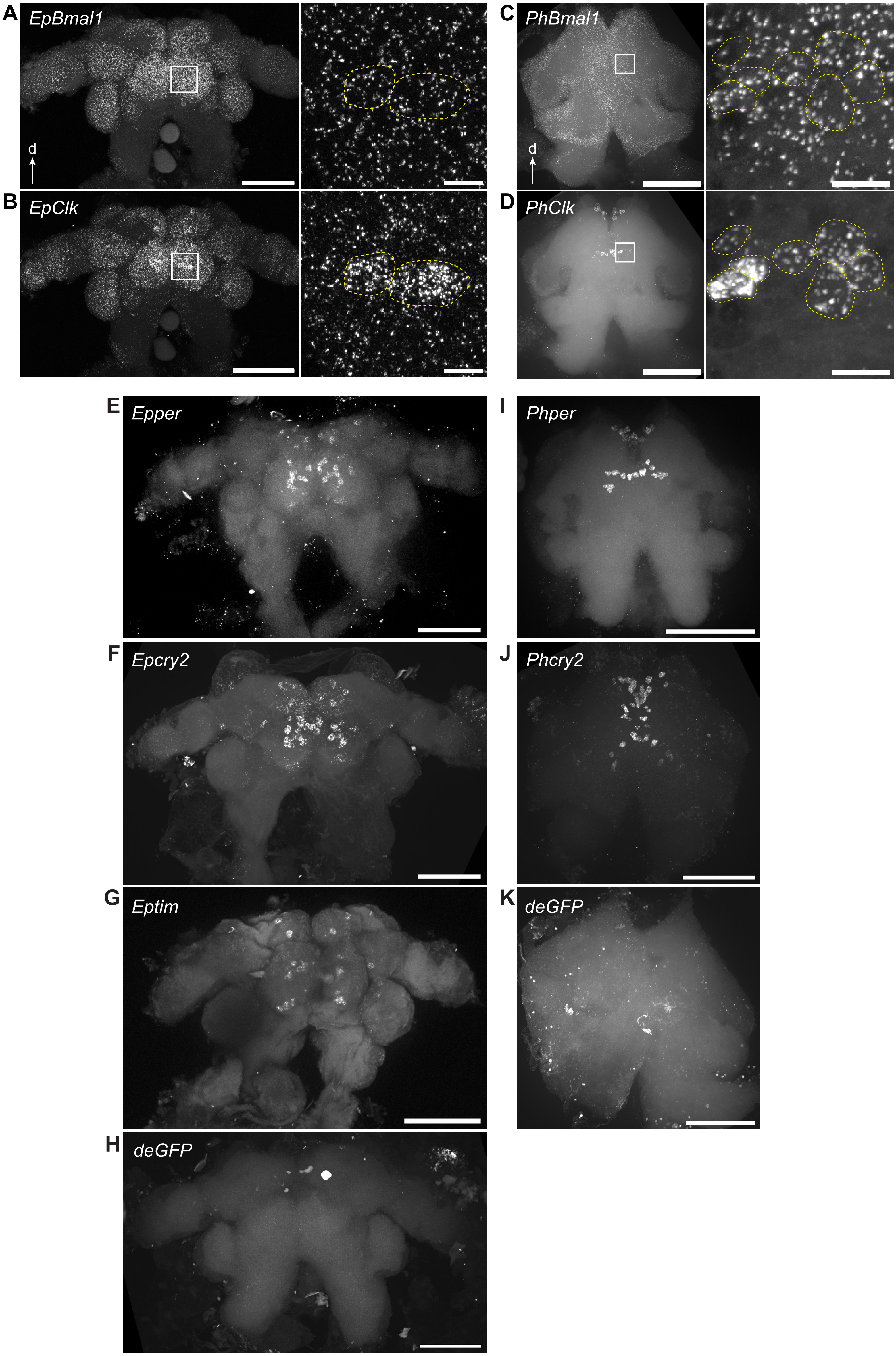

A
EpBmal1
d
B
EpClk
C
PhBmal1
d
D
PhClk
E
Epper
F
Epcry2
G
Eptim
H
deGFP
I
Phper
J
Phcry2
K
deGFP

Figure S3. Expression of circadian clock positive and negative regulators across the brains of *E. pulchra* and *P. hawaiensis*. Related to Figures 1 and 2.

(A) Left panel: maximum intensity Z-projection of image stack of an entire *E. pulchra* brain (posterior apposing coverslip) probed for circadian factor *EpBmal1* using HCR-FISH. Right panel: maximum intensity Z-projection of two-cell-thick image stack (16 μm) from the boxed region of the brain in the left panel.

(B) As in A, probed for circadian factor, *EpClk*.

(C) Left panel: maximum intensity Z-projection of image stacks of a *P. hawaiensis* brain (anterior apposing coverslip) probed for circadian factor *PhBmal1*. Right panel: maximum intensity Z-projection of two-cell-thick image stacks (24 μm) from the boxed region of the brain in the left panels.

(D) As in C, probed for circadian factor, *PhClk*.

(E, F, G, H) Maximum intensity Z-projections of image stacks of *E. pulchra* brains (posterior apposing coverslip) probed for circadian clock repressors, *Epcry2, Epper*, *Eptim* and the negative control probe *deGFP* HCR-FISH.

(I, J, K) Maximum intensity Z-projections of image stacks of *P. hawaiensis* brains (anterior apposing coverslip) probed for circadian clock repressors, *Phcry2*, *Phper* and the negative control probe *deGFP*. Scale bars: 100 μm.

Arrows indicate dorsal orientation. Yellow dotted outlines in A- D indicate cells in which *Clk* expression is enriched. Scale bars: 100 μm (A-D: left panels, E-K) and 10 μm (A-D: right panels).

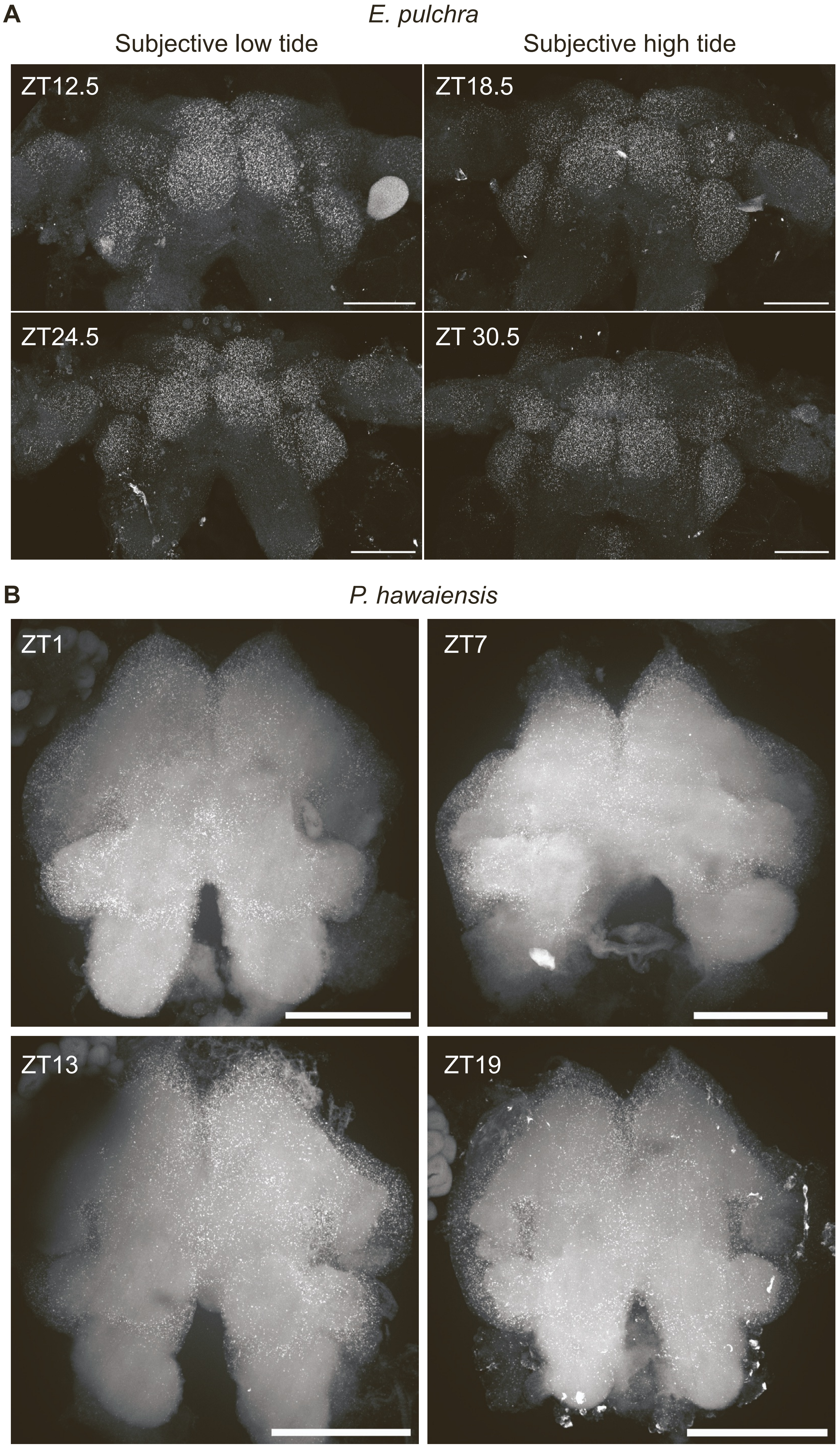
A
E. pulchra
Subjective low tide
Subjective high tide
ZT12.5
ZT18.5
ZT24.5
ZT 30.5
B
P. hawaiensis
ZT1
ZT7
ZT13
ZT19

Figure S4. Cellular expression of *Bmal1* in brains of *E. pulchra* and *P. hawaiensis* across time. Related to Figures 1 and 2.

(A) Maximum intensity Z-projections of representative brains (posterior apposing coverslip) from field-collected *E. pulchra* sampled under 14 h:10 h LD cycle and then probed for *EpBmal1* using HCR-FISH.

(B) Maximum intensity Z-projections of representative *P. hawaiensis* brains (anterior apposing coverslip) sampled under 12 h: 12 h LD cycle and then probed for *PhBmal1* using HCR-FISH. Scale bars: 100 μm.

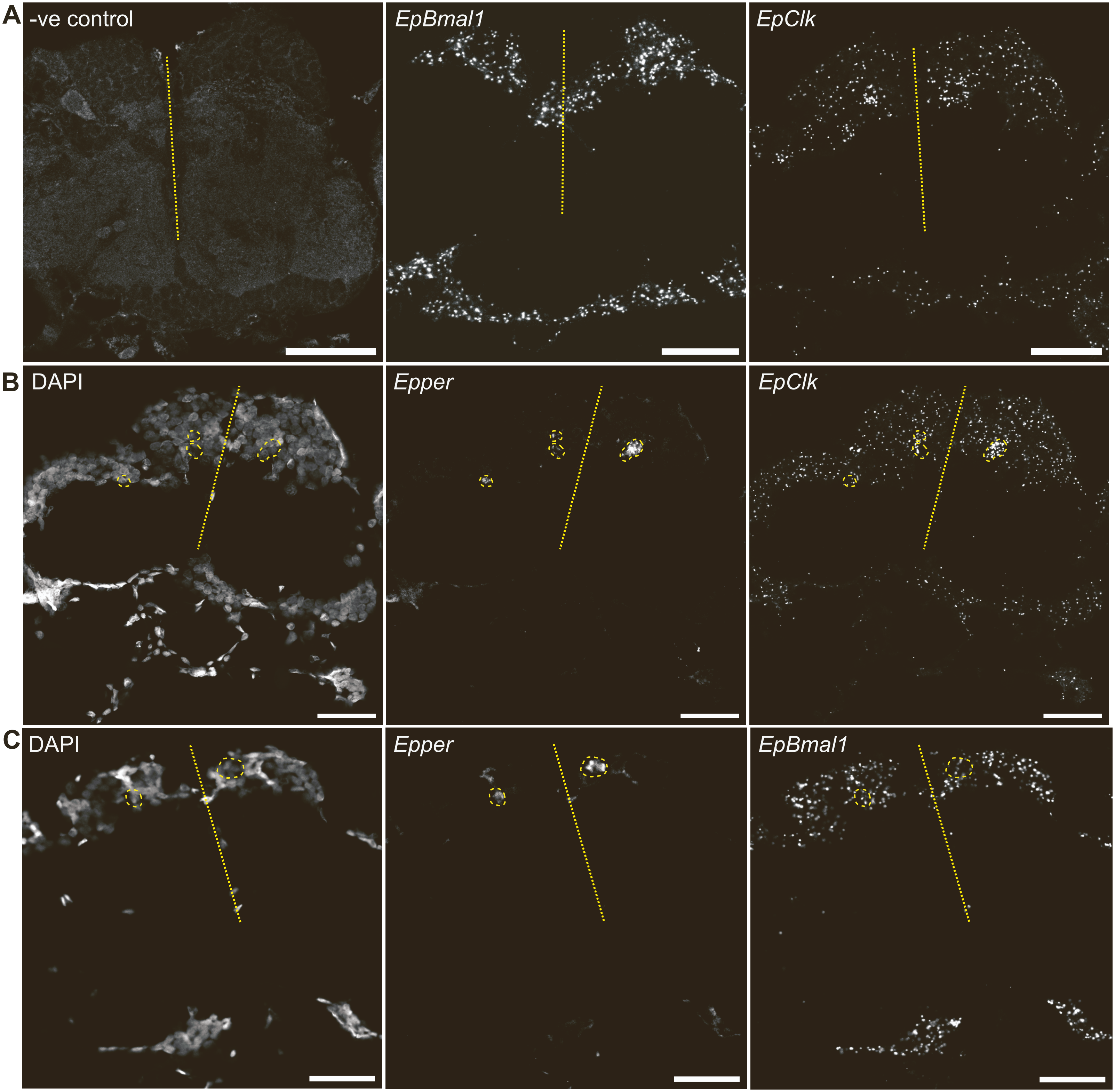
A
-ve control
EpBmal1
EpClk
B
DAPI
Epper
EpClk
C
DAPI
Epper
EpBmal1

Figure S5. Expression of circadian clock genes in *E. pulchra* brain sections detected using RNAScope™. Related to Figures 1 and 2.

(A) Representative image (single optical section) of *E. pulchra* brain sections probed for negative control (left), *EpBmal1* (middle) and *EpClk* (right).

(B) Representative image (single optical section) of a *E. pulchra* brain section stained with DAPI (left) and probed for *Epper* (middle) and *EpClk* (right).

(C) As in B for *Epper* and *EpBmal1*.

Yellow dashed outlines indicate cells enriched for *Epper* and *EpClk* expression. Yellow dotted lines indicate midlines. Scale bars: 10 μm.

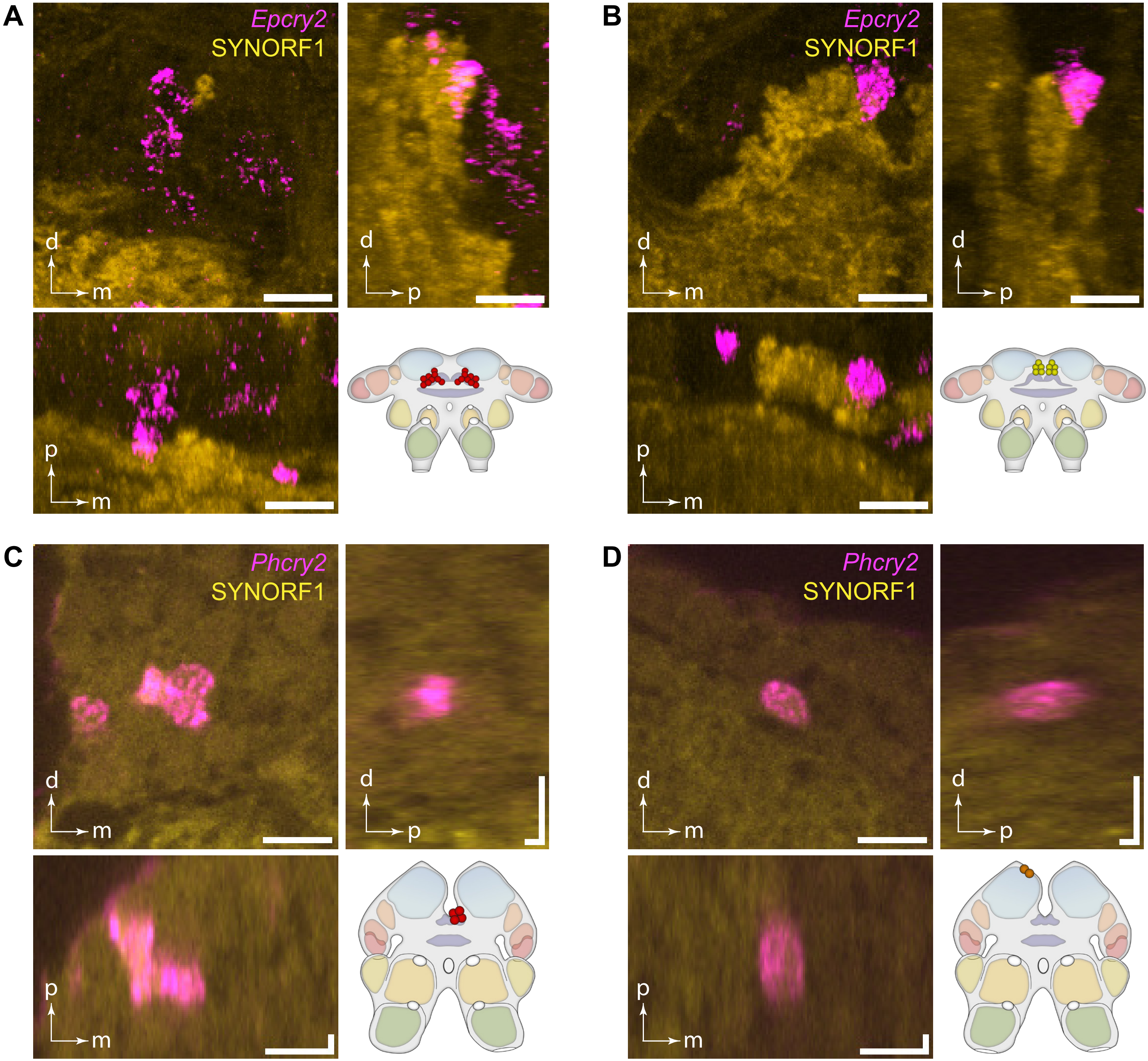
A
Epcry2
SYNORF1
d
m
p
B
Epcry2
SYNORF1
C
Phcry2
SYNORF1
D
Phcry2
SYNORF1

Figure S6. Representative orthogonal views of brains co-labelled with anti-SYNORF1 (IHC) and *cry2* (HCR-FISH). Related to Figures 1 and 2.

(A) Orthogonal views showing *Epcry2* enrichment in four medioposterior cells of a *E. pulchra* brain co-stained with anti-SYNORF1. Cartoon shows location of these cells.

(B) As in A but for the medial cells.

(C) Orthogonal views showing *Phcry2* enrichment in four medioposterior cells of a *P. hawaiensis* brain co-stained with anti-SYNORF1. Cartoon shows location of these cells.

(D) As in C but for the two dorsal-lateral cells.

Horizonal (and vertical) scale bars: 10 µm. Orientations: d: dorsal; p: posterior; m: medial.

In *E. pulchra, per-* and *cry2*-expressing cells were primarily found in the posterior cell body rind overlying the medial protocerebrum (MP), hemi-ellipsoid body and medulla terminalis (HE/MT, Figure 2A, B). There were nine somata in the rind posterior to the protocerebral bridge (rPBp) [S 1], which we termed the "medioposterior" cells. Dorsal and anterior to these cells was a cluster of 5 somata in the cell body rind dorsal to the PB (rPBd) that we assigned as "medial" cells. Lateral to these medial and medioposterior cells was a cell pair in the rind posterior of the medulla terminalis (rMTp) which we named "lateroposterior" cells. In the cell body rind posterior to the hemi-ellipsoid body/ medulla terminalis (rHEp/MTp) were 4 "dorsal" cells, one of which was larger than the others and we refer to as "large dorsal" cell. In the dorsal region of the cell body rind posterior of the MP (rMPp) ~4-6 "ventroposterior" cells. In the anterior aspect of the brain, we found fewer cells; in the cell body rind lateroanterior of the hemi-ellipsoid body/medulla terminalis (rHE/MTla) was a single pair of cells, the "dorsoanterior" cells. A single "lateroanterior" cell was in the rind ventral and anterior of the lobula (rLOva). Finally, a pair of cells on the midline (one per hemisphere), anterior of the medial protocerebrum were named "medioanterior" cells (rMPa). Cell groups and their given nomenclature are summarised in Table S1.

In *P. hawaiensis*, *per*- and *cry2*-enriched cells were found throughout the anterior and posterior aspects of cell body rind overlying MP and HE/MT. In the cell body rind posterior and lateral to the PB (rPBp) were ten *per*- and *cry2*-enriched somata that we assigned as "medioposterior" cells (Figure 2C, D). Dorsoanterior to these cells were three cells, two of which were closer to each other than the last one; these we termed "medial-2" and "medial-1", respectively, and collectively, the medial cells. In the cell body rind dorsal of the HE/MT (rHE/MTd), we identified two somata as the "dorsal-lateral" cells, and antero-medial to these cells were six cells that

we called the “dorsal” cells. On the anterior aspect of the brain, we identified one cell in the cell body rind anterior of the HE/MT (rHE/MTa) as “anterior-lateral” cells. Lastly, we found 8 – 9 cells on the anterior aspect of the medial protocerebrum (rPMa). We refer to these cells collectively as the “anterior-medial” cells and they can be divided into 4 groups. “Anterior-medial b” cells described two cells with distinctive location lateral to the myoarterial formation. Dorsal to the anterior-medial b cells were two cells, in which one was positioned medially to the other. These, we termed “anterior-medial a1” and “anterior-medial a2” cells, respectively. The “anterior-medial c” cells were a group of 4 – 5 cells located dorsal to the anterior-medial a1-2 cells.

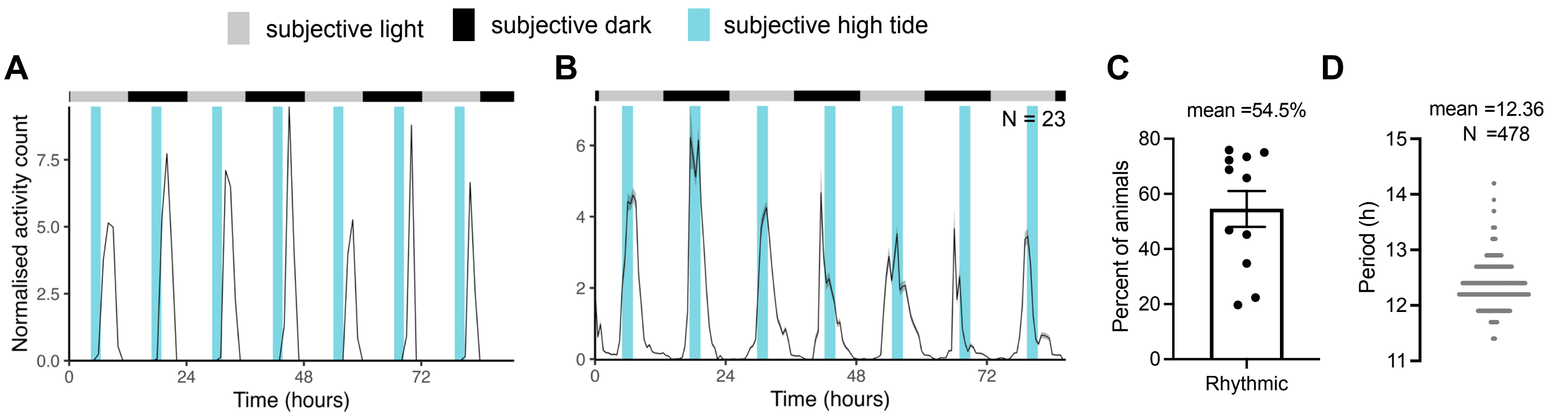
subjective light
subjective dark
subjective high tide
A
B
C
D
Normalised activity count
0.0
2.5
5.0
7.5
0
24
48
72
Time (hours)
N = 23
0
2
4
6
Time (hours)
mean =54.5%
Percent of animals
0
20
40
60
80
Rhythmic
mean =12.36
N =478
Period (h)
11
12
13
14
15

Figure S7. Circatidal swimming behaviour of beach-collected *Eurydice pulchra* recorded in free-running laboratory conditions. Related to Figure 3.

(A) Normalised swimming activity of a representative individual *E. pulchra*. Blue shadings indicate subjective high tides on home beach.

(B) Mean normalised swimming activity profile for a group of animals under DD from the same experiment as the individual in A (N =23).

(C) Percentage of animals showing rhythmic swimming behaviour from 11 independent experiments, representing 11 beach collections from 2022 to 2024. Error bars indicate SEM.

(D) Individual periods of beach-caught *E. pulchra* swimming activity under DD across all experiments. Mean (±SEM) is 12.36 ±0.02 h (N =478).

Lateroposterior cells

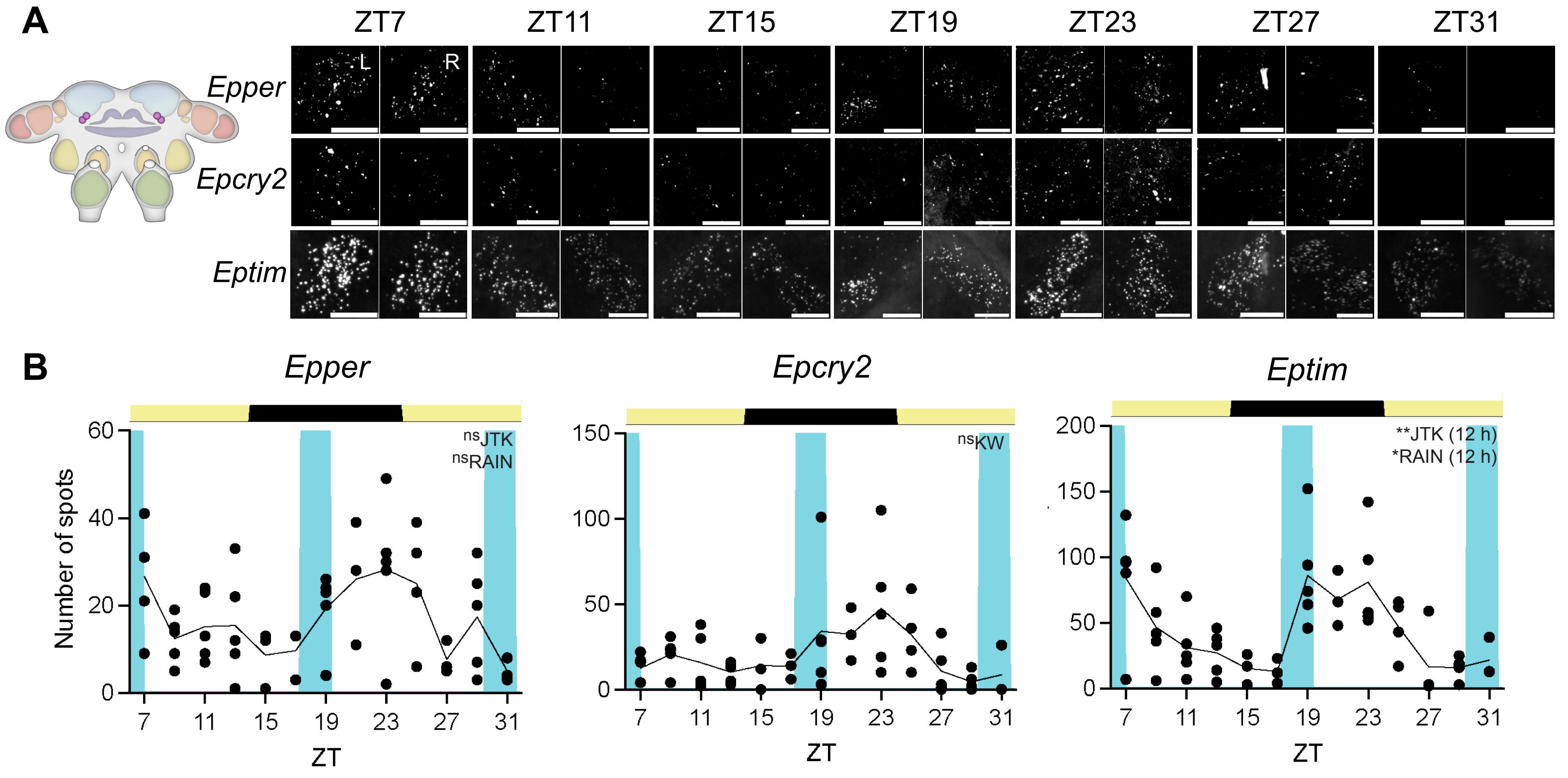

Figure S8. Time course of expression of *per, cry2* and *tim* in lateroposterior cells of tidally rhythmic *E. pulchra* held on a LD cycle. Related to Figure 3.

(A) Left: Cartoon to show location of lateroposterior cell group in the brain and Right: representative maximum intensity Z-projections of *Epper*, *Epcry2* and *Eptim* expression in lateroposterior cells in both hemispheres (L: left, R: right) across ZT (individual HCR-FISH channels in rows).

(B) Plots of mean transcript abundance, quantified as the number of FISH spots, across ZT for *Epper* (left), *Epcry2* (middle) and *Eptim* n (right) in the lateroposterior cells. n =3-5, N =55. Each point is from one brain, total across hemispheres. Inset text indicates statistical tests (JTK-cycle and RAIN) performed to determine 24-h and 12-h rhythmicity with the tested periods in parentheses, following significant time effect by ANOVA or Kruskal-Wallis. p <0.001***, p <0.01**, p ≤0.05*, p >0.05^{ns}. For statistics shown in this figure, refer to Table S2. Scale bars: 10 µm (A).

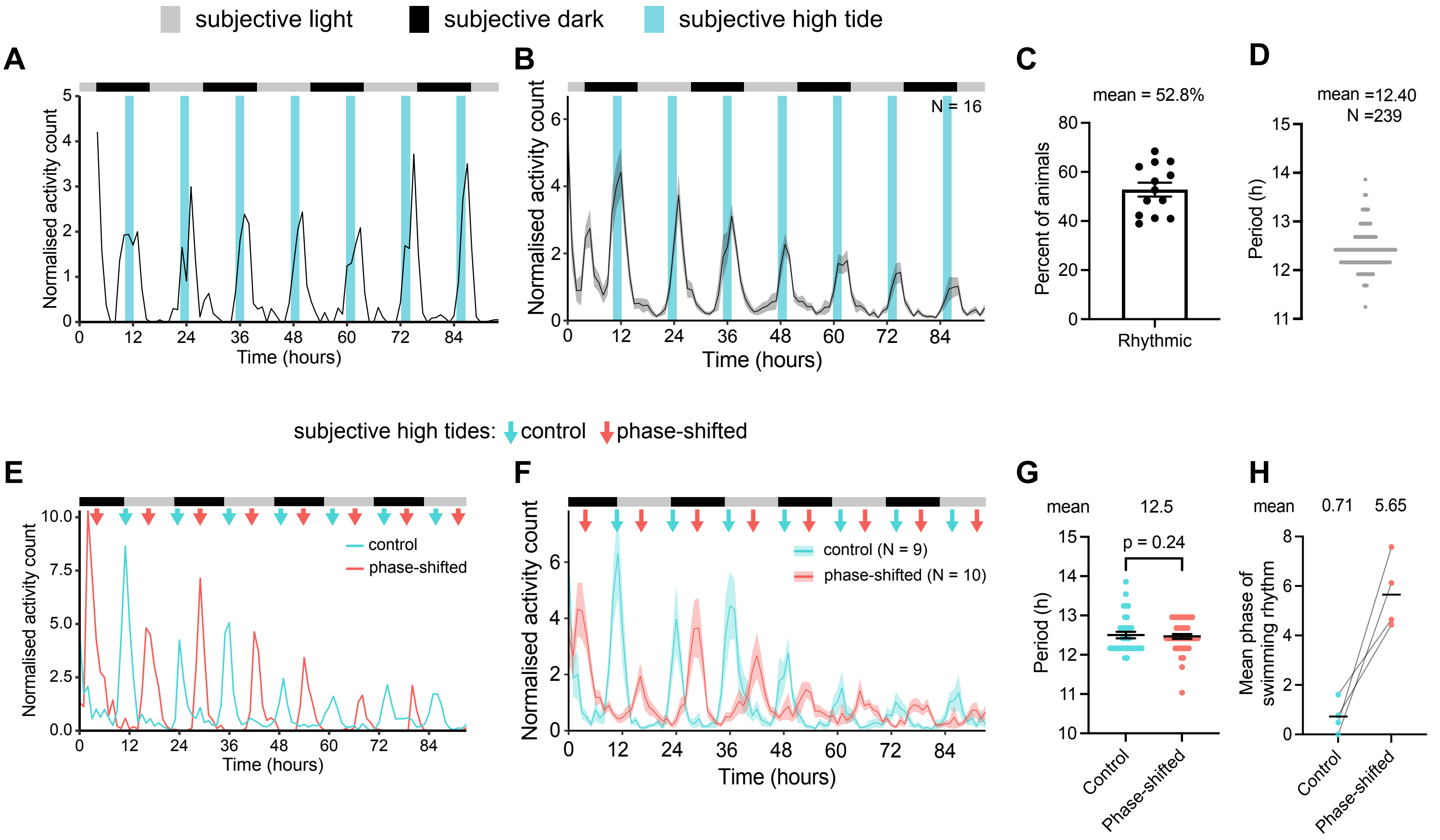

subjective light
subjective dark
subjective high tide
A
Normalised activity count
Time (hours)
B
N = 16
Normalised activity count
Time (hours)
C
mean = 52.8%
Percent of animals
Rhythmic
D
mean =12.40
N =239
Period (h)
subjective high tides: control phase-shifted
E
control
phase-shifted
Normalised activity count
Time (hours)
F
control (N = 9)
phase-shifted (N = 10)
Normalised activity count
Time (hours)
G
mean
12.5
p = 0.24
Period (h)
Control
Phase-shifted
H
mean
0.71
5.65
Mean phase of swimming rhythm
Control
Phase-shifted

Figure S9. Laboratory-cultured *P. hawaiensis* show circatidal activity rhythms after entrainment to a tidal cycle of 2 hours of mechanical agitation every 12.4 hours simulating high water. Related to Figure 4.

(A) Normalised swimming activity of individual laboratory-cultured *P. hawaiensis* free-running under DD (grey/ black bars), following entrainment to 2 h of agitation every 12.4 h. Blue bars indicate time of subjective tidal agitation.

(B) Mean normalised swimming activity profile for all circatidal rhythmic *P. hawaiensis* under DD (grey/ black bars) from the same experiment as the individual in A (N =16). Blue bars indicate time of subjective tidal agitation, shaded area indicates ±SEM.

(C) Percentage of animals showing a circatidal rhythm in swimming activity after tidal entrainment by agitation. Data from 13 independent experiments (N =456). Error bars indicate SEM.

(D) Individual periods of circatidal locomotor activity under DD of tidally entrained *P. hawaiensis.* Mean (±SEM) is 12.40 ±0.02 h (N =239).

(E) Representative activity recording of two individual laboratory-cultured *P. hawaiensis* free-running under DD (black/ grey bars) following prior tidal entrainment at a period of 12.4 h (cyan, control) or 12.9 h (red, phase-shifted) over 6 days. Arrows indicate times of subjective tidal agitation for the control and phase-shifted animals.

(F) Mean normalised swimming activity profile for all circatidally rhythmic *P. hawaiensis* under DD (grey/ black bars) from the same experiment as the individuals in E ($N_{control}$ =9, $N_{phase\text{-}shifted}$ =10). Arrows indicate times of subjective tidal agitation for the control and phase-shifted groups, shaded area indicates ±SEM.

(G). Individual periods of circatidal activity rhythm of *P. hawaiensis* after tidal entrainment by agitation under control and phase-shifted conditions (4 independent experiments, $N_{control}$ =34, $N_{phase\text{-}shifted}$ =42, Kolmogorov-Smirnov test: *D* =0.24, p =0.24, n.s.). Horizontal lines indicate mean phase within each group, and error bars indicate SEM.

(H) Mean phase of circatidal activity rhythms of paired control and phase-shifted groups across 4 replicate experiments as in F. The subjective high tide onset of the earliest control group defined phase =0. Horizontal lines indicate mean phase within each group.

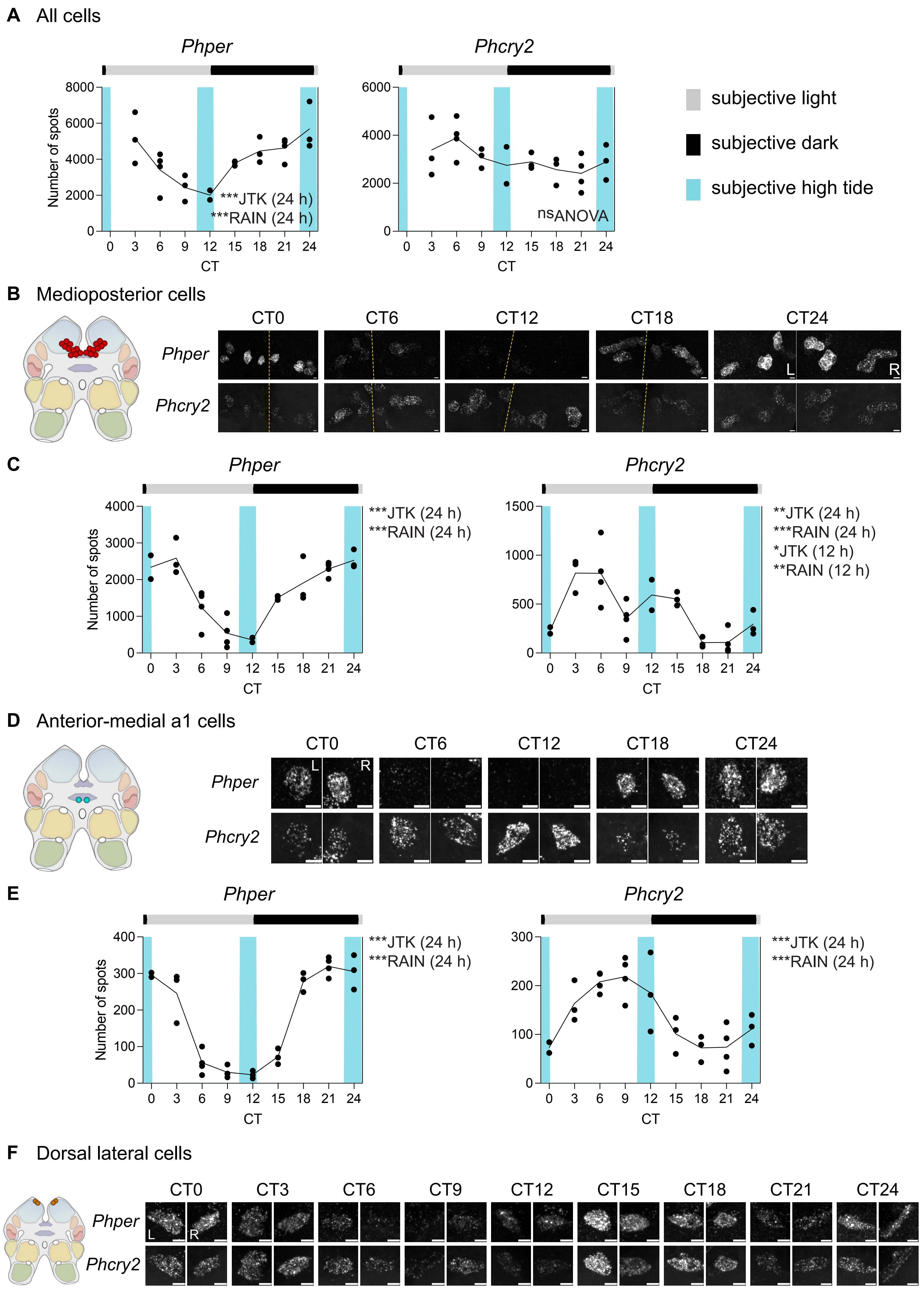
A All cells
Phper
Phcry2
subjective light
subjective dark
subjective high tide
Number of spots
***JTK (24 h)
***RAIN (24 h)
nsANOVA
CT
B Medioposterior cells
CT0
CT6
CT12
CT18
CT24
Phper
Phcry2
L
R
C
***JTK (24 h)
***RAIN (24 h)
**JTK (24 h)
***RAIN (24 h)
*JTK (12 h)
**RAIN (12 h)
D Anterior-medial a1 cells
E
***JTK (24 h)
***RAIN (24 h)
F Dorsal lateral cells
CT3
CT9
CT15
CT21

Figure S10. Free-running clock gene expression rhythms in tidally entrained *P. hawaiensis* (tidal phase θ + 1.2 h). Related to Figure 5.

(A) Mean aggregate expression, quantified as the number of FISH spots, of *Phper* and *Phcry2* across all cells plotted across time in tidally entrained animals sampled under DD. n =2-4, N =25.

(B) Left: Cartoon to show location of medioposterior cell group in the brain and Right: representative maximum intensity Z-projections of *Phper* and *Phcry2* expression in the medioposterior cells from both hemispheres (L: left, R: right, yellow dashed lines: midlines) across CT (individual HCR-FISH channels in rows).

(C) Mean transcript abundance, quantified as the number of FISH spots, across CT for *Phper* (left) and *Phcry2* (right)) in the medioposterior cells. n =2-4, N =28.

(D, E) As in B, C for anterior-medial a1 cell group. n =2-4, N =29.

(F) As in B for dorsal-lateral cells exhibiting a tidal pattern of expression (expanded from main Figure 5B).

Each point is from one brain, total across hemispheres. Inset text indicates statistical tests (JTK-cycle and RAIN) performed to determine 24-h and 12-h rhythmicity with the tested periods in parentheses, following significant time effect by ANOVA or Kruskal-Wallis. p <0.001***, p <0.01**, p ≤0.05*, p >0.05^{ns}. KW: Kruskal-Wallis test. For statistics shown in this figure, refer to Table S4. Scale bars: 10 μm (B), 5 μm (D, F).

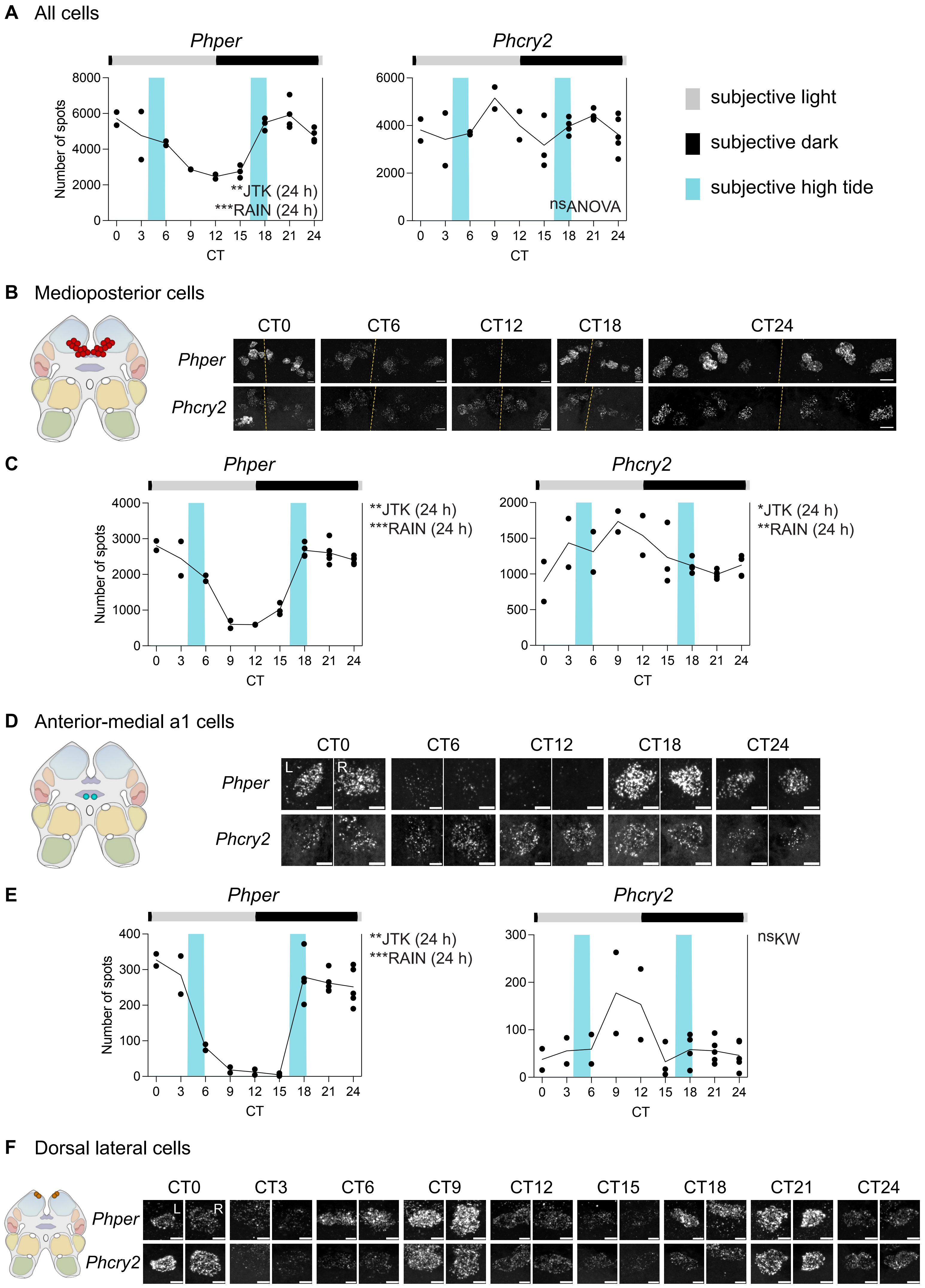
A All cells
Phper
Phcry2
Number of spots
CT
**JTK (24 h)
***RAIN (24 h)
nsANOVA
subjective light
subjective dark
subjective high tide
B Medioposterior cells
CT0
CT6
CT12
CT18
CT24
Phper
Phcry2
C
Phper
Phcry2
Number of spots
CT
**JTK (24 h)
***RAIN (24 h)
*JTK (24 h)
**RAIN (24 h)
D Anterior-medial a1 cells
CT0
CT6
CT12
CT18
CT24
L
R
Phper
Phcry2
E
Phper
Phcry2
Number of spots
CT
**JTK (24 h)
***RAIN (24 h)
nsKW
F Dorsal lateral cells
CT0
CT3
CT6
CT9
CT12
CT15
CT18
CT21
CT24
L
R
Phper
Phcry2

Figure S11. Free-running clock gene expression rhythms in tidally entrained *P. hawaiensis* (tidal phase θ +5.4 h). Related to Figure 5.

(A) Mean aggregate expression, quantified as the number of FISH spots, of *Phper* and *Phcry2* across all cells plotted across time in tidally entrained animals sampled under DD. n =2-5, N =26.

(B) Left: Cartoon to show location of medioposterior cells in the brain and Right: representative maximum intensity Z-projections of *Phper* and *Phcry2* expression in the anterior-medial a1 cells from both hemispheres (L: left, R: right, yellow dashed lines: midlines) across CT (individual HCR-FISH channels in rows).

(C) Mean expression, quantified as the number of FISH spots, across CT for *Phper* (left) and *Phcry2* (right) in the anterior-medial a1 cells. n =2-5, N =27.

(D, E) As in B, C for anterior-medial a1 cell group. n =2-5, N =27.

(F) As in B for dorsal-lateral cells exhibiting a tidal pattern of expression (expanded from main Figure 5D).

Each point is from one brain, total across hemispheres. Inset text indicates statistical tests (JTK-cycle and RAIN) performed to determine 24-h and 12-h rhythmicity with the tested periods in parentheses, following significant time effect by ANOVA or Kruskal-Wallis. $p < 0.001^{***}$, $p < 0.01^{**}$, $p \leq 0.05^{*}$, $p > 0.05^{ns}$. KW: Kruskal-Wallis test. For statistics shown in this figure, refer to Table S5. Scale bars: 10 µm (B), 5 µm (D, F).

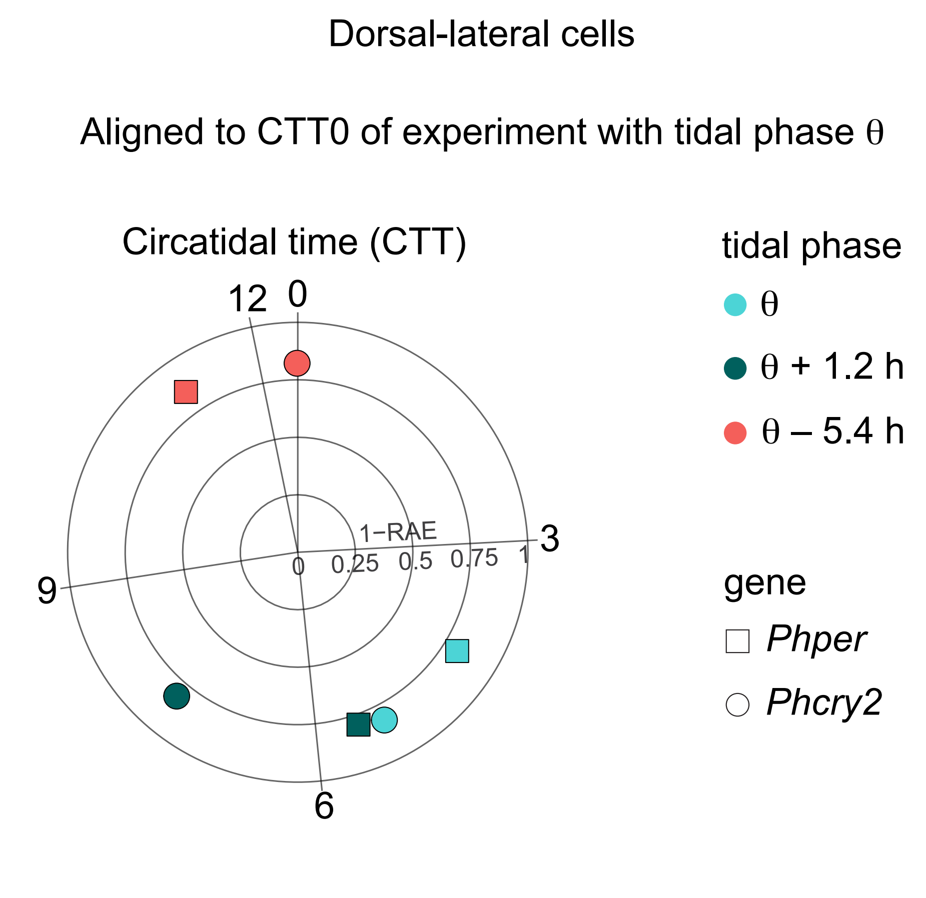

Dorsal-lateral cells
Aligned to CTT0 of experiment with tidal phase θ
Circatidal time (CTT)
0
3
6
9
12
1−RAE
0
0.25
0.5
0.75
1
tidal phase
θ
θ + 1.2 h
θ – 5.4 h
gene
Phper
Phcry2

Figure S12. Acrophase of free-running clock gene expression pattern in the dorsal lateral cells of *P. hawaiensis* entrained to tidal cycles of different phase. Related to Figures 5.

Circular plots of phase of circatidal peak expression of *Phper* and *Phcry2* in the dorsal-lateral cells of *P. hawaiensis* exhibiting circatidal activity rhythms. The phase of each experiment is aligned to circatidal time (CTT) =0 of the experiment with tidal phase θ. Each colour represents an individual experiment at each of different phases (total N =3 X 3). RAE: relative amplitude error.

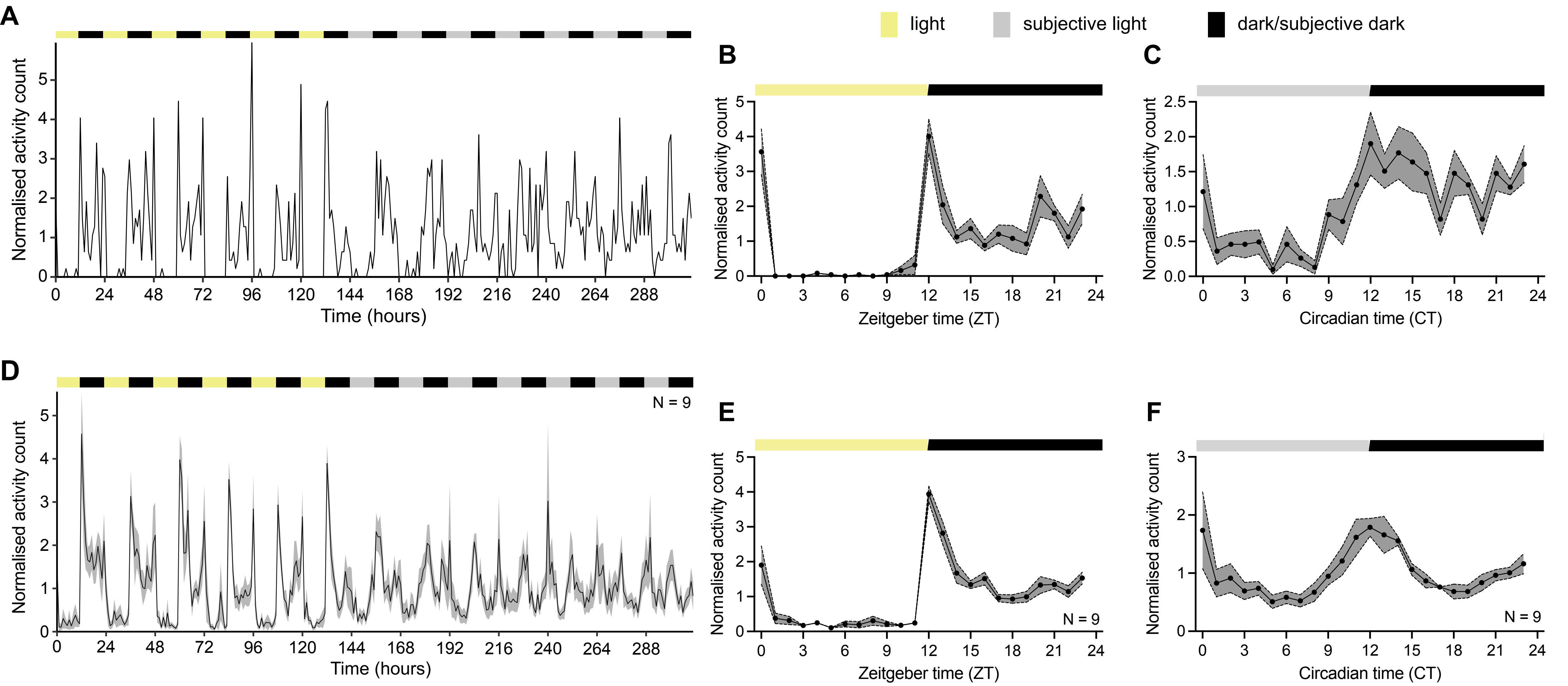
A
B
C
D
E
F
light
subjective light
dark/subjective dark
Normalised activity count
Time (hours)
Zeitgeber time (ZT)
Circadian time (CT)
N = 9

Figure S13. Laboratory-cultured *P. hawaiensis* show circadian activity rhythms after entrainment to LD cycle. Related to Figure 6.

(A) Representative activity recording of individual laboratory-cultured *P. hawaiensis*, which had never been subjected to tidal entrainment, initially maintained under 12 h:12 h L:D cycle (yellow/black bars) and then allowed to free-run under DD (grey/ black bars).

(B) Mean activity profile for the individual from A under LD showing higher activity during night-time. Shaded area indicates ±SEM.

(C) Mean activity profile for the individual from A under DD showing higher activity during subjective night. Shaded area indicates ±SEM.

(D, E and F) as for A, B and C but showing group data as mean ±SEM (shading, N =9).

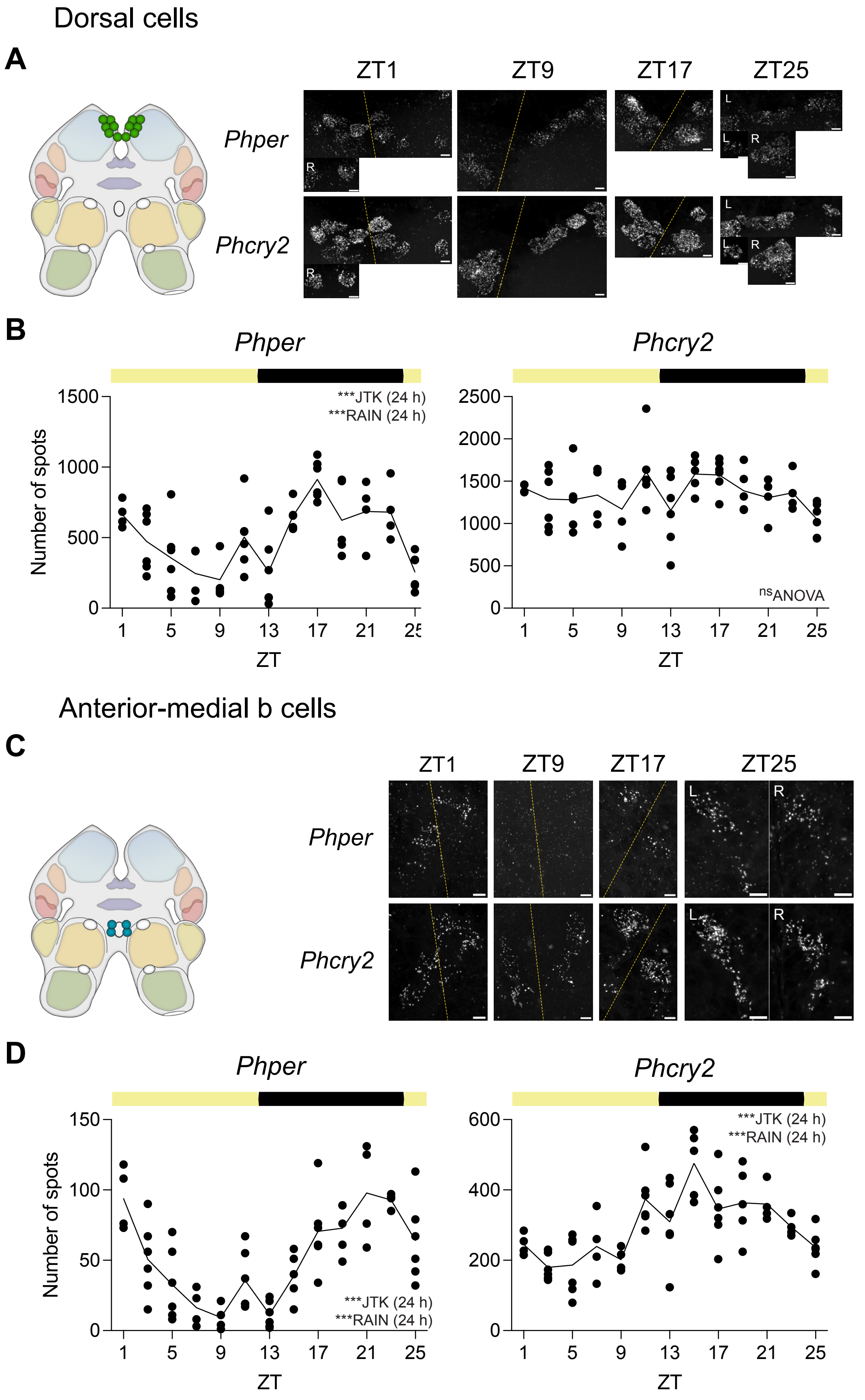

Dorsal cells
A
ZT1
ZT9
ZT17
ZT25
Phper
Phcry2
L
R
B
Phper
Phcry2
Number of spots
***JTK (24 h)
***RAIN (24 h)
nsANOVA
ZT
Anterior-medial b cells
C
ZT1
ZT9
ZT17
ZT25
Phper
Phcry2
L
R
D
Phper
Phcry2
Number of spots
***JTK (24 h)
***RAIN (24 h)
ZT

Figure S14. Time course of rhythmic expression of *per* and *cry2* in cells of *P. hawaiensis* synchronised to the LD cycle. Related to Figure 6.

(A) Left: Cartoon to show location of dorsal cell group in the brain and Right: representative maximum intensity Z-projections of *Phper* and *Phcry2* expression in the dorsal cells of both hemispheres (L: left, R: right, yellow dashed lines: midlines) across ZT (individual HCR-FISH channels in rows).

(B) Mean expression, quantified as the number of FISH spots, of *Phper* (left) and *Phcry2* in the dorsal cells plotted across daily time. n =4-6, N =66.

(C, D) as in A, B for anterior-medial b cell group. n =4-6, N =66.

Each point is from one brain, total across hemispheres. Inset text indicates statistical tests (JTK-cycle and RAIN) performed to determine 24-h and 12-h rhythmicity with the tested periods in parentheses, following significant time effect by ANOVA or Kruskal-Wallis. p <0.001***, p <0.01**, p ≤0.05*, p >0.05^{ns}. Scale bars: 5 μm. For statistics shown in this figure, refer to Table S6.

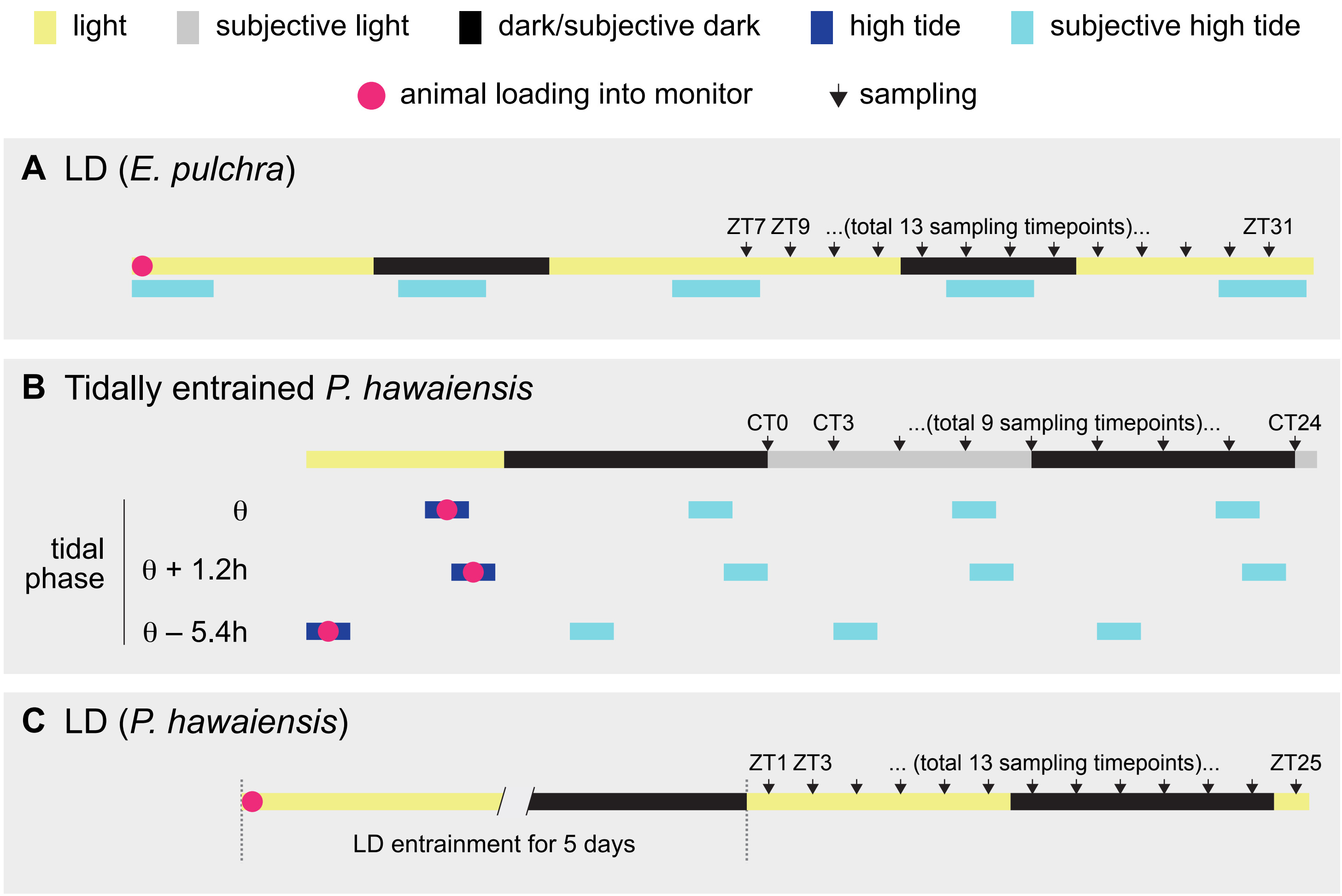
light
subjective light
dark/subjective dark
high tide
subjective high tide
animal loading into monitor
sampling
A LD (E. pulchra)
ZT7 ZT9 ...(total 13 sampling timepoints)... ZT31
B Tidally entrained P. hawaiensis
CT0 CT3 ...(total 9 sampling timepoints)... CT24
tidal phase
θ
θ + 1.2h
θ – 5.4h
C LD (P. hawaiensis)
ZT1 ZT3 ... (total 13 sampling timepoints)... ZT25
LD entrainment for 5 days

Figure S15. HCR-FISH time-course design. Related to Figures 3, 4 , 5 and 6.

(A) Field-collected *E. pulchra* were loaded into activity monitors and maintained under 16 h: 8 h LD cycles. At ZT7 on the day after loading, animals showing circatidal rhythmicity in their swimming activity were sampled every 2 hours, for a total of 13 timepoints.

(B) Laboratory-cultured *P. hawaiensis* which had been subjected to tidal cycles of mechanical agitations were loaded into activity monitors whilst being shaken. At CT0 on the day after loading, animals showing peaks in activity coinciding with the subjective high tide during the night before were sampled every 3 hours, for a total of 9 timepoints.

Time-course design is also described in Table S10.

(C) Laboratory-cultured *P. hawaiensis* that had never been tidally entrained were loaded into activity monitors and maintained under 12 h: 12 h LD cycles for 5 days. Animals showing 24-h rhythmicity over the 5-day LD entrainment period were sampled every 2 hours beginning at ZT1 on day 6, for a total of 13 timepoints.

Time-course design is also described in Table S10.

E. pulchra				*P. hawaiensis*		
Cell group	# of cells	Anatomical location		Cell group	# of cells	Anatomical location
Medioposterior	18	rPBp		Medioposterior	20	rPBp
Medial	10	rPBdp		Medial	6	rPBp
Dorsal	8	rHE/MTp		Dorsal	12	rHE/MTd
Medioanterior	2	rMPa		Dorsal-lateral	4	rHE/MTd
Lateroposterior	4	rMTp		Anterior-lateral	2	rHE/MTa
Dorsoanterior	4	rHE/MTa		Anterior-medial a1	2	rMPa
Lateroanterior	2	rLOva		Anterior-medial a2	2	rMPa
Ventroposterior	8-12	rMPp		Anterior-medial b	4	rMPa
				Anterior-medial c	8-10	rMPa

Table S1. Summary of cell groups enriched for *per* and *cry2* expression in *E. pulchra* and *P. hawaiensis*. Related to Figures 1, 2.

The anatomical location of each cell group was defined as the relative position of their resident cell body rind to anatomical structure of the brain and is abbreviated as follows: r: cell body rind, a: anterior, p: posterior, v: ventral, d: dorsal, HE/MT: hemi-ellipsoid body/ medulla terminalis, PB: protocerebral bridge, MP: medial protocerebrum, LO: lobula.

Groups with daily/ circadian or tidal rhythms across studies. See Tables S2- 6 for statistical details.

Note: because of its location, the ventroposterior cell group in *E. pulchra* could not be imaged in the time-course studies.

Gene	Cell group	Significant variation with time					24-h rhythmicity		12-h rhythmicity	
		Test	df	Test statistic	p-value	Effect size	JTK_cycle	RAIN	JTK_cycle	RAIN
Epper	all clock cells	ANOVA	12	2.590	0.011	0.425	0.003	0.001	1.000	0.852
	medioposterior	ANOVA	12	2.522	0.014	0.431	<0.001	0.001	1.000	0.990
	medial	ANOVA	12	2.617	0.011	0.428	0.022	0.003	1.000	0.711
	large-dorsal	ANOVA	12	2.090	0.041	0.391	0.003	0.192	1.000	1.000
	lateroanterior	KW	12	20.792	0.054	0.187				
	lateroposterior	ANOVA	12	2.200	0.030	0.386	0.459	1.000	1.000	0.999
	dorsoanterior	ANOVA	12	2.346	0.020	0.390	0.602	1.000	0.943	1.000
	dorsal	ANOVA	12	1.718	0.100	0.346				
Epcry2	all clock cells	KW	12	24.662	0.017	0.301	<0.001	<0.001	1.000	0.747
	medioposterior	ANOVA	12	2.563	0.013	0.435	<0.001	<0.001	1.000	0.886
	medial	KW	12	19.532	0.076	0.179				
	large-dorsal	KW	12	26.095	0.010	0.361	<0.001	0.017	1.000	0.996
	lateroanterior	KW	12	18.405	0.104	0.133				
	lateroposterior	KW	12	19.553	0.076	0.176				
	dorsoanterior	KW	12	14.737	0.256	0.061				
	dorsal	KW	12	16.606	0.165	0.118				

Gene	Cell group	Significant variation with time					24-h rhythmicity		12-h rhythmicity	
		Test	df	Test statistic	p-value	Effect size	JTK_cycle	RAIN	JTK_cycle	RAIN
Eptim	all clock cells	KW	12	26.588	0.009	0.347	0.034	0.009	0.034	0.008
	medioposterior	KW	12	20.747	0.054	0.219				
	medial	KW	12	29.999	0.003	0.429	0.170	0.028	0.046	0.005
	large-dorsal	KW	12	17.331	0.138	0.137				
	lateroanterior	KW	12	40.436	<0.001	0.592	0.005	0.001	0.092	0.002
	lateroposterior	KW	12	31.599	0.002	0.456	0.556	1.000	0.009	0.042
	dorsoanterior	KW	12	30.541	0.002	0.412	0.539	0.545	0.169	0.050
	dorsal	KW	12	19.182	0.084	0.184				

Table S2. Statistical tests for all gene-cell group combinations for the HCR-FISH time-course experiment of tidally active, beach-collected *E. pulchra* under LD. Related to Figure 3 and Figure S8. Effect size quantified as eta squared. KW: Kruskal-Wallis test. Cell groups with statistically significant periodicities.

Gene	Cell group	Significant variation with time					24-h rhythmicity		12-h rhythmicity		Cosinor fit	
		Test	df	Test statistic	p-value	Effect size	JTK_cycle	RAIN	JTK_cycle	RAIN	phase	RAE
Phper	all clock cells	ANOVA	8	11.99	<0.001	0.813	<0.001	<0.001	0.299	0.083	22.33	0.16
	mediopposterior	ANOVA	8	62.35	<0.001	0.954	<0.001	<0.001	0.381	0.262	22.32	0.07
	anterior-medial a1	ANOVA	8	43.95	<0.001	0.934	<0.001	<0.001	0.388	0.793	22.31	0.06
	anterior-medial b	ANOVA	8	2.253	0.059	0.429						
	anterior-medial c	ANOVA	8	1.856	0.125	0.426						
	dorsal	ANOVA	8	1.962	0.099	0.406						
	anterior-lateral	ANOVA	8	1.090	0.403	0.267						
	anterior-medial a2	ANOVA	8	2.157	0.068	0.408						
	medial-1	ANOVA	8	1.031	0.441	0.256						
	medial-2	ANOVA	8	1.180	0.353	0.291						
	dorsal-lateral	ANOVA	8	6.584	<0.001	0.678	1	0.034	0.006	<0.001	4.20	0.18
Phcry2	all clock cells	ANOVA	8	3.942	0.005	0.589	0.441	0.023	0.087	0.004		
	medioposterior	ANOVA	8	15.13	<0.001	0.835	<0.001	<0.001	0.059	0.002	9.35	0.14
	anterior-medial a1	ANOVA	8	9.814	<0.001	0.758	<0.001	<0.001	0.930	0.567	8.86	0.14
	anterior-medial b	ANOVA	8	1.501	0.209	0.333						
	anterior-medial c	KW	8	7.572	0.476	−0.021						
	dorsal	ANOVA	8	1.391	0.252	0.326						
	anterior-lateral	ANOVA	8	0.7758	0.628	0.205						
	anterior-medial a2	ANOVA	8	0.4122	0.903	0.117						
	medial-1	ANOVA	8	1.387	0.252	0.316						
	medial-2	KW	8	8.827	0.357	0.036						
	dorsal-lateral	ANOVA	8	6.866	<0.001	0.687	1	0.086	0.002	<0.001	5.26	0.18

Table S3. Statistical tests and cosinor analysis (with defined periods of 24-h and/or 12.4-h, whenever appropriate) for all gene-cell group combinations for the HCR-FISH time-course experiment of *P. hawaiensis* entrained to tidal phase θ. Related to Figure 4 and Table S7. Effect size quantified as eta squared. Phase derived from cosinor fit is corrected to Circadian Time (CT) =0 or Circatidal Time (CTT) =0, as appropriate. KW: Kruskal-Wallis test; RAE: relative amplitude error. Cell groups with statistically significant periodicities.

Gene	Cell group	Significant variation with time					24-h rhythmicity		12-h rhythmicity		Cosinor fit	
		Test	df	Test statistic	p-value	Effect size	JTK_cycle	RAIN	JTK_cycle	RAIN	phase	RAE
Phper	all clock cells	ANOVA	7	5.289	0.002	0.685	<0.001	<0.001	1	0.841	23.03	0.18
	mediopposterior	ANOVA	8	12.96	<0.001	0.845	<0.001	<0.001	0.772	0.462	22.90	0.11
	anterior-medial a1	ANOVA	8	49.29	<0.001	0.952	<0.001	<0.001	1	0.753	22.17	0.06
	anterior-medial b	KW	8	16.20	0.040	0.304	0.232	0.003	0.772	0.106		
	anterior-medial c	ANOVA	7	1.255	0.335	0.369						
	dorsal	ANOVA	8	0.7518	0.648	0.273						
	anterior-lateral	ANOVA	7	2.212	0.083	0.462						
	anterior-medial a2	ANOVA	8	1.732	0.152	0.410						
	medial-1	ANOVA	8	0.5012	0.84	0.182						
	medial-2	ANOVA	7	0.4214	0.876	0.148						
	dorsal-lateral	ANOVA	8	7.212	<0.001	0.743	0.232	0.003	0.016	<0.001	4.53	0.21
Phcry2	all clock cells	ANOVA	7	1.397	0.269	0.365						
	medioposterior	ANOVA	8	7.967	<0.001	0.770	0.005	<0.001	0.023	0.001	7.01	0.24
	anterior-medial a1	ANOVA	8	6.807	<0.001	0.731	<0.001	<0.001	1	0.773	7.88	0.13
	anterior-medial b	ANOVA	8	2.880	0.026	0.535	0.222	0.014	1	0.460		
	anterior-medial c	ANOVA	7	0.3891	0.894	0.154						
	dorsal	ANOVA	8	0.9256	0.522	0.316						
	anterior-lateral	ANOVA	7	0.6081	0.742	0.191						
	anterior-medial a2	ANOVA	8	1.667	0.168	0.400						
	medial-1	ANOVA	8	0.6999	0.688	0.237						
	medial-2	ANOVA	7	1.691	0.178	0.410						
	dorsal-lateral	ANOVA	8	4.908	0.002	0.663	1	0.215	0.016	<0.001	6.58	0.18

Table S4. Statistical tests and cosinor analysis (with defined periods of 24-h and/ or 12.4-h, whenever appropriate) for all gene-cell group combinations for the HCR-FISH time-course experiment of *Parhyale hawaiensis* entrained to tidal phase θ +1.2 h. Related to

Figure 5, Figure S10 and Table S7. Effect size quantified as eta squared. Phase derived from cosinor fit is corrected to Circadian Time (CT) =0 or Circatidal Time (CTT) =0, as appropriate. KW: Kruskal-Wallis test; RAE: relative amplitude error. Cell groups with statistically significant periodicities.

Gene	Cell group	Significant variation with time					24-h rhythmicity		12-h rhythmicity		Cosinor fit	
		Test	df	Test statistic	p-value	Effect size	JTK_cycle	RAIN	JTK_cycle	RAIN	phase	RAE
Phper	all clock cells	KW	8	19.98	0.010	0.705	0.002	<0.001	0.627	0.25	22.32	0.16
	mediopposterior	KW	8	20.39	0.009	0.689	0.002	<0.001	1	0.879	22.57	0.11
	anterior-medial a1	ANOVA	8	23.67	<0.001	0.913	0.005	<0.001	0.829	0.654	22.73	0.12
	anterior-medial b	ANOVA	8	1.912	0.121	0.096						
	anterior-medial c	KW	8	9.893	0.273	0.111						
	dorsal	ANOVA	8	1.808	0.145	0.460						
	anterior-lateral	KW	8	11.99	0.152	0.234						
	anterior-medial a2	KW	8	6.759	0.563	−0.073						
	medial-1	KW	8	11.56	0.172	0.223						
	medial-2	KW	8	7.392	0.495	-0.036						
	dorsal-lateral	ANOVA	8	7.469	<0.001	0.768	1	0.277	<0.001	<0.001	4.40	0.15
Phcry2	all clock cells	ANOVA	8	1.544	0.215	0.421						
	medioposterior	ANOVA	8	2.537	0.048	0.530	0.016	0.001	1	0.84	9.06	0.23
	anterior-medial a1	KW	8	10.06	0.261	0.114						
	anterior-medial b	ANOVA	8	1.129	0.391	0.334						
	anterior-medial c	ANOVA	8	2.131	0.090	0.501						
	medial-1	ANOVA	8	1.497	0.234	0.428						
	medial-2	ANOVA	8	0.8941	0.542	0.296						
	dorsal	KW	8	13.51	0.096	0.324						
	anterior-lateral	ANOVA	8	4.823	0.003	0.694	1	0.203	<0.001	<0.001	3.93	0.17
	anterior-medial a2	KW	8	12.26	0.14	0.251						
	dorsal-lateral	ANOVA	8	8.943	<0.001	0.799	0.758	0.085	<0.001	<0.001	5.59	0.18

Table S5. Statistical tests and cosinor analysis (with defined periods of 24-h and/ or 12.4-h, whenever appropriate) for all gene-cell group combinations for the HCR-FISH time-course experiment of *P. hawaiensis* entrained to tidal phase θ – 5.4 h. Related to Figure 5,

Figure S11 and Table S7. Effect size quantified as eta squared. Phase derived from cosinor fit is corrected to Circadian Time (CT) =0 or Circatidal Time (CTT) =0, as appropriate. KW: Kruskal-Wallis test; RAE: relative amplitude error. Cell groups with statistically significant periodicities.

Gene	Cell group	Significant variation with time					24-h rhythmicity		12-h rhythmicity		Cosinor fit	
		Test	df	Test statistic	p-value	Effect size	JTK_cycle	RAIN	JTK_cycle	RAIN	phase	RAE
Phper	all clock cells	ANOVA	12	14.28	<0.001	0.764	<0.001	<0.001	1	0.250	21.14	0.11
	medioposterior	KW	12	54.45	<0.001	0.801	<0.001	<0.001	1	0.515	21.62	0.07
	anterior-medial a1	KW	12	57.04	<0.001	0.850	<0.001	<0.001	1	0.717	21.05	0.09
	anterior-medial b	ANOVA	12	9.073	<0.001	0.673	<0.001	<0.001	1	0.791	21.55	0.11
	anterior-medial c	ANOVA	12	1.697	0.095	0.285						
	dorsal	ANOVA	12	6.225	<0.001	0.585	<0.001	<0.001	1	0.193	19.11	0.18
	anterior-lateral	KW	12	13.09	0.363	0.021						
	anterior-medial a2	KW	12	20.94	0.051	0.172						
	medial-1	KW	12	24.66	0.017	0.239	1	0.868	1	0.014		
	medial-2	KW	12	20.18	0.064	0.157						
	dorsal-lateral	ANOVA	12	3.973	<0.001	0.474	0.103	0.003	1	0.051		
Phcry2	all clock cells	KW	12	26.16	0.010	0.267	0.05	0.001	1	0.019	11.70	0.36
	medioposterior	ANOVA	12	12.40	<0.001	0.737	<0.001	<0.001	0.014	<0.001	9.03	0.16
	anterior-medial a1	KW	12	41.79	<0.001	0.562	<0.001	<0.001	1	0.219	8.28	0.12
	anterior-medial b	ANOVA	12	6.574	<0.001	0.598	<0.001	<0.001	1	0.580	16.39	0.14
	anterior-medial c	ANOVA	12	2.203	0.026	0.341	0.049	0.009	1	0.034	15.78	0.34
	dorsal	ANOVA	12	1.792	0.074	0.289						
	anterior-lateral	ANOVA	12	0.9371	0.518	0.178						
	anterior-medial a2	KW	12	15.15	0.233	0.061						
	medial-1	ANOVA	12	1.233	0.286	0.218						
	medial-2	ANOVA	12	2.057	0.037	0.322	1	0.951	1	0.989		
	dorsal-lateral	ANOVA	12	1.501	0.153	0.254						

Table S6. Statistical tests and cosinor analysis (with defined periods of 24-h whenever appropriate) for all gene-cell group combinations for the HCR-FISH time-course experiment of *P. hawaiensis* synchronised to and sampled under LD. Related to Figure 6 and Figure

S14. Effect size quantified as eta squared. Phase derived from cosinor fit is corrected to Zeitgeber Time (ZT) =0. KW: Kruskal-Wallis test; RAE: relative amplitude error. Cell groups with statistically significant periodicities.

Gene	Cell group	Mean phase	Mean vector length	Number of experiments
Circadian oscillations				
Phper	All clock cells	22.56	1.00	3
	Medioposterior	22.60	1.00	3
	Anterior-medial a1	22.40	1.00	3
Phcry2	Medioposterior	8.48	0.96	3
	Anterior-medial a1	8.37	0.99	2
Circatidal oscillations				
Phper	Dorsal-lateral	4.38	1.00	3
Phcry2		5.81	0.96	3

Table S7. Circular statistics of gene-cell group combinations that show statistically significant rhythmicity (at 24-h or 12-h) across 3 HCR-FISH time-course experiments of tidally entrained *P. hawaiensis*. Related to Figures 4, 5, 7, Figures S10, 11, Tables S3 -5.

Probe/target genes	NCBI accession #	Probe hairpin	Number of probe pairs	Lot number	Relevant figure(s)
EpBmal1	KC885968.1	B3	20	PRP939	Fig. 1, Fig. S3-4
EpBmal1 additional	KC885968.1	B3	40	RTA402	Fig. 1, Fig. S3-4
EpClk 1-9	KC885973.1	B1	40	RTA400	Fig. 1, Fig. S3
EpClk 1-9 additional	KC885973.1	B1	29	RTE285	Fig. 1, Fig. S3
Epcry2	KC885970.1	B1	20	RTA399	Fig. 1, Fig. S3, 6
Epcry2	KC885970.1	B3	20	RTO453	Fig. 1, Fig. S8
Epper	KC885967.1	B2	20	RTO454	Fig. 1, 3, Fig. S3, 8
Eptim	KC885969.1	B1	20	PRP937	Fig. 1, 3, Fig. S3, 8
PhBmal1	Not applicable. Sequences from Hunt, Mallon & Rosato (2019)	B1	38	RTC080	Fig. 1, Fig. S3-4
Phper		B1/B2	30	RTK407/RTC082	Fig. 1, 4-6, Fig. S3, 10-11, 13
PhClk-fragment 1		B2	40	RTG497	Fig. 1, 4-6 Fig. S3
PhClk-fragment 2		B2	28	RTG498	Fig. 1 Fig. S3
Phcry2		B3	30	RTC083	Fig. 1, 4-6, Fig. S3, 6, 10-11, 13
deGFP	Not applicable. Sequence provided by Molecular Instruments Inc.	B1	12	RTA210	Fig. S3
deGFP		B2	12	PRQ740	Fig. S3

Table S8. The targets, hairpin identities and lot numbers of the HCR-FISH primary probes used to generate the data described in this study. Relate to STAR Methods.

Probe/target gene	NCBI accession #	Amplifier	ACD cat number
Epper-C4	KC885967.1	C4	578211-C4
EpClk5-C1	KC885973.1	C1	578151-C1
EpBmal1-C2	KC885968.1	C2	578191-C2
-ve control			

* Negative control probe is designed against the DapB gene of *Bacillus subtilis* strain

Table S9. The names, hairpin identities and lot numbers of the RNAscope technology FISH probes. Related to STAR Methods.

Experiment	Sampling condition	First sampling time-point	*N* total animals sampled	Sampling interval	1st subjective high tide onset during sampling	HCR-FISH primary probes	HCR-FISH hairpins (AF: AlexaFluor)	Relevant figures/tables
14h:10h LD-maintained *E. pulchra* (Figure S15A)								
LD (*E. pulchra*)	LD	ZT7	65	2	ZT18.4	*Eptim*-B1, *Epper*-B2, *Epcry2*-B3	B1-AF647, B2-AF546, B3-AF488	Figure 3, Figure S8, Table S2
Tidally entrained *P. hawaiensis* (Figure S15B)								
Tidal θ *P. hawaiensis*	DD	CT0	37	3	CT9.2	*Phper*-B2, *Phcry2*-B3	B2-AF546, B3-AF488,	Figure 4, Table S3
Tidal θ + 1.2h *P. hawaiensis*	DD	CT0	35	3	CT10.4	*Phper*-B2, *Phcry2*-B3	B2-AF647, B3-AF488,	Figure 5, Figure S10, Table S4
Tidal θ – 5.4h *P. hawaiensis*	DD	CT0	35	3	CT3.8	*Phper*-B2, *Phcry2*-B3	B2-AF647, B3-AF488,	Figure 5, Figure S11, Table S5
12h:12h LD-synchronised *P. hawaiensis* (Figure S15C)								
LD (*P. hawaiensis*)	LD	ZT1	79	2	N.A.	*Phper*-B1, *Phcry2*-B3	B1-AF647, B4-AF647	Figure 6, Figure S13, Table S6

Table S10. FISH time-course designs for investigating clock gene expression rhythms. Related to Figures 3-6, Figures S10, S11, S13, S15 and Tables S2-S6.
