# Supplementary material for: Expression of clock genes tracks daily and tidal time in brains of intertidal crustaceans Eurydice pulchra and Parhyale hawaiensis
Source: Curr Biol. Author manuscript; Available in PMC 2025 Jul 30. (PMC7617966; doi:10.1016/j.cub.2025.04.047)
Supplement: Document S2. [file EMS207343-supplement-Document_S2_.pdf]

Current Biology

Article

Expression of clock genes tracks daily and tidal time in brains of intertidal crustaceans *Eurydice pulchra* and *Parhyale hawaiensis*

Graphical abstract

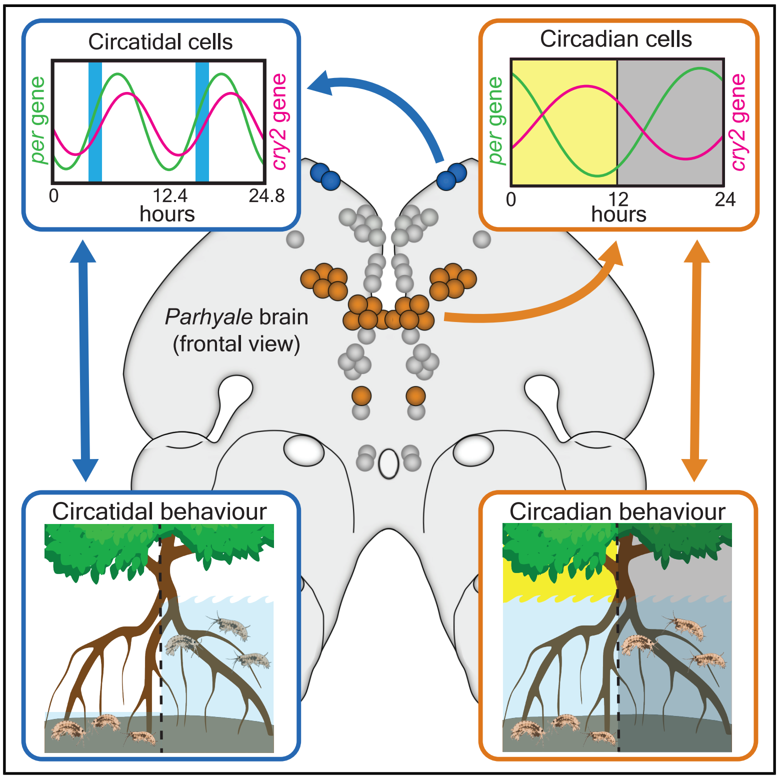

Highlights

- Circadian clock gene expression mapped in brains of two tidally rhythmic crustaceans
- Discrete populations of ∼60 putative clock cells identified in both species
- Separate cells in same brain exhibit either daily or tidal clock gene expression
- Canonical circadian clock genes implicated in circatidal clockwork

Authors

Andrew Oliphant, Chee Y. Sia,
Charalambos P. Kyriacou,
David C. Wilcockson,
Michael H. Hastings

Correspondence

mha@mrc-lmb.cam.ac.uk

In brief

Oliphant and Sia et al. explore the basis of tidal clocks in crustaceans by mapping canonical circadian clock gene expression across *Eurydice* and *Parhyale* brains, revealing comparable clusters of ∼60 putative clock cells. Daily and tidal expression rhythms in different cells in the same brains implicate circadian clock genes in circatidal clockwork.

Oliphant et al., 2025, Current Biology *35*, 2802–2815
June 23, 2025 © 2025 MRC Laboratory of Molecular Biology.
Published by Elsevier Inc.
https://doi.org/10.1016/j.cub.2025.04.047

CellPress

CellPress
OPEN ACCESS

Current Biology

Article

Expression of clock genes tracks daily and tidal time in brains of intertidal crustaceans *Eurydice pulchra* and *Parhyale hawaiensis*

Andrew Oliphant,[1,4] Chee Y. Sia,[1,4] Charalambos P. Kyriacou,[2] David C. Wilcockson,[3] and Michael H. Hastings[1,5,*]
[1]Neurobiology Division, MRC Laboratory of Molecular Biology, Francis Crick Avenue, Cambridge CB2 0QH, UK
[2]Department of Genetics, Genomics and Cancer Sciences, University of Leicester, University Road, Leicester LE1 7RH, UK
[3]Department of Life Sciences, Aberystwyth University, Penglais, Aberystwyth SY23 3DA, UK
[4]These authors contributed equally
[5]Lead contact
*Correspondence: mha@mrc-lmb.cam.ac.uk
https://doi.org/10.1016/j.cub.2025.04.047

SUMMARY

Intertidal organisms, such as the crustaceans *Eurydice pulchra* and *Parhyale hawaiensis*, express daily and tidal rhythms of physiology and behavior to adapt to their temporally complex environments. Although the molecular-genetic basis of the circadian clocks driving daily rhythms in terrestrial animals is well understood, the nature of the circatidal clocks driving tidal rhythms remains a mystery. Using *in situ* hybridization, we identified discrete clusters of ∼60 putative "clock" cells co-expressing canonical circadian clock genes across the protocerebrum of *E. pulchra* and *P. hawaiensis* brains. In field-collected, tidally rhythmic *E. pulchra* sampled under a light:dark (LD) cycle, the expression of *period* (*per*) and *cryptochrome 2* (*cry2*) exhibited daily rhythms in particular cell groups, whereas *timeless* (*tim*) showed 12-h rhythms in others. In tidally rhythmic laboratory-reared *P. hawaiensis*, previously entrained to 12.4-h cycles of agitation under LD and sampled under continuous darkness, several cell groups (e.g., medioposterior cells) exhibited circadian expression of *per* and *cry2*. In contrast, dorsal-lateral cells in the protocerebrum exhibited robust ∼12-h, i.e., circatidal, rhythms of *per* and *cry2*, phased to the prior tidal agitation but not the prior LD. In *P. hawaiensis* exhibiting daily behavior under LD without tidal agitation, robust daily rhythms of *per* and *cry2* expression were evident in medioposterior and other cells, whereas expression in dorsal-lateral cells was not rhythmic, underlining their essentially tidal periodicity. These results implicate canonical circadian molecules in circatidal timekeeping and reveal conserved brain networks as potential neural substrates for the generation of daily and tidal rhythms appropriate to intertidal habitats.

INTRODUCTION

Organisms adapt to their cyclical environments through rhythms of metabolism, physiology, and behavior that are driven by internal biological clocks.[1] Terrestrial environmental cycles are predominantly daily, leading to the evolution of clocks with an autonomous period of approximately 1 day (hence *circa-dian*) and entrained to the cycle of light and darkness. In contrast, coastal environments present greater temporal complexity and marine organisms have additionally evolved a range of circatidal (∼12.4 h), circasemilunar (∼14.8 days), and circalunar (∼29.5 days) timing mechanisms[2,3] that co-ordinate adaptive rhythms across these time domains. Despite their biological relevance, the mechanistic underpinnings of circatidal clocks remain a mystery and three principal hypotheses have been proposed.[2] First, circatidal and circadian rhythms are driven by a single oscillatory system, the period of which is plastic, adapting to the period of the dominant environmental cue.[4] Second, paired circalunidian clocks with an intrinsic period of ∼24.8 h (i.e., a slightly modified circadian period) run in antiphase to create sequential peaks 12.4-h apart.[5] Third, separate 12.4- and 24-h oscillators co-exist, independently driving circatidal and daily phenotypes.[6] Testing these models requires identification of the clock cells and genes responsible for tidal timekeeping.

Circadian clocks in animals pivot around a transcriptional/translational feedback loop (TTFL) in which positive factors CLOCK (CLK) and BMAL1 (or CYC in flies), acting through E-box promoter sites, transactivate genes that encode negative regulators, PERIOD (PER), CRYPTOCHROME2 (CRY2), and/or TIMELESS (TIM), depending on species.[7] Accumulating negative regulators progressively suppress transactivation and, following their degradation, the cycle restarts after ∼24 h.[8,9] The intertidal peracarid crustaceans *Eurydice pulchra* (Isopoda, the speckled sea louse) and *Parhyale hawaiensis* (Amphipoda) can express autonomous circadian and/or circatidal rhythms of locomotor behavior in the absence of environmental cues.[10–13] However, the only molecular markers of circatidal timekeeping relate to mitochondrial gene expression and cellular redox state in *E. pulchra*.[14] Nevertheless, genes encoding CLOCK, BMAL1, PER, and CRY2 (i.e., invertebrate type 2

 © 2025 MRC Laboratory of Molecular Biology. Published by Elsevier Inc.
This is an open access article under the CC BY license (http://creativecommons.org/licenses/by/4.0/).

CellPress
OPEN ACCESS

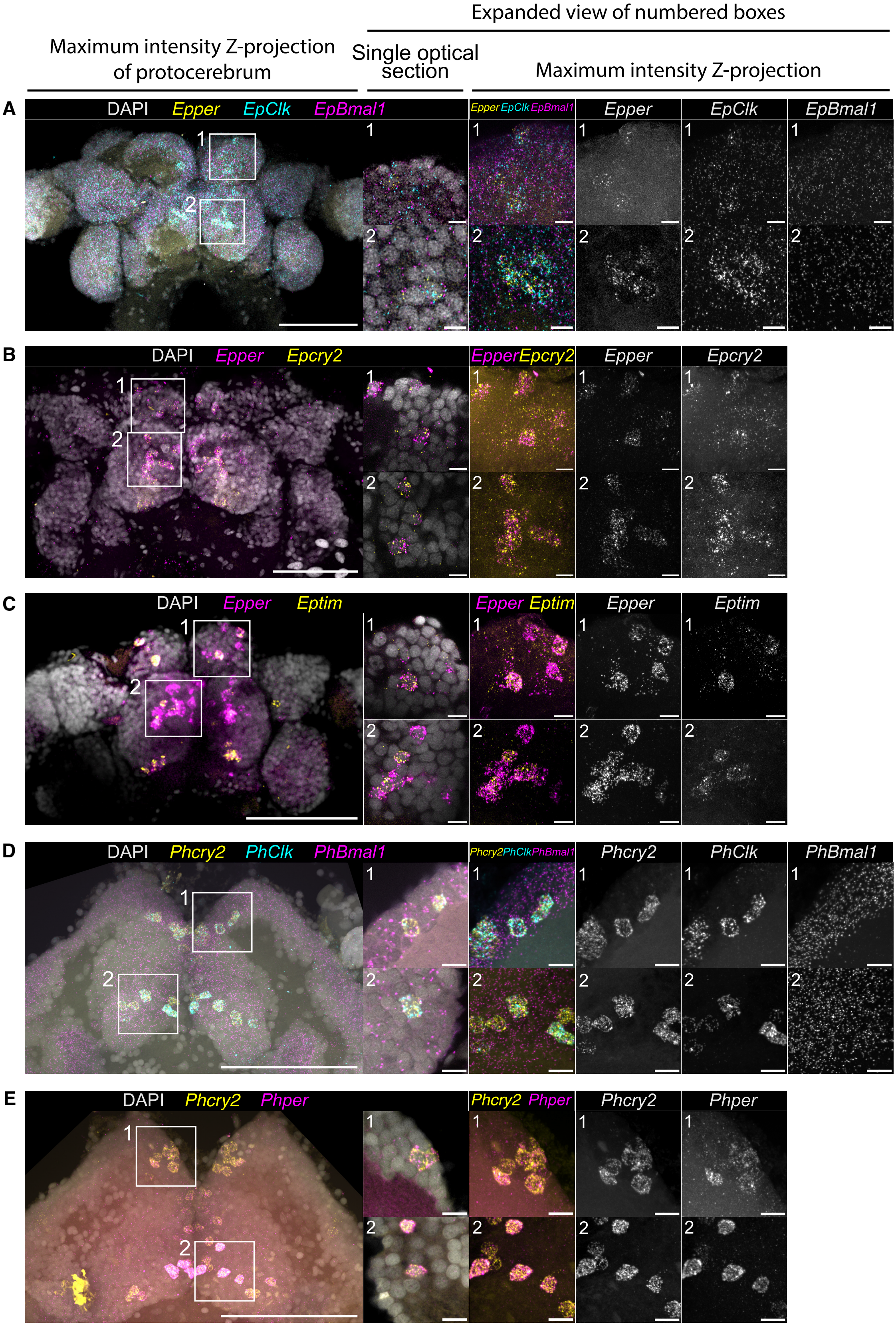

(legend on next page)

CRY) are present in both species, although the photosensitive invertebrate type 1 CRY is absent from both.[12,15] Furthermore, *tim* expression in the head of *E. pulchra* is circadian,[12,16] whereas *P. hawaiensis* lacks *tim*.[15] *E. pulchra* CLOCK:BMAL1 heterodimers can transactivate E-boxes and are suppressed by PER and CRY2,[12] whereas PhBMAL1 co-expressed with mouse CLK can also transactivate E-boxes.[13] Finally, genomic deletion (*P. hawaiensis*) or RNAi-knockdown (*E. pulchra*) of *Bmal1* compromises tidal activity rhythms,[13,16] raising the intriguing possibility of interactions between circadian and circatidal clocks at the molecular-genetic level.

The current aim was to describe the expression of *Bmal1*, *Clk*, *per*, and *cry2* (together with *Eptim*) across the brain to identify putative "clock" cells and then to test their relationship to daily, circadian, and circatidal behavior. We reveal ~60 such cells arranged in multiple, discrete cell groups in the brain and show that particular groups are capable of tracking daily/circadian time (CT), encoded as the rhythmic expression of canonical circadian genes. Furthermore, we also identify cells with 12-h rhythms of expression of *tim* in *E. pulchra* and of *per* and *cry2* in *P. hawaiensis* and show that the latter are capable of tracking circatidal time. These results implicate canonical circadian mechanisms in circatidal timekeeping. They also reveal brain networks that may represent neural substrates for the generation of interactive daily and tidal rhythms appropriate to local intertidal habitats.

RESULTS

The brains of *E. pulchra* and *P. hawaiensis* express canonical circadian clock genes

Our neuroanatomical descriptions of *E. pulchra* are based on the isopod *Saduria entomon*.[17] The brain structure of *P. hawaiensis* has been described.[18,19] To map gene expression across the brain, we first generated reference brains for *E. pulchra* and *P. hawaiensis* by averaging confocal image stacks of brains immunolabeled with anti-synapsin (SYNORF1) to reveal neuropils (Figure S1). We segmented the neuropils to facilitate description of gross brain anatomy and naming of clock cell groups relative to the body axis (Figures S1 and S2; Videos S1 and S2).

Bmal1 is implicated in circadian and circatidal timekeeping in both *E. pulchra* and *P. hawaiensis*. We therefore used whole-mount HCR-FISH to map *Bmal1* transcripts as a first step to identify putative clock cells. This revealed widespread expression across the brain of *E. pulchra* (Figure S3A) and, consistent with qPCR studies,[12] its intensity did not change over time in light:dark (LD) (Figure S4A). The expression of *Clk* mRNA was similarly widespread in the brain of *E. pulchra*, with most cells expressing low levels although there was a condensation of higher-intensity signals in ~60 cells, located principally in the median protocerebrum (Figure S3B). These unexpected, dispersed *Bmal1* and *Clk* expression patterns in *E. pulchra* were confirmed using an alternative RNA detection technology, RNAscope, on wax-embedded brain sections (Figure S5). There were no differences in the expression patterns of *EpBmal1* (dispersed), *EpClk* (dispersed with some cellular enrichment), and *Epper* (cellular enrichment, see below) detected by the two methods, but the HCR-FISH on whole-mounts was a more practical and convenient approach and so it was adopted for further studies.

Pan-cellular expression of *Bmal1* was also evident in the brain of *P. hawaiensis* (Figure S3C) and, again, this did not change across the LD cycle (Figure S4B). In contrast, the expression of *Clk* in *P. hawaiensis* was less widespread than in *E. pulchra*, with enrichment in cells in the median protocerebrum (Figure S3D). The enrichment was clearer than in *E. pulchra* and these enriched cells were comparable in number and location to those in *E. pulchra*. Thus, notwithstanding the requirement for *Bmal1* to sustain circatidal behavior in both species,[13,16] the pattern of its expression did not specifically signpost any populations of putative clock cells. In contrast, the expression of *Clk* was significantly elevated in discrete cell populations in the median protocerebrum of both species.

We then mapped the expression of genes encoding negative regulators *per*, *cry2*, and *tim* across the brain of *E. pulchra*. This revealed strong, specific signals for all three transcripts restricted to discrete cell clusters in the median protocerebrum (Figures S3E–S3G). In *P. hawaiensis* brains, the expression of *per* and *cry2* exhibited a pattern similar to that of *E. pulchra*, with high levels of both transcripts localized to ~60 cells in the median protocerebrum (Figures S3I and S3J). The HCR-FISH was specific, insofar as probes against *deGFP* gave no signal (Figures S3H and S3K).

Co-expression of circadian clock genes defines groups of putative clock cells in *E. pulchra* and *P. hawaiensis* brains

CLOCK:BMAL1 heterodimers are required for transactivation within the TTFL and so we compared the co-expression of *Bmal1* and *Clk* by multiplexed HCR-FISH of *E. pulchra* brains dissected in the light phase. The *Clk*-enriched cells expressed *Bmal1* at the low level seen in other cells across the brain. They also expressed high levels of *per* (Figure 1A) and the *per*-expressing cells also expressed *cry2* and *tim* (Figures 1B and 1C). Given

Figure 1. Cellular co-expression of circadian clock genes in the brains of *E. pulchra* and *P. hawaiensis*

(A) Representative maximum intensity Z-projections and single optical sections of *E. pulchra* brain probed for *Epper*, *EpClk*, and *EpBmal1* using HCR-FISH. Left: merged low-power (25×) view of protocerebrum of *E. pulchra*, center: merged high-power (40×) single optical sections in boxes labeled 1 and 2, right: single channel high-power maximum intensity Z-projections. *EpBmal1* is widely and uniformly expressed in both putative clock cells and other cells across the brain, but it is not enriched in cells enriched for *EpClk*, whereas *Epper* is enriched in *EpClk*-enriched cells.

(B) As in (A) for *Epper* and *Epcry2*, expression of which is co-enriched in putative clock cells but not in other cells in the brain of *E. pulchra*.

(C) As in (A) for *Epper* and *Eptim*, of *Epper*-enriched cells in *E. pulchra*.

(D) As in (A) but for *P. hawaiensis* brain probed for *PhBmal1*, *PhClk*, and *Phcry2* using HCR-FISH. *PhBmal1* is widely and uniformly expressed in both putative clock cells and other cells across the brain, but it is not enriched in cells enriched for *PhClk*, whereas *Phcry2* is enriched in *PhClk*-enriched cells.

(E) As in (A) but for *P. hawaiensis* brain labeled for *Phcry2* and *Phper*. *Phcry2* and *Phper* are co-enriched in putative clock cells, but not other cells, in the brain. Animals were sampled across various points in the light phase of a 14L:10D (*E. pulchra*) or 12L:12D (*P. hawaiensis*) cycle in the laboratory without reference to activity rhythms. Scale bars: 100 μm (left-most), 10 μm (other).

See also Figures S1–S6 and Table S1.

CellPress
OPEN ACCESS

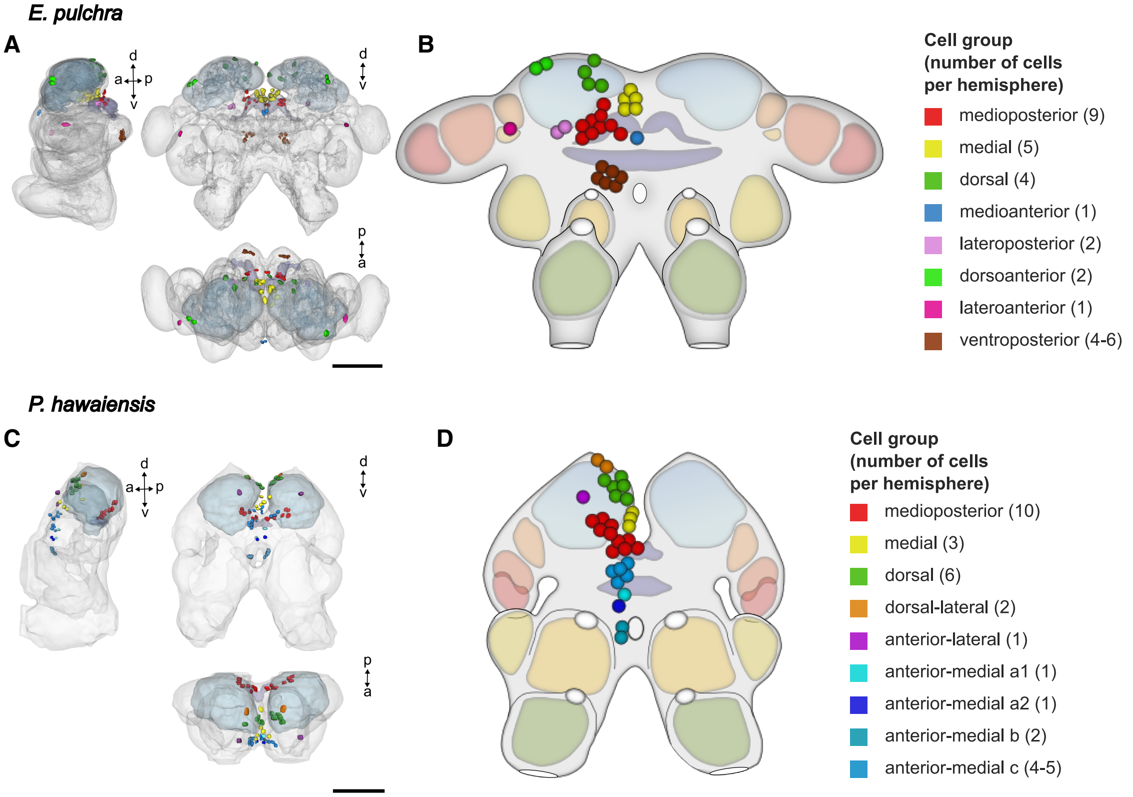

Figure 2. Putative clock cells in the brains of *E. pulchra* and *P. hawaiensis* revealed by co-expression of canonical circadian clock genes

(A) Lateral, frontal, and dorsal views of the *E. pulchra* reference brain model (from Figure S2A) on to which somata enriched for expression of *Epper* and *Epcry2* are registered and segmented. The neuropils used in Figure S2 to signpost putative clock cells are here made translucent.

(B) Diagrammatic representation of the *E. pulchra* brain summarizing the locations of cells co-enriched for *Epper* and *Epcry2*, based on the 3D reconstruction in (A).

(C) Lateral, frontal, and dorsal views of the *P. hawaiensis* reference brain model (from Figure S2C) on to which somata enriched for expression of *Phper* and *Phcry2* are registered and segmented.

The neuropils used in Figure S2 to signpost putative clock cells are here made translucent.

(D) Diagrammatic representation of the *P. hawaiensis* brain summarizing the locations of cells co-enriched for *Phper* and *Phcry2* based on the 3D reconstruction in (C).

Compass markers in (A) and (C) show anterior (a), posterior (p), dorsal (d), and ventral (v) directions. Discrete cell groups are color coded (see key). Cell numbers within each group (per hemisphere) in parenthesis. Scale bars: 100 μm.

See also Figures S1–S6, Table S1, and Videos S1 and S2.

that *per* and *cry2* encode the principal negative regulators of E-box-mediated transcription in *E. pulchra*,[12] this small population of cells in the median protocerebrum co-expressed the full complement of genes required for a functional TTFL, highlighting them as putative clock cells. Using a similar approach in *P. hawaiensis*, we found, again, that *Clk*-enriched cells expressed *Bmal1* and high levels of *cry2* (Figure 1D). Furthermore, the *cry2*-enriched (and hence, *Clk*-enriched) cells also expressed *per* (Figure 1E), indicating that they are likely TTFL-competent cells. By combining HCR-FISH with anti-synapsin (SYNORF1) immunofluorescence (Figure S6; Videos S1 and S2), the putative clock cell clusters were registered to the reference brains (Figure 2). The groups were named by general location: 8 groups in *E. pulchra* and 9 in *P. hawaiensis*, with a total of ~30 cells per hemisphere, distributed symmetrically. Cell groups and their given nomenclature are summarized in Table S1 and their distribution is described in Figure S6. Having defined the neuroanatomical distribution of putative clock cells, we then explored their capacity for rhythmic expression of clock genes and asked whether any of them can track daily or tidal time?

Circadian clock genes are expressed rhythmically in putative clock cells of *E. pulchra* brain

When placed into free-running conditions (DD) in the laboratory, field-collected *E. pulchra* displayed strong circatidal activity rhythms, with peak activity corresponding to the expected time of high water on the home beach (Figure S7). Across 11 independent field collections, 54.5% ± 6.5% of animals were tidally rhythmic (range: 20%–76%), with a mean period of 12.36 ± 0.02 h (±SEM, N = 478). To test whether some or all putative clock cells of *E. pulchra* can track daily or tidal time, we performed whole-mount, multiplexed HCR-FISH on dissected brains of circatidally rhythmic beach-collected *E. pulchra* sampled every 2 h under LD (16L:8D) for 24 h (Figure 3A). The number of HCR-FISH spots was used to quantify transcript abundance, and we considered expression to be rhythmic only

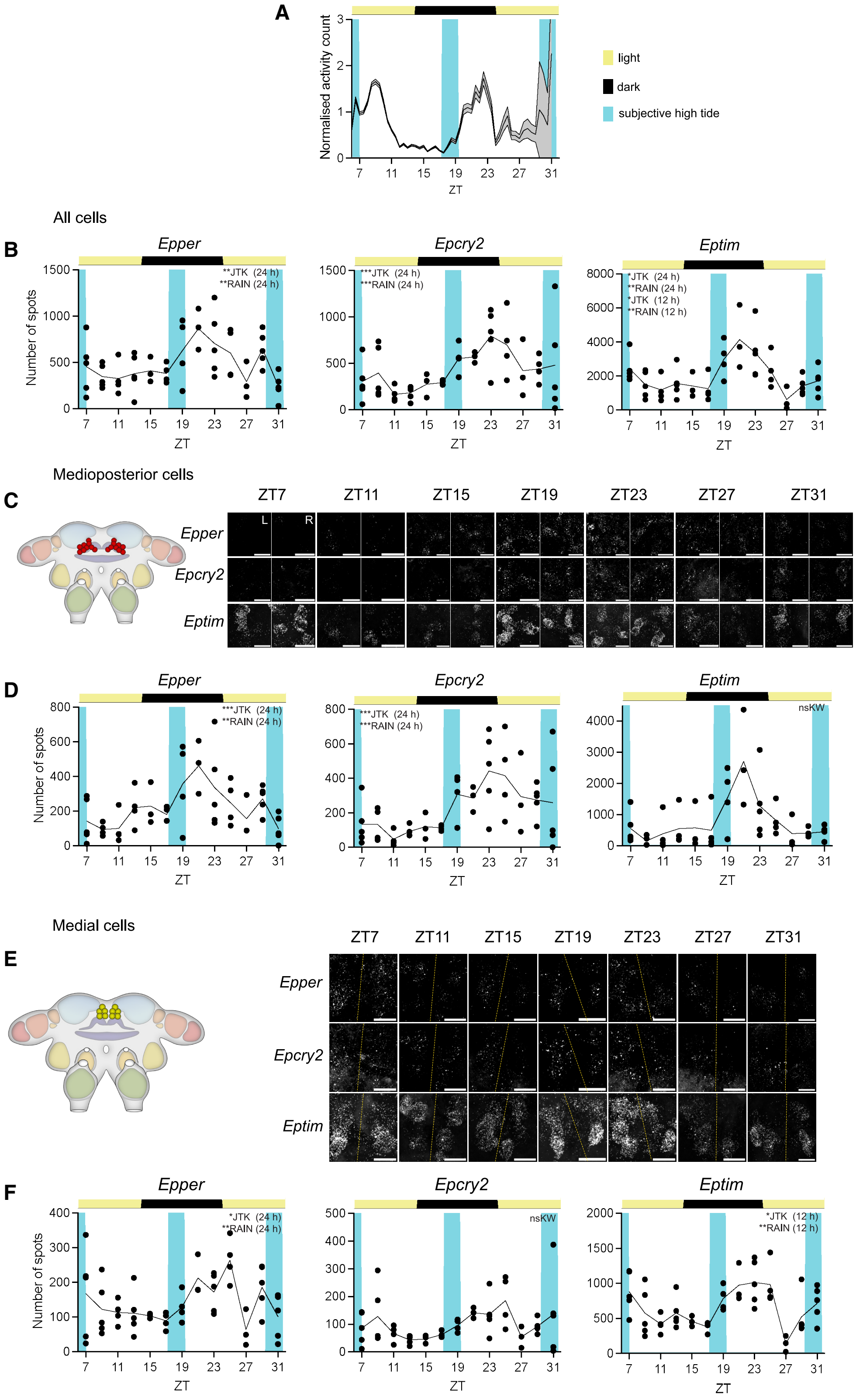

Figure 3. Time course of expression of *per, cry2*, and *tim* in cells of tidally rhythmic *E. pulchra* held on a LD cycle

(A) Normalized swimming activity (mean + SEM, shading) of beach-collected *E. pulchra* harvested under LD (16L:8D) for the HCR-FISH time course (initial group size at start of harvest at ZT7 = 65). See also Figure S7.

(legend continued on next page)

CellPress
OPEN ACCESS

when Kruskal-Wallis/ANOVA identified a significant time effect and both JTK_CYC[20] and RAIN[21] were significant ($p < 0.05$) at a period of 12 or 24 h for circatidal and daily rhythms, respectively. The aggregated counts across all cells, as a measure of total brain expression, showed strong daily rhythms for *per*, *cry2*, and *tim*, with peak levels in the late dark phase (Figure 3B). Aggregate levels of *tim* also showed a statistically significant 12-h rhythm, although the overall profiles for aggregate *per*, *cry2*, and *tim* were similar and most clearly tracked daily time.

To explore whether circatidal and circadian behavioral phenotypes are generated by different cell populations that contain ∼12- and ∼24-h molecular timers, we analyzed clock gene expression at the level of individual cell groups. Of the 7 groups examined, 5 showed significant rhythms of one or multiple transcripts (Table S2). Daily patterning was evident for *per* and *cry2* expression in the medioposterior cells, with peaks in late night (Figures 3C and 3D), whereas medial cells had a daily rhythm of *per* but a 12-h rhythm of *tim* (Figures 3E and 3F). Lateroposterior cells also had a significant 12-h cycle of *tim* (Figure S8), whereas *per* and *cry2* levels in these cells were not significantly rhythmic. In summary, in circatidally behaving *E. pulchra* sampled under LD, aggregate expression of *per* and *cry2* showed a daily rhythm, whereas aggregate levels of *tim* were more complex, reflecting both daily and circatidal time. When examined across distinct cell groups, daily cycles of *per* and *cry2* were evident in 4 of 7 groups, whereas a 12-h, putatively circatidal, pattern of *tim* expression was detected in two. Thus, distinct cell groups in *E. pulchra* appear able to track either daily or tidal time.

Circadian and circatidal expression of clock genes in separate cell groups of *P. hawaiensis* brain

To establish circatidal behavioral rhythms, *P. hawaiensis* laboratory-reared under a 12L:12D cycle were entrained by a cycle of mechanical agitation for 2 h every 12.4 h for >30 days. On transfer to free-running conditions of DD and no further agitation, they showed circatidal rhythms with peak activity phased to anticipated agitation (i.e., subjective high tide) (Figures S9A and S9B). Across 13 experiments, 52.8% ± 2.8% of animals showed circatidal behavior (range 40%–70%) with a mean period of 12.40 ± 0.02 h (±SEM, N = 456) (Figures S9C and S9D). To confirm that the circatidal behavior was phased by the agitation and not by the LD cycle, a sub-group of animals were subjected to a phase-shifted regime for the final 6 days of entrainment in which agitation occurred every 12.9 h rather than every 12.4 h as in matched controls. It therefore accumulated a relative delay of ∼6 h (0.5 h per cycle over ∼12 cycles). On release to DD, control and phase-shifted animals exhibited circatidal behavioral rhythms with peak activity at projected high tide and periods of ∼12.4 h that were not significantly different between groups (Kolmogorov-Smirnov test: D = 0.1779, p = 0.59, n.s.) (Figures S9E–S9G). Over four replications of the control and shifted regimes, their effectiveness (45.7% vs. 49.8% circatidal animals) was comparable (paired t test: t = 0.27, df = 3, p = 0.80, n.s., Cohen's d = 0.136). Importantly, the phase of the circatidal behavior was delayed by 4.94 ± 0.51 h (±SEM, N = 4) in phase-shifted animals relative to the controls (Figures S9E, S9F, and S9H). This not only confirms the capacity of *P. hawaiensis* to exhibit circatidal behavior[13] but also demonstrates that the circatidal clockwork of *P. hawaiensis* is sensitive to mechanical agitation, as is the case for *E. pulchra*.[11,12]

To test whether cells in *P. hawaiensis* could track circadian and/or tidal time, whole-mount HCR-FISH was applied to the brains of animals entrained by tidal agitation and exhibiting circatidal activity before harvest (Figure 4A). Aggregate expression of *per* was circadian, peaking in subjective night, whereas aggregate expression of *cry2* did not vary with time (Figure 4B). Across the 10 cell groups examined, 3 groups showed significant rhythms of one or multiple transcripts (Table S3). Notably, expression of *per* was strongly circadian in medioposterior cells and anterior-medial a1 cells (Figures 4C–4F). Moreover, these cells also showed circadian cycles of *cry2* expression, running in antiphase to *per*. Thus, *per* and *cry2* expression in these cells tracked CT rather than tidal time in circatidally rhythmic animals. In contrast, dorsal-lateral cells in the same animals showed a significant 12-h cycle of *per* and *cry2* transcripts (Figures 4G and 4H). Furthermore, in the dorsal-lateral cells, unlike the circadian cells, *per* and *cry2* oscillated almost in phase, both peaking after subjective high tides (∼CT00-03 and CT15).

The experiment was repeated, with the final tidal agitation occurring slightly later (+1.2 h) relative to the LD cycle. The animals again exhibited robust circatidal activity rhythms in DD, phased to anticipated high water (Figure 5A). Aggregate expression of *per* was circadian (Figure S10A) and, across the 10 cell groups examined, the same three groups (medioposterior, anterior-medial a1, and dorsal-lateral) showed significant rhythms of one or multiple transcripts (Table S4). The expression of *per* and *cry2* in the medioposterior and anterior-medial a1 cells was circadian (Figures S10B–S10E), whereas the dorsal-lateral cells again showed a strong circatidal, 12-h pattern of expression of *per* and *cry2*. The transcripts were also oscillating in phase and peaking after subjective high tide (Figures 5B, 5C, and S10F). To test whether we could phase-shift these putative tidal cells even further, and to differentiate them from light-entrained

(B) Mean aggregate transcript abundance, quantified as the number of FISH spots, of *Epper*, *Epcry2*, and *Eptim* across all cells plotted across time in beach-collected animals held under LD.
(C) Left: cartoon to show location of medioposterior cell group in the brain. Right: representative maximum intensity Z-projections of *Epper*, *Epcry2*, and *Eptim* expression in both hemispheres (L: left, R: right) across ZT (individual HCR-FISH channels in rows). Scale bars: 20 μm.
(D) Mean transcript abundance, quantified as the number of FISH spots, of *Epper* and *Epcry2* in medioposterior cell group plotted across time in beach-collected animals held under LD.
(E and F) As (C) and (D) but for medial cells group. Yellow dashed lines: midlines. Scale bars: 10 μm.
Each point is from one brain, total across hemispheres. n = 3–5, N = 55. Inset text indicates statistical tests (JTK-cycle and RAIN) performed to determine 24- and 12-h rhythmicity, with the tested periods in parentheses, following significant time effect by ANOVA or Kruskal-Wallis. ***$p < 0.001$, **$p < 0.01$, *$p \leq 0.05$, $^{ns}p > 0.05$. See also Figure S8 for clock gene expression in lateroposterior cell group. For statistics and data shown in this figure, refer to Table S2. See also Figure S15 and Table S10.

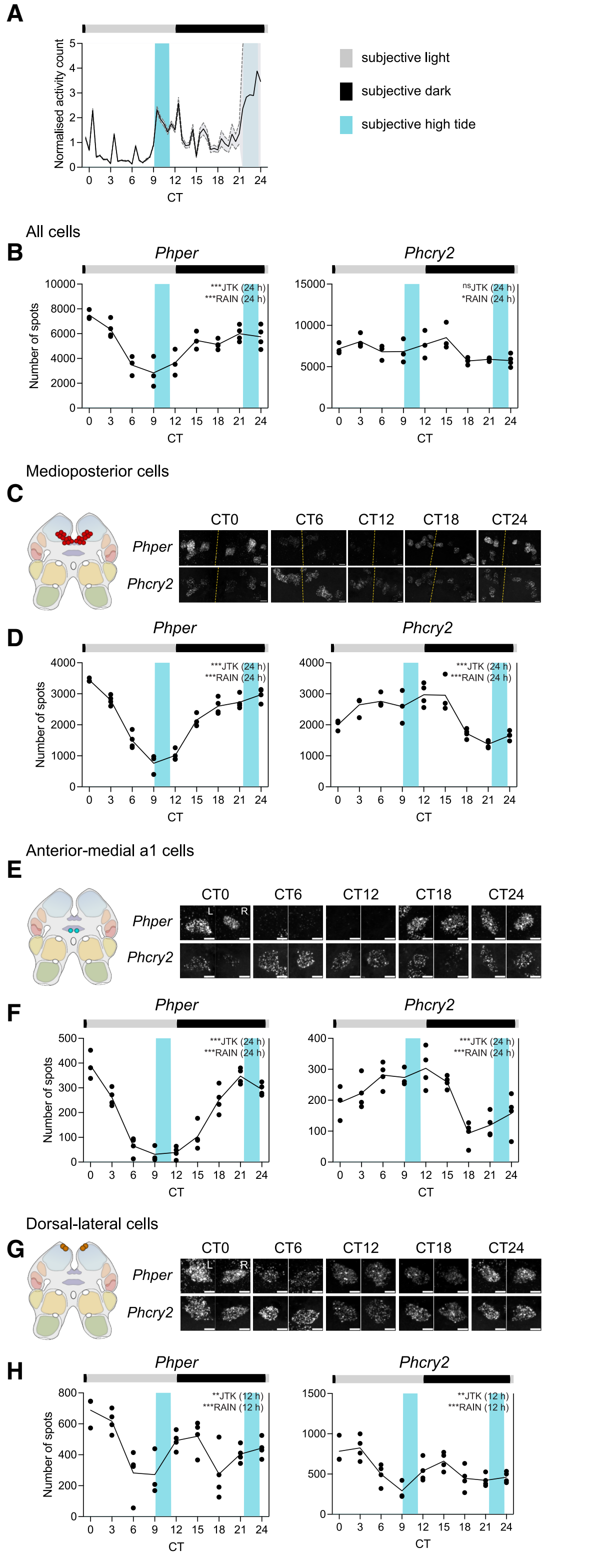

Figure 4. Free-running clock gene expression rhythms in tidally entrained *P. hawaiensis* (tidal phase θ)

(A) Normalized swimming activity (mean + SEM, shading) of *P. hawaiensis* synchronized to the prior agitation cycle and harvested for the HCR-FISH time course (initial group size at start of harvest at CT0 = 37).

(B) Mean aggregate transcript abundance, quantified as the number of FISH spots, of *Phper* and *Phcry2* across all putative clock cells plotted across time in tidally entrained animals sampled under DD. $n = 3–4$, $N = 31$.

(C) Left: cartoon to show location of medioposterior cell group in the brain. Right: representative maximum intensity Z-projections of *Phper* and *Phcry2* expression in both hemispheres (yellow dashed lines: midlines) across CT (individual HCR-FISH channels in rows).

(D) Plots of mean transcript abundance, quantified as the number of FISH spots, across CT for *Phper* (left) and *Phcry2* (right). $n = 3–4$, $N = 33$.

(E and F) As in (C) and (D) for anterior-medial a1 cells. $n = 3–4$, $N = 34$. L, left; R, right.

(G and H) As in (C) and (D) for dorsal-lateral cells. $n = 3–4$, $N = 34$. L, left; R, right.

Each point is from one brain, total across hemispheres. Inset text indicates statistical tests (JTK-cycle and RAIN) performed to determine 24- and 12-h rhythmicity, with the tested periods in parentheses, following significant time effect by ANOVA or Kruskal-Wallis. $^{***}p < 0.001$, $^{**}p < 0.01$, $^{*}p \leq 0.05$, $^{ns}p > 0.05$. For statistics shown in this figure, refer to Table S3. Scale bars: 10 μm (C), 5 μm (E and G). See also Figures S9 and S15 and Tables S7 and S10.

circadian cells exhibiting bimodal 24-h rhythms, we entrained animals such that the final bout of tidal agitation occurred 5.4 h phase-shifted relative to the 12:12LD cycle (i.e., subjective high tides at ∼CT3.8 and ∼CT16.2). On release into constant conditions, the animals exhibited appropriately phased circatidal rhythms of activity peaking at subjective high tide (Figure 5A). The aggregate expression of *per* (but not *cry2*) was circadian, peaking in subjective night (Figure S11A). Of the 10 cell groups examined, 4 (the original three plus anterior-lateral cells) showed an effect of time on transcript levels (Table S5). Medioposterior cells showed antiphasic circadian rhythms of *per* and *cry2*, anterior-medial a1 cells showed a circadian cycle of *per* (Figures S11B–S11E), and anterior-lateral cells were circadian for *cry2*. In contrast, the dorsal-lateral cells again showed 12-h cycles of expression of *per* and *cry2* (Figures 5D, 5E, and S11F). Moreover, the peaks occurred after subjective high tide and, therefore, the profiles were phase-shifted relative to the prior LD cycle by ∼6 h when compared with the previous tidal experiments. Comparison across the three experiments with circatidally active animals showed conservation of the phase relationship between *per* and *cry2* expression across studies as well as adoption of the phase of the prior regime of tidal agitation (Figure S12). Thus, in the same brains of *P. hawaiensis* exhibiting free-running circatidal behavior, medioposterior and anterior-medial a1 cells consistently tracked CT, expressed by antiphasic rhythms of *per* and *cry2* expression, whereas the dorsal-lateral cells consistently tracked tidal time, independently of CT, and did so with *per* and *cry2* rhythms in phase.

Loss of circatidal but not daily expression of clock genes in putative clock cells of *P. hawaiensis* under a LD cycle

To test whether dorsal-lateral cells have any capacity to follow daily time, we entrained *P. hawaiensis* to LD cycles without tidal agitation. As reported,[10,13] *P. hawaiensis* exhibited rhythmic activity under LD, peaking at night (Figures S13A, S13B, S13D, and S13E). Across 7 experiments 58.6% ± 4.4% were rhythmic (range: 45%–80%, total $N = 189$). When released into constant darkness, ∼50% of rhythmic animals (i.e., 22.9% ± 7.5% of total) showed circadian rhythms in their locomotor activity (Figures S13A, S13C, S13D, and S13F), with a mean (±SEM) period of 24.17 ± 0.17 h ($N = 39$). Activity levels were significantly higher during the subjective night than in the subjective day.

CellPress
OPEN ACCESS

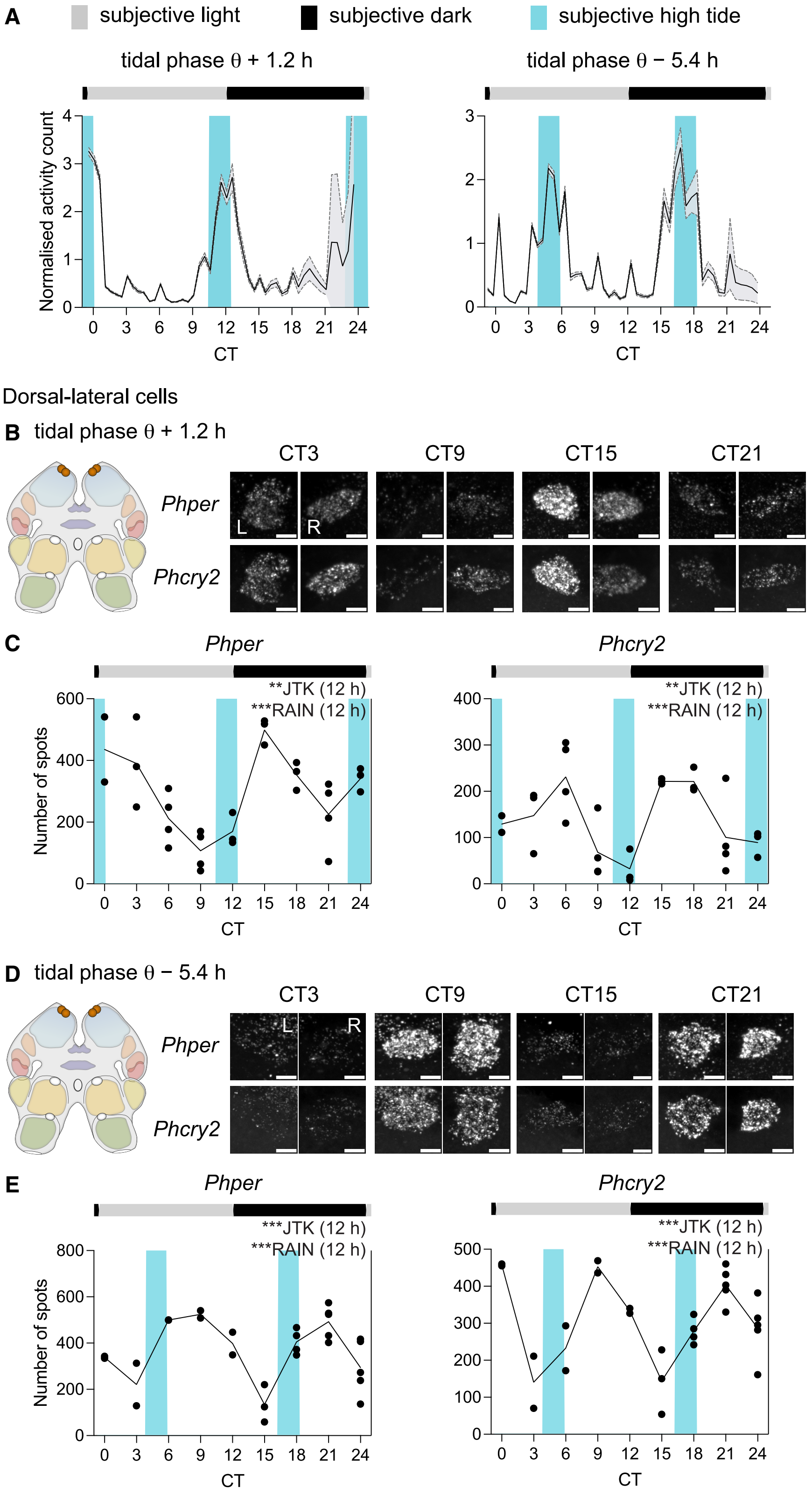

Figure 5. Circatidal rhythms of expression of *Phper* and *Phcry2* in dorsal-lateral cells in the brain of tidally entrained *P. hawaiensis*

(A) Normalized swimming activity (mean + SEM, shading) of *P. hawaiensis* synchronized to the prior agitation cycle, phase-shifted by +1.2 h (left) or −5.4 h (right) and harvested for the HCR-FISH time course (initial group sizes at start of harvest at CT0 = 35).

(legend continued on next page)

In animals confirmed to be showing daily behavioral activity under 12L:12D prior to harvest of their brains for HCR-FISH (Figure 6A), the aggregate expression of *per* was highly rhythmic, peaking at night (Figure 6B). Aggregate expression of *cry2* was also significantly rhythmic, in antiphase to *per*, but with a small amplitude. Of the 10 cell groups examined, 5 groups showed a daily pattern of one or both transcripts (Table S6). In both the medioposterior cell group and the anterior-medial a1 cells, *per* and *cry2* were rhythmic and in antiphase, with nocturnal and diurnal peaks, respectively (Figures 6C–6F), echoing the circadian patterns seen in these cells in tidally active animals under DD. Rhythmic expression of *per* was also evident in the dorsal cells and the anterior-medial b cells (Figures S14A–S14D), again peaking in the night, and *cry2* was rhythmic in anterior-medial c cells. In contrast, there was no statistically significant rhythmic expression of either *per* or *cry2* in the dorsal-lateral cells of the daily-behaving animals (Figures 6G and 6H). Thus, several cell groups can exhibit daily cycles of gene expression in daily-behaving *P. hawaiensis* under LD, including the medioposterior and anterior-medial a1 cells that are also circadian under DD. In contrast, the circatidal gene expression observed in dorsal-lateral cells in tidally rhythmic animals under DD was specific to that condition: in the absence of tidal cues this population of cells did not show tidal patterning nor did they adopt the daily rhythms of expression seen in other cell groups under LD.

The distinctions between circadian and circatidal rhythms of gene expression are evident in a combined meta-analysis across the three tidal entrainment studies, registered alternatively in either circadian or circatidal time (Figure 7). When examined across all experiments in the same circatidally behaving animals, the expression of *per* and *cry2* was highly circadian in the medioposterior cells and anterior-medial a1 cells (Figures 7A and 7B), with *cry2* oscillating in antiphase to *per*. On the other hand, the expression of *per* and *cry2* in dorsal-lateral cells was highly circatidal and co-phasic (Figure 7C). Circular plots registered to prior LD and tidal agitation emphasize these differences in periodicity and acrophase between the circadian and the circatidal cells (Figures 7D and 7E). Moreover, this mapping of the tidal oscillations to the phase of tidal entrainment in all three experiments confirmed that the dorsal-lateral cells were able to express a free-running molecular signature of tidal time that was independent of CT and revealed, thereby, that the brain of *P. hawaiensis* contains independent circadian and circatidally rhythmic cell populations.

DISCUSSION

Examination of the expression of canonical circadian clock genes across the brains of both *E. pulchra* and *P. hawaiensis* identified ∼60 cells co-expressing *Clk*, *Bmal1*, *cry2*, and *per* (and *tim* in *E. pulchra*), arranged into distinct groups across the protocerebrum. Although the loss of *Bmal1* compromises circatidal behavior in *E. pulchra*[16] and in *P. hawaiensis*,[13] its brain-wide expression showed no local enrichment and so did not highlight any potential circatidal clock cells. This broad distribution is, however, consistent with BMAL1 having diverse, pleiotropic actions beyond timekeeping.[22] On the other hand, the observed enrichment of *Clk*, *per*, and *cry2* expression (alongside *tim* in *E. pulchra*) in specific cell groups indicated their potential function as circadian oscillators.

In circatidally active *E. pulchra* sampled under LD cycles, the aggregated signals for *per* and *cry2* were daily, reflective of their putative circadian roles, and both peaked in the late night, as expected for negative co-regulators within the TTFL. In contrast, aggregate brain expression of *tim* showed significant patterning not only at 24 h, consistent with circadian cycling of *tim* in whole heads assayed by qPCR,[12] but also at 12 h. Analysis at the level of individual cell groups provided more granular insight, revealing 24-h rhythms of *per* and *cry2* in medioposterior cells and of *per* in medial cells, with peak expression in late night. This absence of circatidal rhythmicity of *per* expression resonates with the finding that knockdown of *per* to ∼20% of wild-type levels, had no effect on circatidal activity in *E. pulchra*, although it did disrupt circadian phenotypes.[12] Alternatively, *tim* showed 12-h accumulation in both the medial and the lateroposterior cells, peaking ∼2 h after expected high water. These cell-specific differences in *tim* expression likely underlie the compound periodicity evident in the aggregate expression. Furthermore, the 12-h patterns in the medial and the lateroposterior cells indicate potential tidal timekeeping in these cells associated with circatidal behavior.

In *P. hawaiensis*, aggregate expression of *per* tracked daily time under LD and CT under DD, with elevated levels during the dark phase and subjective night, respectively. Daily and circadian patterning were even more pronounced when examined in individual cell groups, with rhythmic expression of *per* evident in medioposterior, anterior-medial a1, dorsal, and anterior-medial b cells. Notably, whereas *per* and *cry2* were in phase in daily cells of *E. pulchra*, expression of *per* and *cry2* in daily and circadian cells in *P. hawaiensis* was antiphasic, suggesting an inter-species difference in the underlying molecular architecture of the circadian TTFL. This suggests that *cry2* and *per* may carry different circadian regulatory sequences to direct their specific phases, and future analyses of the *P. hawaiensis* and *E. pulchra* genomes may illuminate this mechanism. It also raises questions about protein levels; for example, are PER and CRY2 expressed in antiphase? A precedent comes from work in the mouse SCN, where the levels of mPER2 and mCRY1 proteins

(B) Left: cartoon to show location of dorsal-lateral cell group in the brain. Right: representative maximum intensity Z-projections of *Phper* and *Phcry2* in both hemispheres (L, left; R, right) across CT (individual HCR-FISH channels in rows). Scale bars: 5 μm.
(C) Plots of mean transcript abundance, quantified as the number of FISH spots, across CT for *Phper* (left) and *Phcry2* (right) (blue shadings: subjective high water). n = 2–4, N = 29.
(D and E) As in (B) and (C) for animals entrained to a tidal cycle advanced, relative to the LD cycle, by 5.4 h. n = 2–5, N = 27.
Each point is from one brain, total across hemispheres. Inset text indicates statistical tests (JTK-cycle and RAIN) performed to determine 24- and 12-h rhythmicity, with the tested periods in parentheses, following significant time effect by ANOVA or Kruskal-Wallis. ***$p < 0.001$, **$p < 0.01$, *$p \leq 0.05$, $^{ns}p > 0.05$. For statistics and data of FISH time course experiments on tidally entrained animals, refer to Tables S4 and S5 for tidal phases $\theta + 1.2$ and $\theta - 5.4$ h, respectively. See also Figures S10, S11, S12, and S15 and Tables S7 and S10.

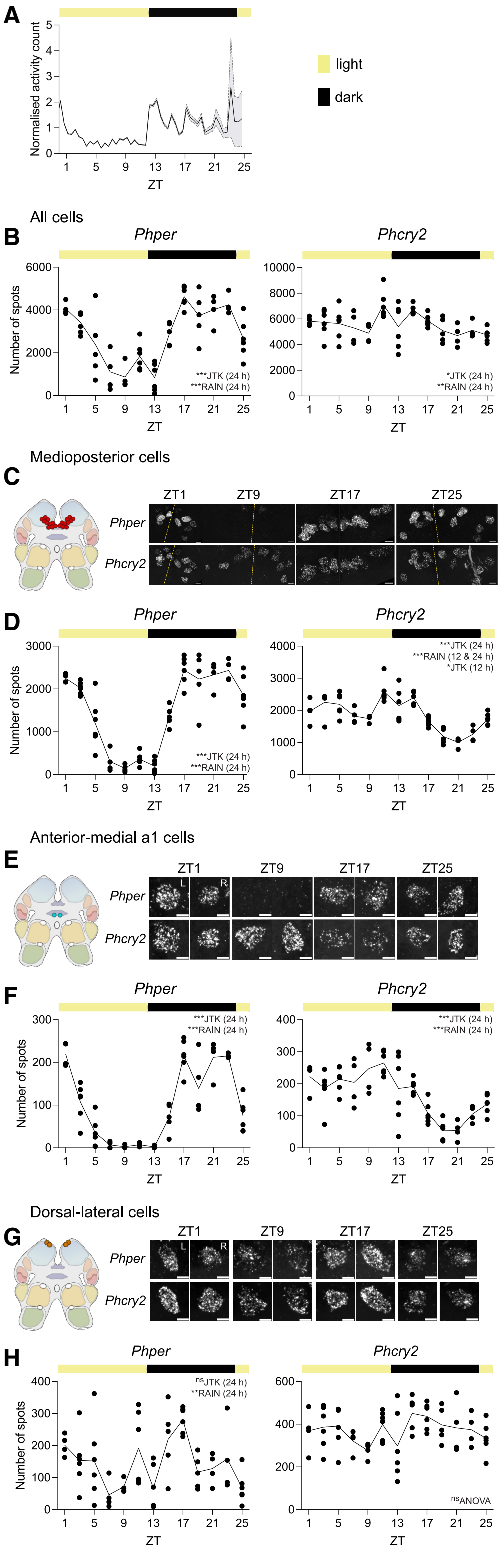

Figure 6. Time course of expression of *per* and *cry2* in cells of *P. hawaiensis* synchronized to the LD cycle

(A) Normalized swimming activity (mean + SEM, shading) of *P. hawaiensis* synchronized to the LD cycle and harvested for the HCR-FISH time course (initial group size at start of harvest at ZT1 = 79).

(B) Mean aggregate transcript abundance, quantified as the number of FISH spots, of *Phper* and *Phcry2* across all cells plotted across time in animals entrained to, and sampled under, a 12 h:12 h LD cycle. n = 4–6, N = 66.

(C) Left: cartoon to show location of medioposterior cell group in the brain. Right: representative maximum intensity Z-projections of *Phper* and *Phcry2* expression in both hemispheres (yellow dashed lines: midlines) across ZT (individual HCR-FISH channels in rows).

(D) Plots of mean transcript abundance, quantified as the number of FISH spots, across daily time for *Phper* (left) and *Phcry2* (right). n = 4–6, N = 66.

(E and F) As for (C) and (D) for anterior-medial 1a cells. n = 4–6, N = 66. L, left; R, right.

(G and H) As for (C) and (D) for dorsal-lateral cells. n = 4–6, N = 66.

Each point is from one brain, totaled across hemispheres. Inset text indicates statistical tests (JTK-cycle and RAIN) performed to determine 24- and 12-h rhythmicity, with the tested periods in parentheses, following significant time effect by ANOVA or Kruskal-Wallis. $^{***}p$ < 0.001, $^{**}p$ < 0.01, $^{*}p \leq 0.05$, ^{ns}p > 0.05. See also Figure S14 for other cell groups with 24-h cycling in clock gene expression. For statistics and data shown in this figure, refer to Table S6. Scale bars: 10 μm (C), 5 μm (E and G). See also Figures S13 and S15 and Table S10.

are temporally segregated, with mPER2 peaking ∼6 h before peak mCRY1.[23]

Remarkably, following entrainment of *P. hawaiensis* to artificial tidal cycles under LD and then subsequent transfer to free-running conditions, *per* and *cry2* expression in dorsal-lateral cells was robustly rhythmic, with a circatidal period that reflected the circatidal behavior of the animals. Across independent trials, these cells tracked experimental tidal time, regardless of prior LD phase. In contrast, clock gene expression in these cells was arrhythmic when analyzed across individuals sampled under LD without tidal cues and with daily, but not tidal, behavior. These observations place the dorsal-lateral cells as strong candidates for dedicated circatidal oscillators, especially when other cell groups in the same brains (e.g., medioposterior and anterior-medial a1 cells) ignored tidal cues and remained circadian, phased to prior LD. Notably, *per* and *cry2* in these tidal cells oscillated in phase, in contrast to the antiphasic rhythms of *per* and *cry2* in daily/circadian cells. The functional significance of these varying relationships, which are surprising in terms of the canonical TTFL whereby *per* and *cry2* are typically co-phasic, is not clear, but they suggest that a common molecular architecture may have diverged, evolving to operate under different timebases in different cells. Of the canonical clock genes in *E. pulchra*, only *tim* expression was associated with tidal time in 2 of the 4 neuronal groups studied. Future analysis of protein expression in daily and tidal cell groups will better inform understanding of cell-specific TTFL structures. In addition, examination of clock gene expression in the different cell groups of animals with genetic deletion or knockdown of BMAL1 (and CLK) would indicate mechanistic bridges between the TTFL and tidal behavior. It is possible that the circatidal expression patterns of *Phper*, *Phcry2*, and *Eptim* are driven by feedback from a cryptic tidal clock that exists within, or even outside, those cells. Consequently, these patterns may reflect cellular activity but are not in themselves part of the clock nor are they causal to BMAL1-dependent tidal behavior. Such a scenario could explain why RNAi of canonical negative regulators had no effect on tidal behavior in *E. pulchra*.[12,16] The genetic tractability of *P. hawaiensis* may enable this to be tested directly.

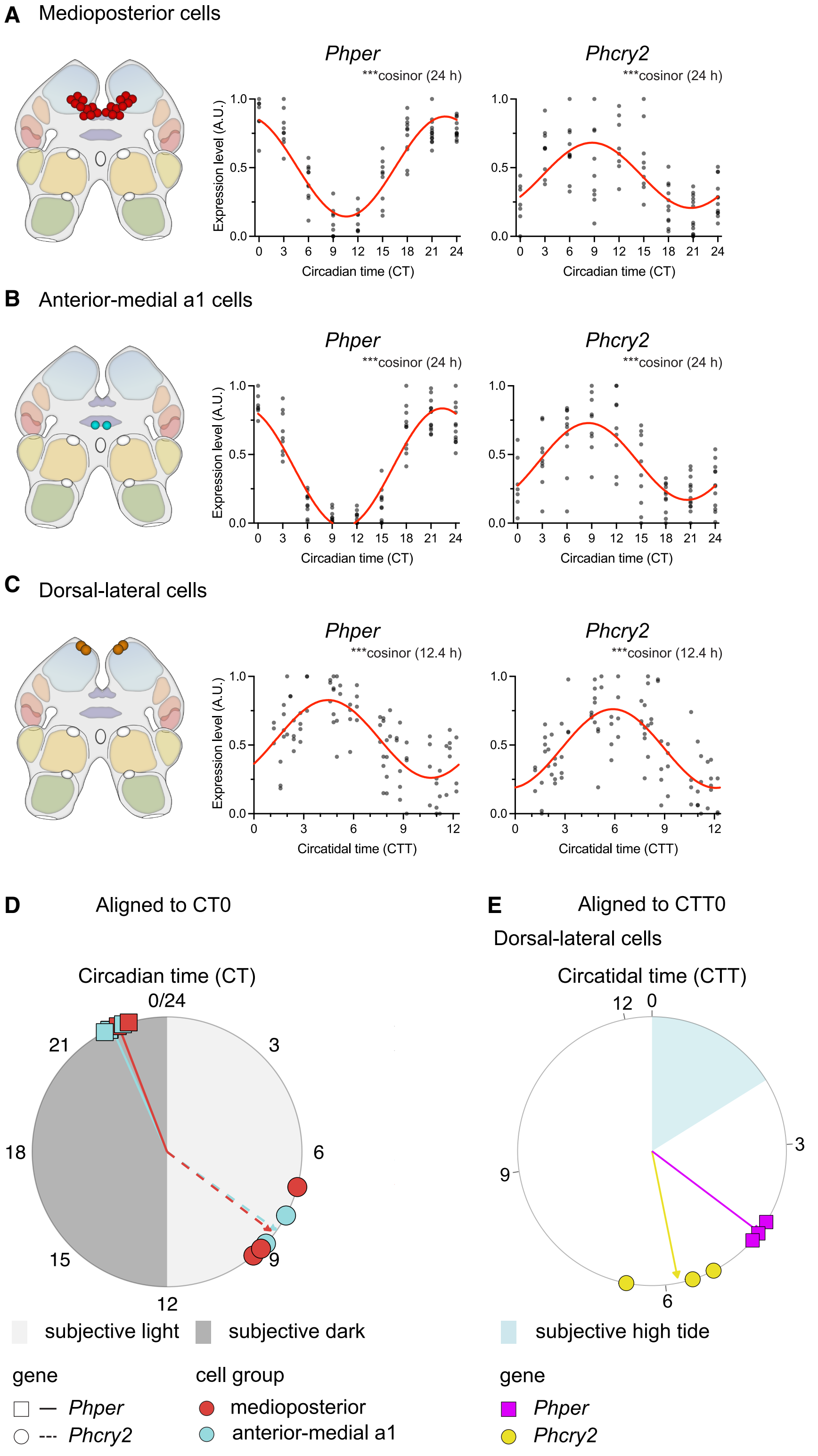

Figure 7. Summary meta-analysis of circatidal and circadian rhythms of clock gene expression in medioposterior, anterior-medial a1, and dorsal-lateral cell groups of *P. hawaiensis*

(A) Left: cartoon to show location of the medioposterior cell group in the brain. Right: scatterplot of expression of *Phper* (left) and *Phcry2* (right) across three independent experiments registered to subjective CT (CT = 0). Red lines indicate 24-h cosinor fits.

(B) As in (A) for anterior-medial a 1 cell group.

(C) As in (A) for dorsal-lateral cell group, but plots are registered to subjective high tide (circatidal time [CTT] = 0). Red lines indicate 12.4-h cosinor fits. Inset text in (A)–(C) indicate statistical significance of cosinor.

(D) Circular plot of circadian peak expression phase of *Phper* and *Phcry2* in selected rhythmic cell groups of *P. hawaiensis* exhibiting circatidal activity rhythms. Each color-shape combination represents data from across *N* = 3 experiments. Arrowed lines indicate mean phase for each gene-cell group combination, and length of arrowed lines indicates the power of the vector at each mean.

(E) Circular plots of circatidal peak expression phase of *Phper* and *Phcry2* in the dorsal-lateral cells of *P. hawaiensis* exhibiting circatidal activity rhythms, with the circatidal acrophase aligned to CTT0 within each individual experiment. Arrowed lines indicate mean phase for each gene-cell group combination, and length of arrowed lines indicates the power of the vector at each mean. For mean phase and mean vector lengths for each gene-cell group combination in (D) and (E), see Table S7.

Notably, even though numerous cells oscillated at ~24 h (22 in *P. hawaiensis*) and only a few at ~12 h (4 in *P. hawaiensis*), both *E. pulchra* and tidally entrained *P. hawaiensis* were strongly circatidal in terms of behavior. This raises the question of why strong tidal phenotypes are reflected by relatively few tidal cells compared with the strong aggregate cycles of daily/circadian gene expression. Of course, day/night cycles are highly predictable in nature, and it is therefore expected that circadian systems will represent a prominent and robust feature. Nevertheless, coastal animals have evolved temporally complex behaviors to match their habitats, cued by multiple stimuli.[10–13] It is therefore possible that gene expression may be circatidal in additional cell groups in *E. pulchra* and/or *P. hawaiensis*, but it evaded statistical detection in the current study because of the high variance intrinsic to population-based measures. Alternatively, different tidal cell groups may respond to distinct entraining cues and so only a subset of these groups is revealed in any single experimental setting. Examination of gene expression in the brain of *P. hawaiensis* entrained by tidal immersion/emersion,[13] with and without tidal agitation, may indeed reveal additional putative circatidal clock cells: can subsets of cells we describe here as

CellPress
OPEN ACCESS

non-rhythmic be recruited by tidal stimuli of other modalities? Alternatively, is there a unique tidal role for dorsal-lateral cells? Such a cell- and stimulus-specific entrainment repertoire raises the question of how tidal stimuli are transduced to the central brain and whether a distributed multi-modal network broadens the capacity for tidal entrainment, reinforcing it in diverse natural settings.[2,10,11]

In considering the three hypotheses of circatidal timekeeping,[4,6,24] our data are most consistent with the model of dedicated tidal and daily timers proposed by Naylor,[6] with canonical circadian genes in distinct cell groups tuning to either circatidal or circadian periodicities. Our demonstration that dual circatidal and circadian systems operate in parallel echoes other work on circatidal clocks. For example, surgical removal of the optic lobes of the mangrove cricket, *Apteronemobius asahinai*, abrogates circadian but not circatidal locomotor phenotypes, indicative of two anatomically separable timers.[25,26] In contrast, evidence in favor of a circadian/tidal timer based on a single "plastic" clock, as proposed by Enright,[4] received support from studies on the coastal oyster *Crassostrea gigas*, which showed ∼12-h and/or ∼24-h expression of circadian gene homologs.[27] Importantly, these models are not mutually exclusive. Given the evolutionary distance between mollusks and arthropods, it is entirely possible that tidal timekeeping machineries have evolved independently. In fact, a parsimonious explanation for the evolution of clock cells with different periodicities would be that pre-existing circadian clock components were co-opted to track relevant tidal cues. The cells we identified invariably co-expressed the rudiments of a circadian TTFL but with either circadian or circatidal frequencies in the same brain, and it is possible that they evolved in parallel, diverging under the influence of daily (e.g., photosensory) and tidal (e.g., mechanosenory) inputs, respectively.

Kwiatkowski et al.[10] asked how *P. hawaiensis* "chooses" between 12.4- and 24-h environmental rhythms during entrainment. Our observation that distinct circadian and circatidal cell groups entrain independently to light and mechanical stimuli, respectively, provides a solution to this problem. Similarly, these authors also questioned whether neurons coordinating rhythmic behavior in *P. hawaiensis* have two different timers, with differential sensitivity to light and tides, or whether two cell types exist. Although we cannot currently exclude the former, our results align better with the latter. This does not preclude interactions of the two systems, however, and the genetic and behavioral malleability of *P. hawaiensis* should enable their decipherment. Thus, our findings may facilitate the examination of input and output pathways of tidal and daily cues at cellular resolution, progressing toward defining the nature and connectivity of circadian and circatidal neural clockworks. In conclusion, our results implicate canonical circadian mechanisms in circatidal timekeeping and reveal brain networks that may represent neural substrates for the generation of interactive daily and tidal rhythms.

RESOURCE AVAILABILITY

Lead contact

Requests for further information, resources and code should be directed to, and will be fulfilled by, the lead contact, Michael H. Hastings (mha@mrc-lmb.cam.ac.uk).

Materials availability

This study did not generate new unique reagents.

Data and code availability

All original code has been deposited at https://github.com/HastingsLab-LMB/fish_pipeline and is publicly available at https://figshare.com/projects/2025_crustacean_clock_cell_rhythms/240869 as of the date of publication. HCR-FISH and behavioral data reported in this paper will be shared by the lead contact upon request.

Any additional information required to reanalyze the data reported in this paper is available from the lead contact upon request.

ACKNOWLEDGMENTS

We thank Dr. Sebastian Cachero (LMB) and Dr. Gregory Jefferis (LMB) for their guidance on reference brain generation and their algorithms, as well as Dr. Aziz Aboobaker (Oxford) for providing a starting culture of Chicago-F *P. hawaiensis* and Dr. Michalis Averoff (Lyon) for advice on working with *P. hawaiensis*. We also thank the Mechanical and Electronics Workshops and the Light Microscopy Facility at the LMB for technical support. This work was supported by core funding from Medical Research Council (MRC) as part of United Kingdom Research and Innovation (also known as UK Research and Innovation) (MRC file reference no. MC_U105170643) to M.H.H. C.Y.S. was supported by a Boehringer Ingelheim Fonds PhD fellowship (2023–2025). For the purpose of open access, the author has applied a CC BY public copyright license to any author accepted manuscript version arising.

AUTHOR CONTRIBUTIONS

Conceptualization, all authors; data curation, A.O., C.Y.S., and M.H.H.; formal analysis, A.O. and C.Y.S.; funding, M.H.H.; investigation, A.O., C.Y.S., and D.C.W.; methodology, A.O., C.Y.S., D.C.W., and M.H.H.; project administration, M.H.H.; software, A.O. and C.Y.S.; resources, A.O., C.Y.S., D.C.W., and M.H.H.; supervision, M.H.H.; validation, all authors; visualization, all authors; writing – original draft, all authors; and writing – review and editing, all authors.

DECLARATION OF INTERESTS

The authors declare no competing interests.

STAR★METHODS

Detailed methods are provided in the online version of this paper and include the following:

- KEY RESOURCES TABLE
- EXPERIMENTAL MODEL AND STUDY PARTICIPANT DETAILS
  - *Eurydice pulchra* collection
  - *Parhyale hawaiensis* husbandry
- METHOD DETAILS
  - *Eurydice pulchra* behavior analysis
  - *Parhyale hawaiensis* behavior analysis
  - Brain collection and processing
  - Immunohistochemistry
  - Reference brains
  - HCR™ RNA-FISH
  - Co-immuno-RNA-FISH
  - RNAscope methods for formalin-fixed, paraffin-wax embedded *E. pulchra* brains
  - Time-course sampling design
  - Whole brain digital HCR-FISH
- QUANTIFICATION AND STATISTICAL ANALYSIS

SUPPLEMENTAL INFORMATION

Supplemental information can be found online at https://doi.org/10.1016/j.cub.2025.04.047.

Received: February 5, 2025
Revised: March 19, 2025
Accepted: April 17, 2025
Published: May 8, 2025

REFERENCES

1. Dunlap, J.C., Loros, J.J., and Decoursey, P.J. (2004). Chronobiology: Biological Timekeeping (Sinauer).
2. Naylor, E. (2010). Chronobiology of Marine Organisms (Cambridge University Press). https://doi.org/10.1017/CBO9780511803567.
3. Häfker, N.S., Andreatta, G., Manzotti, A., Falciatore, A., Raible, F., and Tessmar-Raible, K. (2023). Rhythms and Clocks in Marine Organisms. Ann. Rev. Mar. Sci. *15*, 509–538. https://doi.org/10.1146/annurev-marine-030422-113038.
4. Enright, J.T. (1976). Plasticity in an isopod's clockworks: shaking shapes form and affects phase and frequency. J. Comp. Physiol. *107*, 13–37. https://doi.org/10.1007/BF00663916.
5. Palmer, J.D. (1995). Review of the dual-clock control of tidal rhythms and the hypothesis that the same clock governs both circatidal and circadian rhythms. Chronobiol. Int. *12*, 299–310. https://doi.org/10.3109/07420529509057279.
6. Naylor, E. (1996). Crab clockwork: the case for interactive circatidal and circadian oscillators controlling rhythmic locomotor activity of *Carcinus maenas*. Chronobiol. Int. *13*, 153–161. https://doi.org/10.3109/07420529609012649.
7. Reppert, S.M., and Weaver, D.R. (2000). Comparing Clockworks: Mouse versus Fly. J. Biol. Rhythms *15*, 357–364. https://doi.org/10.1177/074873000129001459.
8. Andreani, T.S., Itoh, T.Q., Yildirim, E., Hwangbo, D.S., and Allada, R. (2015). Genetics of Circadian Rhythms. Sleep Med. Clin. *10*, 413–421. https://doi.org/10.1016/j.jsmc.2015.08.007.
9. Hardin, P.E., and Panda, S. (2013). Circadian timekeeping and output mechanisms in animals. Curr. Opin. Neurobiol. *23*, 724–731. https://doi.org/10.1016/j.conb.2013.02.018.
10. Kwiatkowski, E.R., Rosenthal, J.J.C., and Emery, P. (2024). Crosstalk between the circatidal and circadian clocks mediates behavioral adaptation to tidal patterns. Curr. Biol. *34*, 5239–5251.e2. https://doi.org/10.1016/j.cub.2024.09.067.
11. Hastings, M.H. (1981). The Entraining Effect of Turbulence on the Circa-Tidal Activity Rhythm and Its Semi-Lunar Modulation in *Eurydice pulchra*. J. Mar. Biol. Assoc. UK *61*, 151–160. https://doi.org/10.1017/S0025315400045987.
12. Zhang, L., Hastings, M.H., Green, E.W., Tauber, E., Sladek, M., Webster, S.G., Kyriacou, C.P., and Wilcockson, D.C. (2013). Dissociation of circadian and circatidal timekeeping in the marine crustacean *Eurydice pulchra*. Curr. Biol. *23*, 1863–1873. https://doi.org/10.1016/j.cub.2013.08.038.
13. Kwiatkowski, E.R., Schnytzer, Y., Rosenthal, J.J.C., and Emery, P. (2023). Behavioral circatidal rhythms require Bmal1 in *Parhyale hawaiensis*. Curr. Biol. *33*, 1867–1882.e5. https://doi.org/10.1016/j.cub.2023.03.015.
14. O'Neill, J.S., Lee, K.D., Zhang, L., Feeney, K., Webster, S.G., Blades, M.J., Kyriacou, C.P., Hastings, M.H., and Wilcockson, D.C. (2015). Metabolic molecular markers of the tidal clock in the marine crustacean *Eurydice pulchra*. Curr. Biol. *25*, R326–R327. https://doi.org/10.1016/j.cub.2015.02.052.
15. Hunt, B.J., Mallon, E.B., and Rosato, E. (2019). In silico Identification of a Molecular Circadian System With Novel Features in the Crustacean Model Organism *Parhyale hawaiensis*. Front. Physiol. *10*, 1325. https://doi.org/10.3389/fphys.2019.01325.
16. Zhang, L., Green, E.W., Webster, S.G., Hastings, M.H., Wilcockson, D.C., and Kyriacou, C.P. (2023). Correction: The circadian clock gene bmal1 is necessary for co-ordinated circatidal rhythms in the marine isopod *Eurydice pulchra* (Leach). PLoS Genet. *19*, e1011047. https://doi.org/10.1371/journal.pgen.1011047.
17. Kenning, M., and Harzsch, S. (2013). Brain anatomy of the marine isopod *Saduria entomon* Linnaeus, 1758 (Valvifera, Isopoda) with special emphasis on the olfactory pathway. Front. Neuroanat. *7*, 32. https://doi.org/10.3389/fnana.2013.00032.
18. Wittfoth, C., Harzsch, S., Wolff, C., and Sombke, A. (2019). The "amphi"-brains of amphipods: new insights from the neuroanatomy of *Parhyale hawaiensis* (Dana, 1853). Front. Zool. *16*, 30. https://doi.org/10.1186/s12983-019-0330-0.
19. Ramos, A.P., Gustafsson, O., Labert, N., Salecker, I., Nilsson, D.E., and Averof, M. (2019). Analysis of the genetically tractable crustacean *Parhyale hawaiensis* reveals the organisation of a sensory system for low-resolution vision. BMC Biol. *17*, 67. https://doi.org/10.1186/s12915-019-0676-y.
20. Hughes, M.E., Hogenesch, J.B., and Kornacker, K. (2010). JTK_CYCLE: An Efficient Nonparametric Algorithm for Detecting Rhythmic Components in Genome-Scale Data Sets. J. Biol. Rhythms *25*, 372–380. https://doi.org/10.1177/0748730410379711.
21. Thaben, P.F., and Westermark, P.O. (2014). Detecting Rhythms in Time Series with RAIN. J. Biol. Rhythms *29*, 391–400. https://doi.org/10.1177/0748730414553029.
22. Zheng, Y., Pan, L., Wang, F., Yan, J., Wang, T., Xia, Y., Yao, L., Deng, K., Zheng, Y., Xia, X., et al. (2023). Neural function of Bmal1: an overview. Cell Biosci. *13*, 1. https://doi.org/10.1186/s13578-022-00947-8.
23. Smyllie, N.J., Koch, A.A., and Hastings, M.H. (2025). Quantitative measures of clock protein dynamics in the SCN extends the circadian timekeeping model. EMBO J. https://doi.org/10.1038/s44318-025-00426-z.
24. Palmer, J.D. (1997). Dueling hypotheses: circatidal versus circalunidian battle basics–second engagement. Chronobiol. Int. *14*, 431–433. https://doi.org/10.3109/07420529709001463.
25. Takekata, H., Numata, H., and Shiga, S. (2014). The circatidal rhythm persists without the optic lobe in the mangrove cricket *Apteronemobius asahinai*. J. Biol. Rhythms *29*, 28–37. https://doi.org/10.1177/0748730413516309.
26. Takekata, H., Numata, H., Shiga, S., and Goto, S.G. (2014). Silencing the circadian clock gene Clock using RNAi reveals dissociation of the circatidal clock from the circadian clock in the mangrove cricket. J. Insect Physiol. *68*, 16–22. https://doi.org/10.1016/j.jinsphys.2014.06.012.
27. Tran, D., Perrigault, M., Ciret, P., and Payton, L. (2020). Bivalve mollusc circadian clock genes can run at tidal frequency. Proc. Biol. Sci. *287*, 20192440. https://doi.org/10.1098/rspb.2019.2440.
28. Schneider, C.A., Rasband, W.S., and Eliceiri, K.W. (2012). NIH Image to ImageJ: 25 years of image analysis. Nat. Methods *9*, 671–675. https://doi.org/10.1038/nmeth.2089.
29. Kikinis, R., Hata, N., Morrison, P.R., Cselik, Z., Black, P.M., and Jolesz, F. A. (2014). Intraoperative Imaging and Image-guided Therapy, F.A. Jolesz, ed. (Springer), pp. 567–574.
30. RStudio Team (2020). A language and environment for statistical computing (Vienna, Austria: R Foundation for Statistical Computing). https://www.R_project.org/.
31. Geissmann, Q., Garcia Rodriguez, L., Beckwith, E.J., and Gilestro, G.F. (2019). Rethomics: An R framework to analyse high-throughput behavioural data. PLoS One *14*, e0209331. https://doi.org/10.1371/journal.pone.0209331.
32. Pachitariu, M., and Stringer, C. (2022). Cellpose 2.0: how to train your own model. Nat. Methods *19*, 1634–1641. https://doi.org/10.1038/s41592-022-01663-4.
33. Bahry, E., Breimann, L., Zouinkhi, M., Epstein, L., Kolyvanov, K., Mamrak, N., King, B., Long, X., Harrington, K.I.S., Lionnet, T., et al. (2022). RS-FISH: precise, interactive, fast, and scalable FISH spot detection. Nat. Methods *19*, 1563–1567. https://doi.org/10.1038/s41592-022-01669-y.

34. Levine, J.D., Funes, P., Dowse, H.B., and Hall, J.C. (2002). Signal analysis of behavioral and molecular cycles. BMC Neurosci. *3*, 1. https://doi.org/10.1186/1471-2202-3-1.

35. Ruf, T. (1999). The Lomb-Scargle Periodogram in Biological Rhythm Research: Analysis of Incomplete and Unequally Spaced Time-Series. Biol. Rhythm Res. *30*, 178–201. https://doi.org/10.1076/brhm.30.2.178.1422.

36. Saver, M.A., Wilkens, J.L., and Syed, N.I. (1999). In Situ and In Vitro Identification and Characterization of Cardiac Ganglion Neurons in the Crab, *Carcinus maenas*. J. Neurophysiol. *81*, 2964–2976. https://doi.org/10.1152/jn.1999.81.6.2964.

37. Ostrovsky, A., Cachero, S., and Jefferis, G. (2013). Clonal analysis of olfaction in Drosophila: immunochemistry and imaging of fly brains. Cold Spring Harb. Protoc. *2013*, 342–346. https://doi.org/10.1101/pdb.prot071720.

38. Meissner, G.W., Grimm, J.B., Johnston, R.M., Sutcliffe, B., Ng, J., Jefferis, G.S.X.E., Cachero, S., Lavis, L.D., and Malkesman, O. (2018). DPX Mounting of Drosophila CNS. https://doi.org/10.17504/protocols.io.qj2duqe.

39. Bruce, H.S., Jerz, G., Kelly, S.R., McCarthy, J., Pomerantz, A., Senevirathne, G., Sherrard, A., Sun, D.A., Wolff, C., and Patel, N.H. (2021). Hybridization Chain Reaction (HCR) In Situ Protocol V.1. https://doi.org/10.17504/protocols.io.bunznvf6.

40. Costa, M., Manton, J.D., Ostrovsky, A.D., Prohaska, S., and Jefferis, G.S.X.E. (2016). NBLAST: Rapid, Sensitive Comparison of Neuronal Structure and Construction of Neuron Family Databases. Neuron *91*, 293–311. https://doi.org/10.1016/j.neuron.2016.06.012.

41. Bates, A.S., Manton, J.D., Jagannathan, S.R., Costa, M., Schlegel, P., Rohlfing, T., and Jefferis, G.S. (2020). The natverse, a versatile toolbox for combining and analysing neuroanatomical data. eLife *9*, e53350. https://doi.org/10.7554/eLife.53350.

42. Jefferis, G.S.X.E., Potter, C.J., Chan, A.M., Marin, E.C., Rohlfing, T., Maurer, C.R., Jr., and Luo, L. (2007). Comprehensive Maps of Drosophila Higher Olfactory Centers: Spatially Segregated Fruit and Pheromone Representation. Cell *128*, 1187–1203. https://doi.org/10.1016/j.cell.2007.01.040.

43. Rohlfing, T., and Maurer, C.R., Jr. (2003). Nonrigid image registration in shared-memory multiprocessor environments with application to brains, breasts, and bees. IEEE Trans. Inf. Technol. Biomed. *7*, 16–25. https://doi.org/10.1109/titb.2003.808506.

44. Choi, H.M.T., Schwarzkopf, M., Fornace, M.E., Acharya, A., Artavanis, G., Stegmaier, J., Cunha, A., and Pierce, N.A. (2018). Third-generation *in situ* hybridization chain reaction: multiplexed, quantitative, sensitive, versatile, robust. Development *145*, dev165753. https://doi.org/10.1242/dev.165753.

45. Wang, S. (2019). Single Molecule RNA FISH (smFISH) in Whole-Mount Mouse Embryonic Organs. Curr. Protoc. Cell Biol. *83*, e79. https://doi.org/10.1002/cpcb.79.

46. Stringer, C., Wang, T., Michaelos, M., and Pachitariu, M. (2021). Cellpose: a generalist algorithm for cellular segmentation. Nat. Methods *18*, 100–106. https://doi.org/10.1038/s41592-020-01018-x.

47. Sofroniew, N., Lambert, T., Bokota, G., Nunez-Iglesias, J., Sobolewski, P., Sweet, A., Gaifas, L., Evans, K., Burt, A., Doncila Pop, D., et al. (2024). napari: a multi-dimensional image viewer for Python (v0.5.0) (Zenodo).

STAR★METHODS

KEY RESOURCES TABLE

REAGENT or RESOURCE	SOURCE	IDENTIFIER
Antibodies		
3C11 (anti SYNORF1)	Developmental Studies Hybridoma Bank (DSHB)	RRID: AB_528479
anti-mouse Alexa 488	ThermoFisher	Cat#A-21141
F(ab')2-goat anti-mouse IgG-Alexa Fluor 488	ThermoFisher	Cat#A-11017
Alexa Fluor Plus 555	ThermoFisher	Cat#A48287
Fab goat anti-mouse IgG-Alexa Fluor 488	Jackson ImmunoResearch	Cat#115-547-003
Chemicals, peptides, and recombinant proteins		
Sea salt	Reef Salt, Tropical Marine Centre	N/A
RapiClear 1.49	SunJin Lab Optical Clearing Innovation	Cat#RC149001
DPX	Sigma Aldrich	Cat#06522
Prolong Glass Antifade Mountant	ThermoFisher	Cat#P36980
RNase inhibitor	New England BioLabs	Cat#M0314L
RNaseOUT™ ribonuclease inhibitor	Invitrogen	Cat#10777019
RNAscope® Multiplex Fluorescent Reagent Kit v2 Assay	ACD Biotechne	Cat#323100
Vectashield® Antifade mounting fluid	Vector Laboratories, 2B Scientific	Cat#H-1000-10ML
HCR™ Probe Hybridization Buffer	Molecular Instruments Inc.	LOT: BPH03725
HCR™ Probe Wash Buffer	Molecular Instruments Inc.	LOT: BPW03026
HCR™ Amplification Buffer	Molecular Instruments Inc.	LOT: BAM03526
Leica Paraplast Plus	Leica Biosystems	39602004
Experimental models: Organisms/strains		
Eurydice pulchra, wild caught		N/A
Parhyale hawaiensis, Chicago-F strain	Aziz Aboobaker	N/A
Oligonucleotides		
EpBmal1 HCR™ probe (v3.0)	Molecular Instruments, Inc., this paper	LOT: PRP939
EpBmal1 additional HCR™ probe (v3.0)	Molecular Instruments, Inc.	LOT: RTA402
EpClk 1-9 HCR™ probe (v3.0)	Molecular Instruments, Inc., this paper	LOT: RTA400
EpClk 1-9 additional HCR™ probe (v3.0)	Molecular Instruments, Inc., this paper	LOT: RTE285
Epcry2 HCR™ probe (v3.0)	Molecular Instruments, Inc., this paper	LOT: RTA399
Epcry2 HCR™ probe (v3.0)	Molecular Instruments, Inc., this paper	LOT: RTO453
Epper HCR™ probe (v3.0)	Molecular Instruments, Inc., this paper	LOT: RTO454
Eptim HCR™ probe (v3.0)	Molecular Instruments, Inc., this paper	LOT: PRP937
PhBmal1 HCR™ probe (v3.0)	Molecular Instruments, Inc., this paper	LOT: RTC080
Phper HCR™ probe (v3.0)	Molecular Instruments, Inc., this paper	LOT: RTK407; LOT: RTC082
PhClk-fragment 1 HCR™ probe (v3.0)	Molecular Instruments, Inc., this paper	LOT: RTG497
PhClk-fragment 2 HCR™ probe (v3.0)	Molecular Instruments, Inc., this paper	LOT: RTG498
Phcry2 HCR™ probe (v3.0)	Molecular Instruments, Inc., this paper	LOT: RTC083
deGFP HCR™ probe (v3.0)	Molecular Instruments, Inc.	LOT: RTA210
deGFP HCR™ probe (v3.0)	Molecular Instruments, Inc.	LOT: PRQ740
Epper-C4 RNAScope™ probe	ACD Biotechne, this paper	Cat#578211-C4
EpClk5-C1 RNAScope™ probe	ACD Biotechne, this paper	Cat#578151-C1
Epcry2-C1 RNAScope™ probe	ACD Biotechne, this paper	Cat#1039411-C1
Eptim-C3 RNAScope™ probe	ACD Biotechne, this paper	Cat#1099401-C3

(*Continued on next page*)

Continued

REAGENT or RESOURCE	SOURCE	IDENTIFIER
EpBmal1-C2 RNAScope™ probe	ACD Biotechne, this paper	Cat#578191-C2
-ve control RNAScope™ probe	ACD Biotechne	N/A
**Software and algorithms**		
FIJI ImageJ	Schneider et al., (2012) Nat Methods[28]	https://imagej.net/software/fiji/
Computational Morphometry Toolkit (CMTK)		https://www.nitrc.org/projects/cmtk
MakeAverageBrain		https://github.com/jefferislab/MakeAverageBrain
3DSlicer	Kikinis et al. (2014)[29]	https://www.slicer.org/
DAMSystem3 software	TriKinetics Inc.	N/A
R Studio	RStudio Team (2020)[30]	http://www.rstudio.com/
Rethomics	Geissmann et al.[28]	https://rethomics.github.io/
Cellpose 2.0	Pachitariu and Stringer[32]	https://www.cellpose.org/
napari	napari contributors	https://napari.org/
Fusion software	Andor, Oxford Instruments	N/A
RS-FISH ImageJ plugin	Bahry et al.[33]	https://github.com/PreibischLab/RS-FISH
Graphpad Prism		N/A
**Other**		
DAM10, LAM10, LAM25 activity monitors	Trikinetics Inc.	N/A
double-sided adhesive tape	Radio Spares	Cat#770-3422

EXPERIMENTAL MODEL AND STUDY PARTICIPANT DETAILS

Eurydice pulchra collection

Adult males and females were collected using a hand-towed net from Llanddona beach, Anglesey, UK, during spring high tides from May to October.[12]

Parhyale hawaiensis husbandry

Male and female Chicago F strain *P. hawaiensis* were maintained in 6.5 L polypropylene containers (h: 125 mm, w: 260 mm, d: 260 mm) containing 30–35 psu ASW and a layer of crushed coral substrate (grain size 2–20 mm). Animals were reared at 25°C under 12h light:dark (LD) cycles. The containers were covered with clear Perspex lids to prevent evaporation and aerated using aquarium air pumps and air stones. Animals were fed carrots, goldfish flakes (Tetra GmbH, Germany), algae wafers (Kyorin Food Ind. Ltd., Japan), freeze-dried brine shrimps, and/or freeze-dried Tubifex (Interpret, UK) once weekly. Full water changes were performed fortnightly.

METHOD DETAILS

Eurydice pulchra behavior analysis

Swimming behaviour of adult females and males was recorded in DAM10 and LAM10 activity monitors (Trikinetics Inc., USA) under DD at 16°C with animals housed individually in 3 ml (Ø: 11 mm) plastic vials containing 33 psu artificial seawater (ASW, Reef Salt, Tropical Marine Centre, Germany) and ~5 mm depth of fine sand. Infrared beam interruptions were recorded using the DAMSystem3 software (TriKinetics Inc., USA) in one-minute bins. The first peak in tidal swimming activity was omitted from analysis and behavioural rhythmicity was assessed over the subsequent six tidal cycles using chi-squared periodogram analysis in Rethomics R package.[31]

Parhyale hawaiensis behavior analysis

For locomotor activity monitoring, adult female and male animals were placed individually into 23 mL polystyrene vials (h: 75 mm, Ø: 23.5 mm) containing a layer (~2 mm) of crushed coral substrate (grain size 2–5 mm) and approximately 6 cm of 30 psu ASW at 25°C. Tubes were loosely covered with parafilm and loaded into two-tier activity monitors modified from the LAM25 Locomotor Activity Monitors (TriKinetics Inc., USA) configured to record swimming and/or roaming activity. Infrared beam interruptions were recorded using the DAMSystem3 software (TriKinetics Inc., USA).

Beam interruptions were collected into 30-minute bins and normalised within each animal by dividing the count in each bin by the average count across all bins for that animal. Autocorrelation periodogram was used to determine whether animals were rhythmic,

with a significance threshold of α = 0.05.[34] The period and the power of rhythmicity were determined using Lomb-Scargle periodogram analysis[35] of binned and normalised data at the significance level α =0.05. For circatidal experiments, the first subjective high tide after loading was omitted from analysis and behavioural rhythmicity was assessed over the subsequent six tidal cycles. For circadian entrainment experiments, animals loaded into the monitor were subjected to at least four 12:12 LD (ZT0/lights-on at 0600 local time) cycles before being allowed to free run in DD. For tidal entrainment, animals were kept under LD as above but mechanically agitated on an orbital shaker set at 50–60 rpm for 2 hours at 12.4-hour intervals. For behavioural monitoring, animals were taken from entrainment regimes during the agitation phase and placed in the activity monitor as described above to free-run without agitation in DD.

Brain collection and processing

Animals were anaesthetised in ice-cold seawater, and their brains dissected using sharpened watch-maker's forceps in ice-chilled physiological saline[36] (*E. pulchra*) or 0.22 μm filter-sterilised ASW (30 -35 psu, FASW; *P. hawaiensis*). Tissues were immediately fixed in 4% paraformaldehyde (made in PBS for *E. pulchra* and FASW for *P. hawaiensis*) for 1 hour at room temperature, dehydrated serially in 33%, 66%, 100% methanol in PBST (PBS supplemented with 0.1% Tween20), and stored in 100% methanol at -20°C for at least 16 h before hybridisation or immunohistochemistry. Immediately prior to use, brains were rehydrated in 66/33% methanol and washed/equilibrated in PBST (3x10 min).

Immunohistochemistry

Neuropils were immunolabelled using anti-SYNORF1 antibody (3C11, Developmental Studies Hybridoma Bank (DSHB)) following.[37,38] 3c11 (anti SYNORF1) is a mouse monoclonal anti-body against *Drosophila* synapsin and with confirmed reactivity against crustaceans (as well as insects, cephalopods, leech and planaria). For *E. pulchra*, after rehydration to PBST, brains were washed in PTx (PBS supplemented with 0.1% Triton X-100), permeabilised in detergent solution (50 mM Tris-HCl pH 7.5, 150 mM NaCl, 1 mM EDTA pH 8, 1% SDS, 0.5% Tween-20) for 30 min at room temperature[39] and then incubated with 1:50 anti-SYNORF1 in PTx containing 5% normal goat serum (NGS) overnight at room temperature, followed by 5 days at 4°C, with rotation. After extensive washing in PTx, brains were incubated with anti-mouse Alexa 488 (ThermoFisher #A-21141) at 1:1000 in PTx containing 5% NGS for 3 days at 4°C, with rotation. After extensive washing with PTx, nuclei were stained with 0.1 μg/mL DAPI int PTx for 1h. Brains were then bridge-mounted in RapiClear 1.49 (SunJin Lab Optical Clearing Innovation, #RC149001) using No. 2 thickness coverslips to maintain 3D morphology. For *P. hawaiensis*, brains were incubated in blocking buffer (1x PBS, 0.5% Tween-20, 5% NGS, 1% bovine serum albumin) containing 1:50 anti-SYNORF1 for 3 days at 4°C with gentle shaking, followed by extensive washing in PTw (PBS supplemented with 0.5% Tween-20) and incubation in secondary antiserum F(ab′)2-goat anti-mouse IgG-Alexa Fluor 488 (ThermoFisher #A-11017) or Alexa Fluor Plus 555 (ThermoFisher #A48287) at 1:250 made in blocking buffer for 2 days at 4°C with gentle agitation. Nuclei were stained with 0.1 μg/mL DAPI for 30 min prior to final washing in PTw and preparations mounted in DPX (Sigma Aldrich #06522) according to.[38]

Reference brains

Whole-mount, immunolabelled (see above) brain samples used for generating reference brains and describing the neuroanatomy of putative clock cells were imaged on a Nikon CSU-W1 spinning disk confocal microscope equipped with a 25x/1.05NA silicone oil CFI Plan Apochromat Lambda S objective and a Prime 95B 22mm sCMOS camera. To make an *E. pulchra* reference brain, two sex-specific references were made by registering and then averaging 7 male brains to one male "seed" brain and 6 female brains to a female "seed" brain. These sex-specific references were then averaged to generate a final intersex reference brain (an average of 15 brains) used for all subsequent analyses. For *P. hawaiensis*, 10 male brains were registered and then averaged to a seed brain to generate a reference brain. Registration and averaging were done using: https://github.com/jefferislab/MakeAverageBrain.[40,41] Briefly, Computational Morphometry Toolkit (CMTK) was used to register image stacks against a seed image stack; first with a rigid and then a non-rigid registration.[42,43] Image stacks of brains with co-immuno-RNA-FISH labelling of neuropils and circadian clock genes (*per/ cry2/ tim*) were registered to reference brains based on the anti-SYNORF1 signal. Three-dimensional surface models of neuropils were based on reference brains and generated using the Segmentation Editor in 3DSlicer software (www.slicer.org). 3D surface models of putative clock cells were based on circadian clock gene HCR-FISH labelling (*Epcry2* or *Phcry2*) in an image stack registered to the reference brain and using the Segmentation Editor in 3DSlicer.

HCR™ RNA-FISH

HCR™ probes (v3.0) were designed and synthesised by Molecular Instruments Inc, USA against user-supplied sequences (Table S8). HCR-FISH followed the manufacturer's protocol[44] with some minor alterations following.[39] For HCR-FISH, rehydrated brains were permeabilised in detergent solution for 30 min at room temperature, and equilibrated in pre-warmed probe hybridisation buffer for 30 min at 37°C. Brains were then incubated in probe hybridisation solution containing 10 nM of each primary probe set for 2 days (*E. pulchra*) or 3 days (*P. hawaiensis*) at 37°C. For *P. hawaiensis*, 1:1000 murine RNase inhibitor (NEB #M0314L) was added to the probe hybridisation buffer. Signal amplification with 60 nM fluorescently tagged hairpins was allowed to proceed for 2 days, followed by staining with 0.1 μg/mL DAPI in 5x SSC supplemented with 0.5% Tween-20 (30 min) during one of the final washing steps. *E. pulchra* brains were bridge-mounted using No. 1.5 thickness coverslips in RapiClear 1.49 whilst *P. hawaiensis* brains were dehydrated and mounted in DPX according to.[38] The target genes used for probe design, their accession numbers, and the Molecular

CellPress
OPEN ACCESS

Instruments probe lot numbers and concentrations used are listed in Table S8. For experiments investigating co-expression of clock genes, a 40x/1.3NA Plan Fluorite oil immersion objective was used to image with an optical section interval of 1 μm.

Co-immuno-RNA-FISH

To co-localise putative clock cells relative to major neuropils, immunostaining with anti-SYNORF1 antibody was followed by HCR-FISH. Reference brains were generated from confocal image stacks of brains in which neuropils were labelled with anti-SYNORF1 (see above). For co-immuno-RNA-FISH staining, following rehydration and permeabilization in detergent solution, *E. pulchra*, brains were incubated with 1:50 anti-SYNORF1 in PTx containing 1.6 U/μL RNaseOUT™ ribonuclease inhibitor (Invitrogen #10777019) overnight at room temperature, followed by 3 days at 4°C, with rotation. After extensive washing in PTx, brains were incubated with anti-mouse Alexa 488 (ThermoFisher #A-21141) at 1:1000 in PTx containing RNase inhibitor for 2 days at 4°C, with rotation. After washing in PTx/w, brains were fixed in 4% PFA (in PBS) for 1 hour at room temperature. After washing in PTx/w, the neuropil-stained brains were processed for HCR-FISH as above and mounted as described above. *P. hawaiensis* brains were washed in PTw and subjected to one-step immunostaining[45] by incubating in PTw containing pre-mixed 1:250 murine RNase inhibitor, 1:50 anti-SYNORF1, 3.33 ng/μL Fab goat anti-mouse IgG-Alexa Fluor 488 (Jackson ImmunoResearch #115-547-003) for 2 days at room temperature.

RNAscope methods for formalin-fixed, paraffin-wax embedded *E.pulchra* brains

E. pulchra brains were dissected in ice-chilled crustacean saline[36] and fixed immediately in 10% normal buffered formalin (10%) overnight (16h) at RT. After fixing, brains were washed extensively in PBS, dehydrated through an ethanol series (50%, 70%, 80%, 90%, 95%, 100%), cleared in xylene (1h at RT) and infiltrated in molten wax (Leica Paraplast Plus®, Leica Biosystems, Sheffield UK) 3x, each for 1h, before embedding in wax such that the brains could be mounted for frontal sections to be cut. Serial sections (6μm) were cut on a standard wax microtome and mounted on Superfrost™ Plus (Thermo Fisher, UK). After drying, sections were baked at 60°C for 2h to adhere fully to the slides. Slides were de-waxed in clean xylene and taken to water through an ethanol series before drying at 60C for 1h. Following preparation, manufacturer's instructions were followed exactly, except that target retrieval was done in 700 mL Target Retrieval buffer in a 1L beaker, for 15 minutes at 98-104°C, over a hot-plate. Protease Plus was applied for 30 minutes at 40°C. Signal amplification was done using Opal™ fluors (Opal 520 and Opal 570). All reagents and kit components were as recommended by ACD Biotechne (Abingdon, Oxford, UK. RNAscope® Multiplex Fluorescent Reagent Kit v2 Assay). Target probes were designed by ACD Biotechne and probe details provided in Table S9. Negative control probes were supplied by ACD Biotechne and are designed against the *DapB2* gene of *Bacillus subtilis*. Following hybridisation and amplification, sections were counterstained with DAPI provided with the kit and mounted under Vectashield® Antifade mounting fluid (Vector Laboratories, supplied by 2B Scientific, Oxford, UK). Images were captured on a Leica SP8 confocal microscope at 1μm z-stack intervals and using Leica LAX proprietary software. Images were re-sized, cropped and adjusted for brightness and contrast using ImageJ software.

Time-course sampling design

To investigate clock gene expression pattern in the brain of *E. pulchra*, animals were collected from Llanddona beach, Anglesey, UK, during spring high tides in October 2024 and loaded into activity monitors. Prior to sampling, animals were maintained under 18 h: 6 h L:D cycle for 24 hours. Animals showing peaks of swimming activity during the two subjective high waters during this period were sampled every two hours, beginning at ZT7. To investigate daily clock gene expression pattern in the brain of *P. hawaiensis*, animals were loaded into activity monitors and subjected to 12 h: 12 h LD cycle for 6 days as described. Individuals showing significant 24-h rhythmicity in locomotor activity (as described) over this entrainment period up to the day before sampling were marked for sampling. On the day of sampling, marked animals were sampled every 2 hours, beginning at ZT1 and ending at ZT25, for a total of 13 timepoints.

To investigate circatidal clock gene expression pattern in the brain of tidally entrained *P. hawaiensis*, animals which have been subjected to tidal entrainment were loaded into activity monitors as described. Sampling occurred every 3 hours, beginning at CT0 on the day after monitor loading and ending at CT24, for a total of 9 timepoints. Individuals were only sampled if they showed peaks of locomotor activity during the subjective high tides preceding their sampling timepoint.

Dissected brains were processed for digital HCR-FISH as described. HCR-FISH probes used for time-course experiments are described in Table S10.

Whole brain digital HCR-FISH

For quantification of diffraction-limited spots,[44] signal amplification with 60 nM of each hairpin was performed for 1 h, followed by staining with 1 μg/mL DAPI (as above). Brains were mounted in Prolong Glass Antifade Mountant (ThermoFisher #P36980). *E. pulchra* brains were mounted with the posterior surface apposed to the coverslip whilst *P. hawaiensis* brains were mounted with the anterior surface apposed. To minimise signal loss with imaging depth, brains were compressed to within 40 μm by mounting the coverslips containing brains onto spacers made from double-sided adhesive tape (Radio Spares #770-3422). An Andor BC43 CF benchtop microscope equipped with a 60x/1.42NA Plan Apochromat oil immersion objective was used to image each brain with an optical section interval of 0.3 μm.

To prepare images for segmentation of cell groups enriched for expression of *per*, *cry2*, and/ or *Clk*/ *tim*, 2D maximum filters were applied to each HCR-FISH channel, followed by summation of the filtered images across channels and 2D gaussian filtering. The resulting images, together with the corresponding raw DAPI image as the auxiliary channel, were subjected to segmentation using

Cellpose[46] and a custom model (available at GitHub) trained via the human-in-the-loop functionality of the Cellpose 2.0 graphical user interface.[32] The resulting masks were manually curated as required in napari[47] and assigned to distinct cell groups. For spot detection, images were deconvolved using ClearView™ Deconvolution with 10 iterations after being acquired in the Fusion software. RNA foci were then detected in deconvolved images and assigned to cell groups using the RS-FISH ImageJ plugin.[33]

QUANTIFICATION AND STATISTICAL ANALYSIS

Statistical details of test performed are reported in figure legends and Tables S2–S7. Graphs present data as mean ±SEM (unless otherwise stated). Chi-squared (*E. pulchra*) and Lomb-Scargle (*P. hawaiensis*) periodogram analysis were used to determine power and period of locomotor activity. For quantification of transcript abundance by FISH spot counts, Shapiro-Wilk test was used to determine normal distribution of data and Levene's test was used to determine if the variances of all groups are equal. An overall time effect was tested by ANOVA for normally distributed data and by Kruskal-Wallis for non-normal datasets. Where this yielded a statistically significant effect, rhythmicity in the data was analysed further by JTK CYCLE[20] and RAIN[21] at 12-hour or 24-hour periods. JTK-cycle p-values are adjusted using Bonferroni's method, and RAIN p-values are adjusted using the adaptive Benjamini-Hochberg (default) method. Gene expression was considered rhythmic when ANOVA was significant, and both JTK CYCLE and RAIN were significant for the same period (12 hours or 24 hours).

Current Biology, Volume *35*

Supplemental Information

Expression of clock genes tracks daily and tidal time in brains of intertidal crustaceans *Eurydice pulchra* and *Parhyale hawaiensis*

Andrew Oliphant, Chee Y. Sia, Charalambos P. Kyriacou, David C. Wilcockson, and Michael H. Hastings

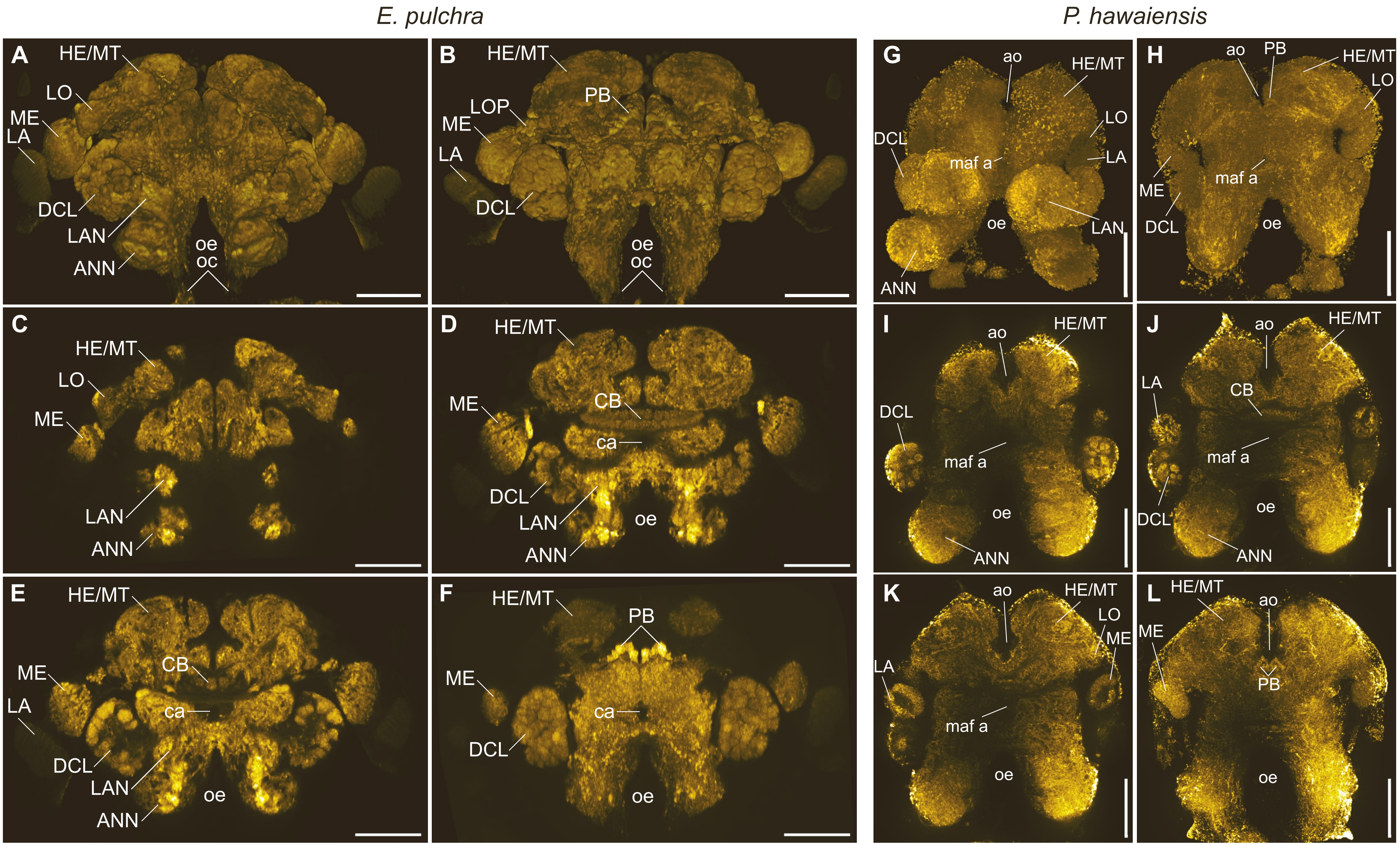
E. pulchra
P. hawaiensis
A
HE/MT
LO
ME
LA
DCL
LAN
ANN
oe
oc
B
PB
LOP
C
D
CB
ca
E
F
G
ao
maf a
H
I
J
K
L

Figure S1. Overview of representative *E. pulchra* and *P. hawaiensis* brains, immunostained with anti-SYNORF1 to highlight the neuropils. Related to Figures 1 and 2.

(A) Anterior view of a *E. pulchra* brain (3D volume projection), with optic lobes, deutocerebral chemosensory lobes, antenna neuropils and hemi-ellipsoid body/medulla terminalis complexes visible on the anterior surface of the brain.

(B) Posterior view of a *E. pulchra* brain (3D volume projection). The optic lobes, deutocerebral chemosensory lobes and hemi-ellipsoid body/medulla terminalis complexes are visible on the posterior surface. The protocerebral bridge appears as a distinct arch-like structure under the hemi-ellipsoid body/medulla terminalis complexes.

(C- E) Selected optical sections of a *E. pulchra* brain from anterior to posterior, showing locations of distinct neuropils throughout the depth of the brain. The optic lobes span the anterior-posterior axis, with the lobula constrained to the anterior aspect (C), and the medulla and lamina (D, E) occupying the medial and posterior aspects. The cigar-shaped central body is at its most expansive towards the middle of the brain.

(F) Maximum intensity Z-projection through the protocerebral bridge of a *E. pulchra* brain.

(G) Anterior view of a *P. hawaiensis* brain (3D volume projection), with parts of the optic lobes (lamina and lobula), deutocerebral chemosensory lobes, antenna neuropils and hemi-ellipsoid body/medulla terminalis complexes visible on the anterior surface of the brain.

(H) Posterior view of a *P. hawaiensis* brain (3D volume projection). Parts of the optic lobes (medulla and lobula), deutocerebral chemosensory lobes and hemi-ellipsoid body/medulla terminalis complexes are visible on the posterior surface. As in *E. pulchra,* the protocerebral bridge of *P. hawaiensis* is located posteriorly under the hemi-ellipsoid body/medulla terminalis complexes.

(I- K) Selected optical sections of a *P. hawaiensis* brain from anterior to posterior, showing locations of distinct neuropils throughout the depth of the brain. The hemi-ellipsoid body/medulla terminalis complexes and the foramina accommodating two of the brain arteries, the anterior aorta and myoarterial formation a, are visible throughout the thickness of the brain. The former also constitute the most dorsal neuropils of the brain. The deutocerebral chemosensory lobes are located anterior to the rest of the optic lobes (I, J). The lamina is the most anteriorly positioned optic neuropils (J), followed almost simultaneously by the medulla and lobula (K). The sausage-shaped central body is at its most expansive towards the middle of the brain (J).

(I) Maximum intensity Z-projection through the protocerebral bridge of a *P. hawaiensis* brain.

Abbreviations: ANN, antenna 2 neuropil; ao, anterior aorta; ca, cerebral artery; CB, central body; DCL, deutocerebral chemosensory lobe; HE/MT, hemi-ellipsoid body/medulla terminalis; LA, lamina; LAN, lateral antenna 1 neuropil; LO, lobula; LOP, lobula plate; maf a,

myoarterial formation a; ME, medulla; oc, oesophageal connective; oe, oesophageal foramen; PB, protocerebral bridge. Scale bars*:* 100 μm.

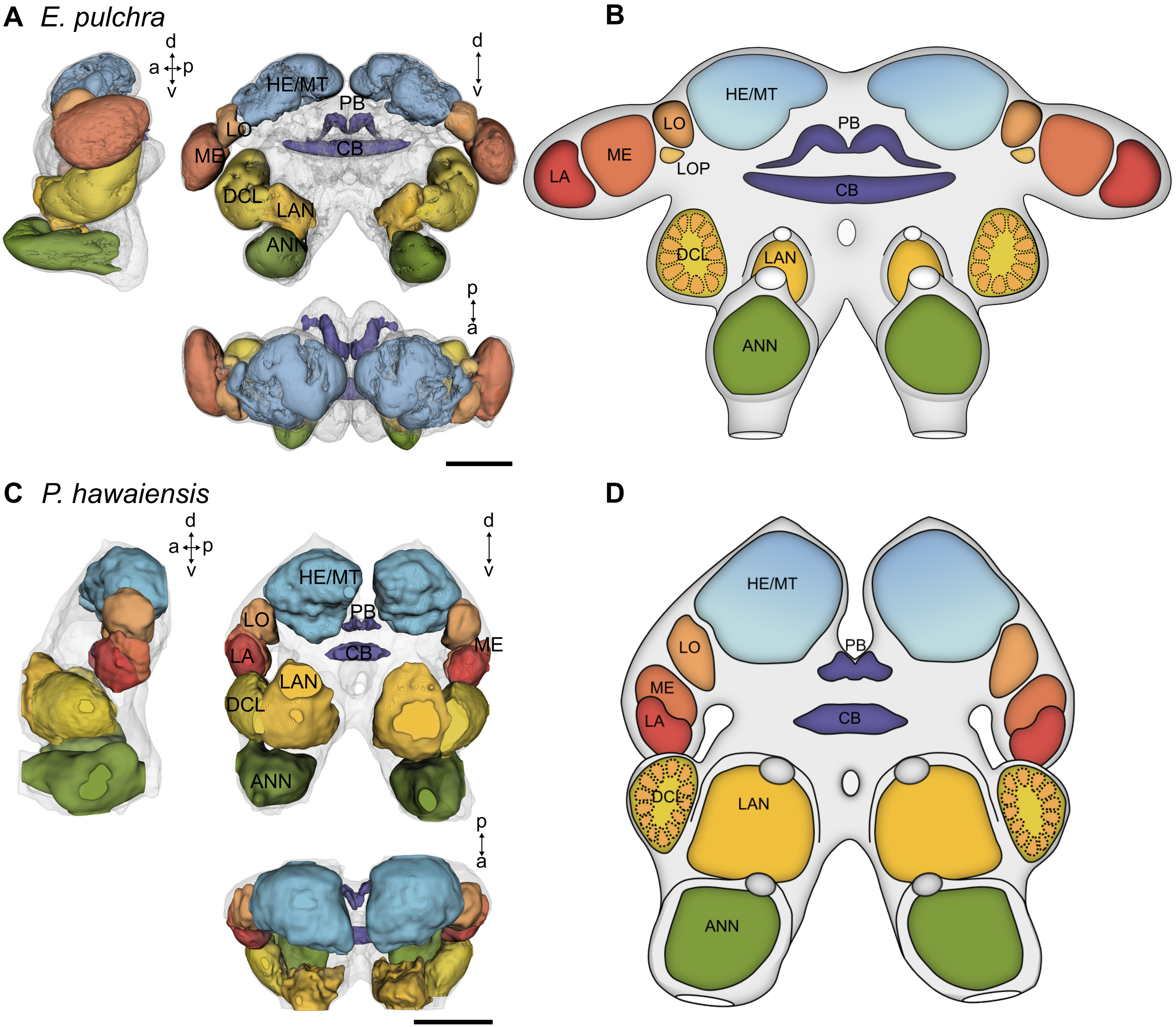

A E. pulchra
B
C P. hawaiensis
D
d
a
p
v
HE/MT
PB
LO
CB
ME
DCL
LAN
ANN
LA
LOP

Figure S2. Gross anatomy of *E. pulchra* and *P. hawaiensis* brains revealed by anti-SYNORF1 immunostaining, registered to reference brain. Related to Figures 1 and 2.

(A) Lateral, anterior and dorsal views of the *E. pulchra* surface-rendered 3D average reference brain, with major neuropils demarcated. See also Video S1.

(B) Diagrammatic representation of the of *E. pulchra* brain, based on A.

(C) Lateral, anterior and dorsal views of *P. hawaiensis* surface-rendered 3D average reference brain with major neuropils demarcated. See also Video S2.

(D) Diagrammatic representation of the *P. hawaiensis* brain, based on C.

Compass markers in A and C show anterior (a), posterior (p), dorsal (d) and ventral (v) directions.

Abbreviations: ANN antenna 2 neuropil, CB central body, DCL deutocerebral chemosensory lobe, HE/MT medulla terminalis/ hemi-ellipsoid body, LA lamina, LAL lateral accessory lobe, LAN lateral antenna 1 neuropil, LO lobula, LOP lobula plate, ME medulla, PB protocerebral bridge. Scale bars: 100 µm.

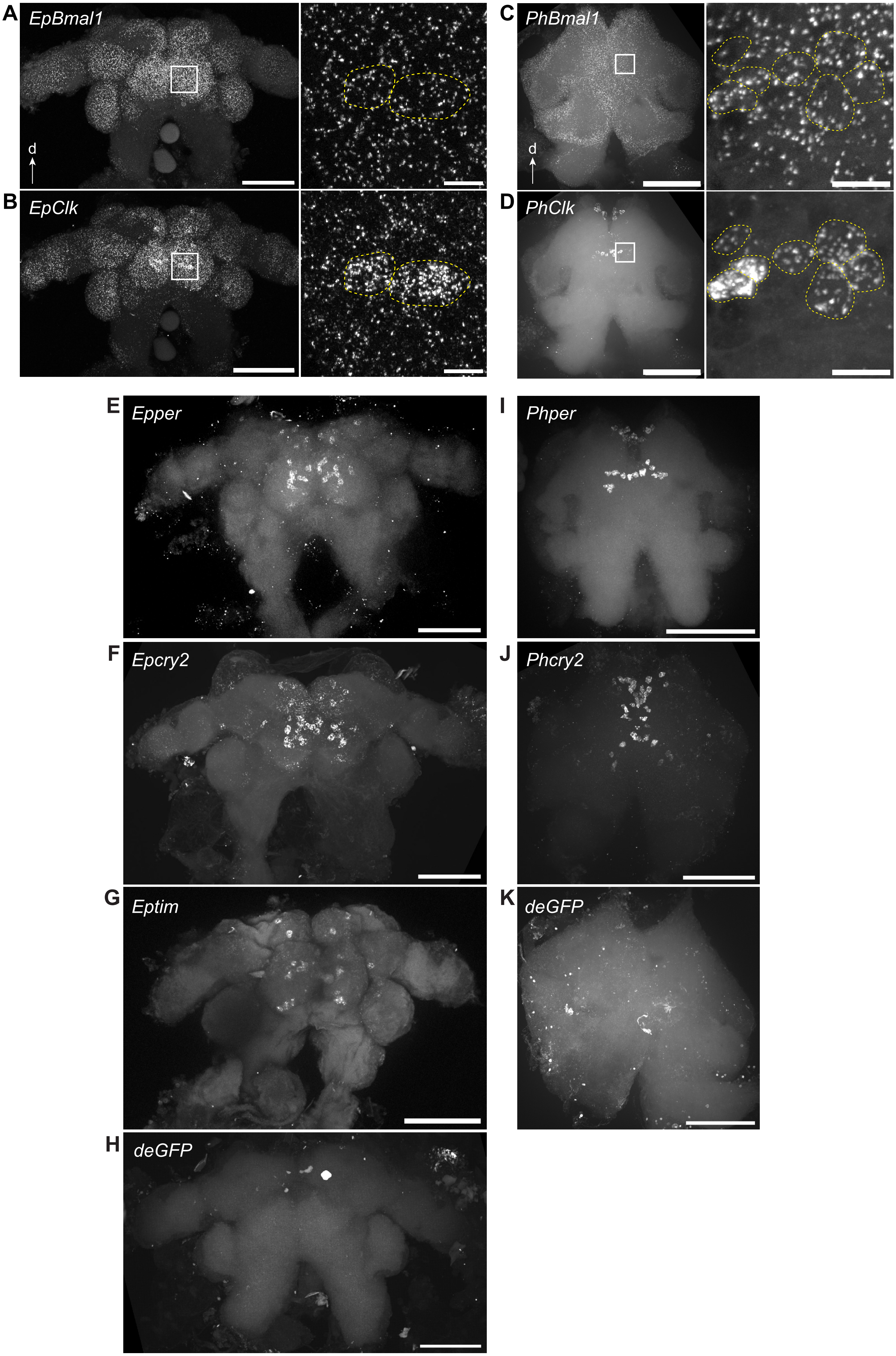
A
EpBmal1
d
B
EpClk
C
PhBmal1
d
D
PhClk
E
Epper
F
Epcry2
G
Eptim
H
deGFP
I
Phper
J
Phcry2
K
deGFP

Figure S3. Expression of circadian clock positive and negative regulators across the brains of *E. pulchra* and *P. hawaiensis*. Related to Figures 1 and 2.

(A) Left panel: maximum intensity Z-projection of image stack of an entire *E. pulchra* brain (posterior apposing coverslip) probed for circadian factor *EpBmal1* using HCR-FISH. Right panel: maximum intensity Z-projection of two-cell-thick image stack (16 μm) from the boxed region of the brain in the left panel.

(B) As in A, probed for circadian factor, *EpClk*.

(C) Left panel: maximum intensity Z-projection of image stacks of a *P. hawaiensis* brain (anterior apposing coverslip) probed for circadian factor *PhBmal1*. Right panel: maximum intensity Z-projection of two-cell-thick image stacks (24 μm) from the boxed region of the brain in the left panels.

(D) As in C, probed for circadian factor, *PhClk*.

(E, F, G, H) Maximum intensity Z-projections of image stacks of *E. pulchra* brains (posterior apposing coverslip) probed for circadian clock repressors, *Epcry2, Epper*, *Eptim* and the negative control probe *deGFP* HCR-FISH.

(I, J, K) Maximum intensity Z-projections of image stacks of *P. hawaiensis* brains (anterior apposing coverslip) probed for circadian clock repressors, *Phcry2*, *Phper* and the negative control probe *deGFP*. Scale bars: 100 μm.

Arrows indicate dorsal orientation. Yellow dotted outlines in A- D indicate cells in which *Clk* expression is enriched. Scale bars: 100 μm (A-D: left panels, E-K) and 10 μm (A-D: right panels).

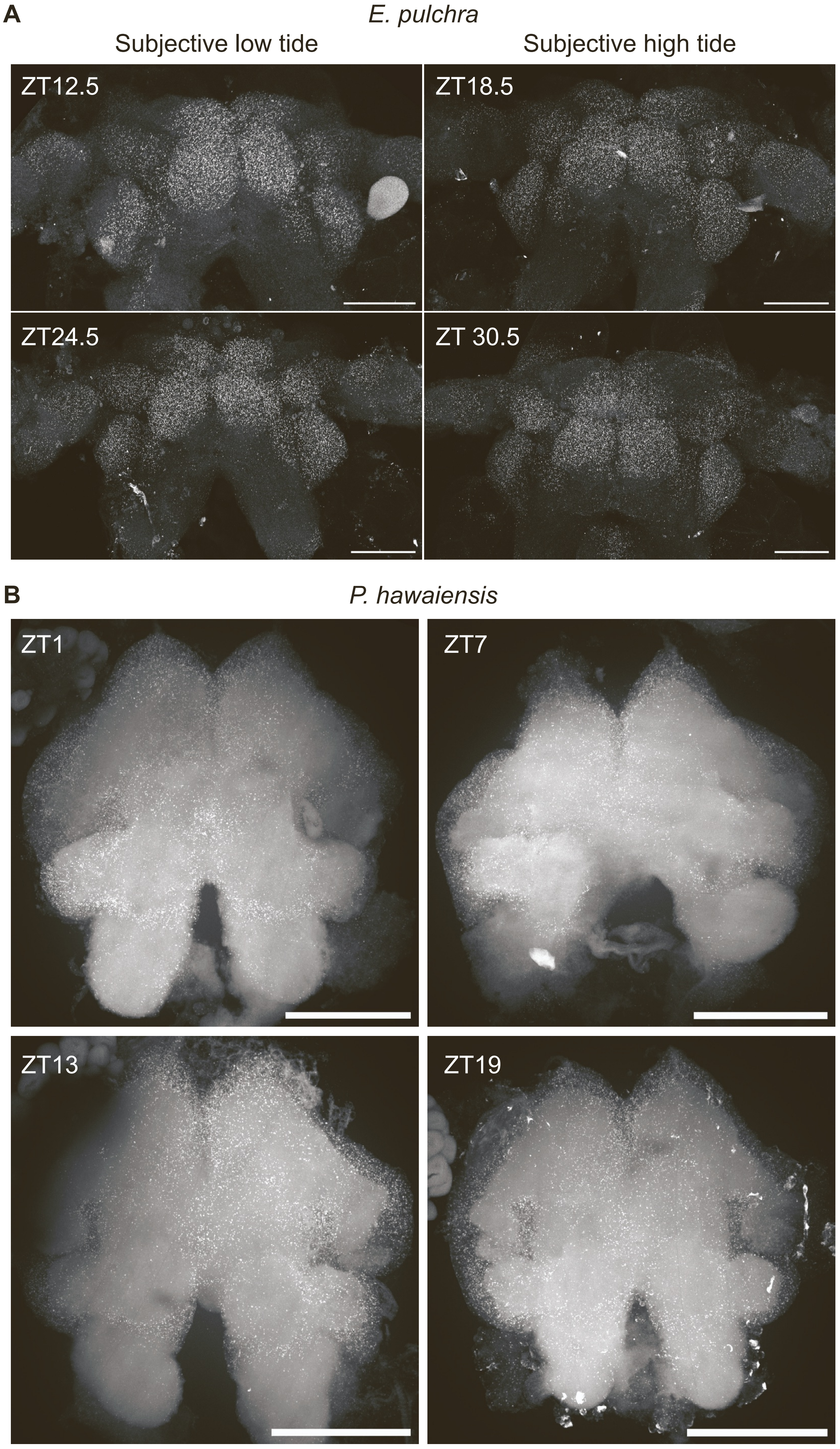
A
E. pulchra
Subjective low tide
Subjective high tide
ZT12.5
ZT18.5
ZT24.5
ZT 30.5
B
P. hawaiensis
ZT1
ZT7
ZT13
ZT19

Figure S4. Cellular expression of *Bmal1* in brains of *E. pulchra* and *P. hawaiensis* across time. Related to Figures 1 and 2.

(A) Maximum intensity Z-projections of representative brains (posterior apposing coverslip) from field-collected *E. pulchra* sampled under 14 h:10 h LD cycle and then probed for *EpBmal1* using HCR-FISH.

(B) Maximum intensity Z-projections of representative *P. hawaiensis* brains (anterior apposing coverslip) sampled under 12 h: 12 h LD cycle and then probed for *PhBmal1* using HCR-FISH. Scale bars: 100 μm.

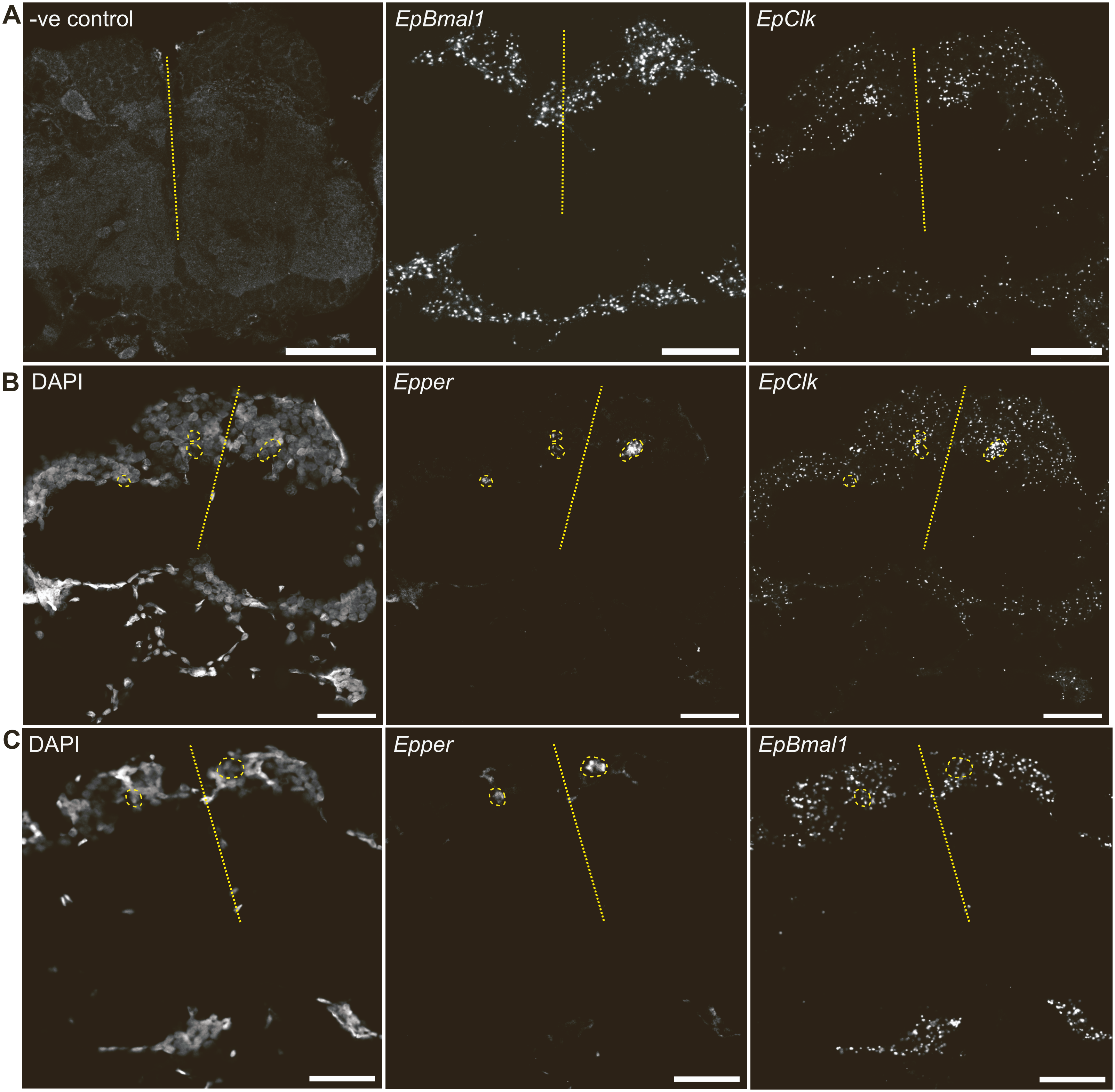
A
-ve control
EpBmal1
EpClk
B
DAPI
Epper
EpClk
C
DAPI
Epper
EpBmal1

Figure S5. Expression of circadian clock genes in *E. pulchra* brain sections detected using RNAScope™. Related to Figures 1 and 2.

(A) Representative image (single optical section) of *E. pulchra* brain sections probed for negative control (left), *EpBmal1* (middle) and *EpClk* (right).

(B) Representative image (single optical section) of a *E. pulchra* brain section stained with DAPI (left) and probed for *Epper* (middle) and *EpClk* (right).

(C) As in B for *Epper* and *EpBmal1*.

Yellow dashed outlines indicate cells enriched for *Epper* and *EpClk* expression. Yellow dotted lines indicate midlines. Scale bars: 10 μm.

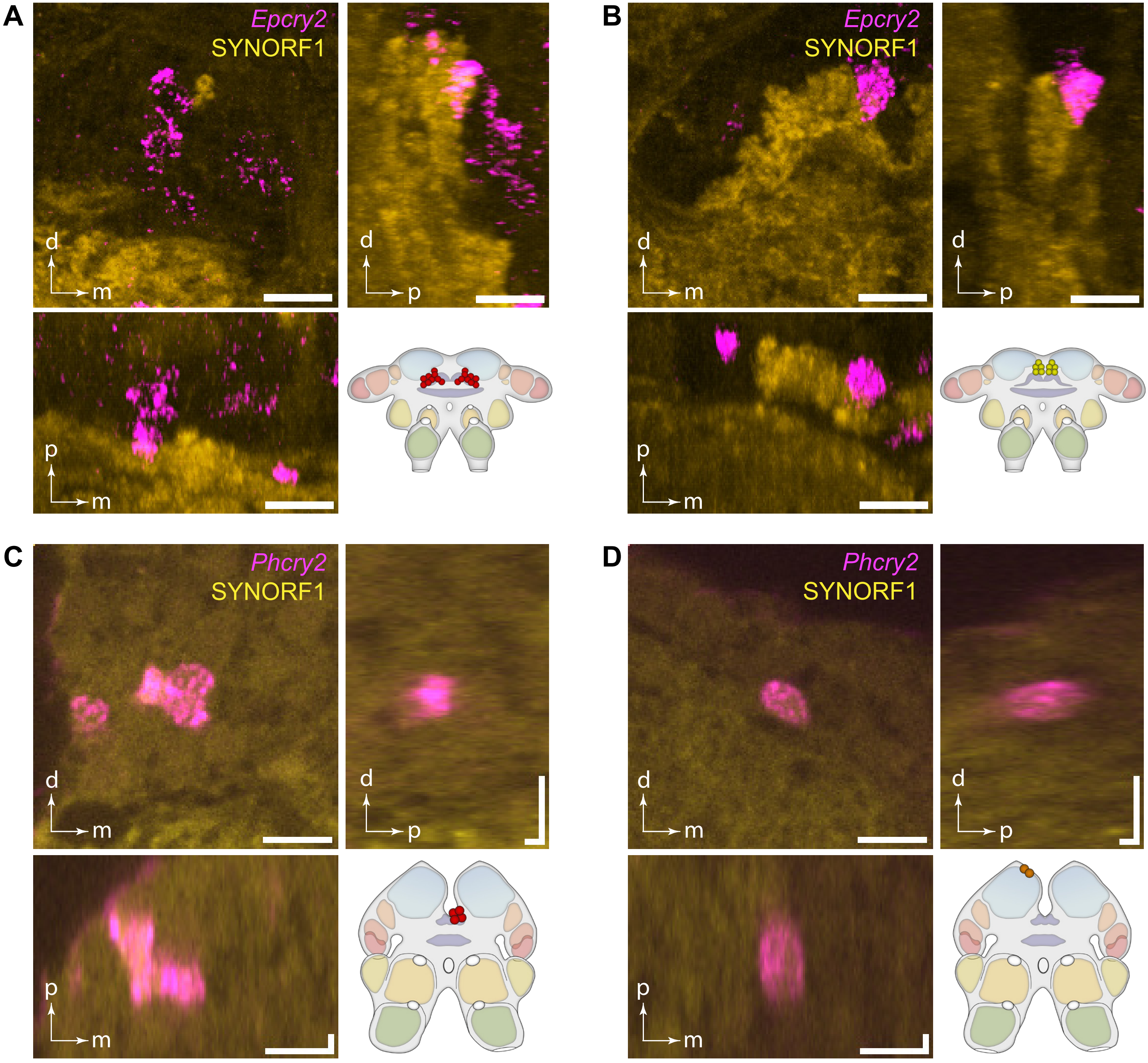
A
Epcry2
SYNORF1
d
m
d
p
p
m
B
Epcry2
SYNORF1
d
m
d
p
p
m
C
Phcry2
SYNORF1
d
m
d
p
p
m
D
Phcry2
SYNORF1
d
m
d
p
p
m

Figure S6. Representative orthogonal views of brains co-labelled with anti-SYNORF1 (IHC) and *cry2* (HCR-FISH). Related to Figures 1 and 2.

(A) Orthogonal views showing *Epcry2* enrichment in four medioposterior cells of a *E. pulchra* brain co-stained with anti-SYNORF1. Cartoon shows location of these cells.

(B) As in A but for the medial cells.

(C) Orthogonal views showing *Phcry2* enrichment in four medioposterior cells of a *P. hawaiensis* brain co-stained with anti-SYNORF1. Cartoon shows location of these cells.

(D) As in C but for the two dorsal-lateral cells.

Horizonal (and vertical) scale bars: 10 µm. Orientations: d: dorsal; p: posterior; m: medial.

In *E. pulchra, per-* and *cry2*-expressing cells were primarily found in the posterior cell body rind overlying the medial protocerebrum (MP), hemi-ellipsoid body and medulla terminalis (HE/MT, Figure 2A, B). There were nine somata in the rind posterior to the protocerebral bridge (rPBp) [S 1], which we termed the "medioposterior" cells. Dorsal and anterior to these cells was a cluster of 5 somata in the cell body rind dorsal to the PB (rPBd) that we assigned as "medial" cells. Lateral to these medial and medioposterior cells was a cell pair in the rind posterior of the medulla terminalis (rMTp) which we named "lateroposterior" cells. In the cell body rind posterior to the hemi-ellipsoid body/ medulla terminalis (rHEp/MTp) were 4 "dorsal" cells, one of which was larger than the others and we refer to as "large dorsal" cell. In the dorsal region of the cell body rind posterior of the MP (rMPp) ~4-6 "ventroposterior" cells. In the anterior aspect of the brain, we found fewer cells; in the cell body rind lateroanterior of the hemi-ellipsoid body/medulla terminalis (rHE/MTla) was a single pair of cells, the "dorsoanterior" cells. A single "lateroanterior" cell was in the rind ventral and anterior of the lobula (rLOva). Finally, a pair of cells on the midline (one per hemisphere), anterior of the medial protocerebrum were named "medioanterior" cells (rMPa). Cell groups and their given nomenclature are summarised in Table S1.

In *P. hawaiensis*, *per*- and *cry2*-enriched cells were found throughout the anterior and posterior aspects of cell body rind overlying MP and HE/MT. In the cell body rind posterior and lateral to the PB (rPBp) were ten *per*- and *cry2*-enriched somata that we assigned as "medioposterior" cells (Figure 2C, D). Dorsoanterior to these cells were three cells, two of which were closer to each other than the last one; these we termed "medial-2" and "medial-1", respectively, and collectively, the medial cells. In the cell body rind dorsal of the HE/MT (rHE/MTd), we identified two somata as the "dorsal-lateral" cells, and antero-medial to these cells were six cells that

we called the “dorsal” cells. On the anterior aspect of the brain, we identified one cell in the cell body rind anterior of the HE/MT (rHE/MTa) as “anterior-lateral” cells. Lastly, we found 8 – 9 cells on the anterior aspect of the medial protocerebrum (rPMa). We refer to these cells collectively as the “anterior-medial” cells and they can be divided into 4 groups. “Anterior-medial b” cells described two cells with distinctive location lateral to the myoarterial formation. Dorsal to the anterior-medial b cells were two cells, in which one was positioned medially to the other. These, we termed “anterior-medial a1” and “anterior-medial a2” cells, respectively. The “anterior-medial c” cells were a group of 4 – 5 cells located dorsal to the anterior-medial a1-2 cells.

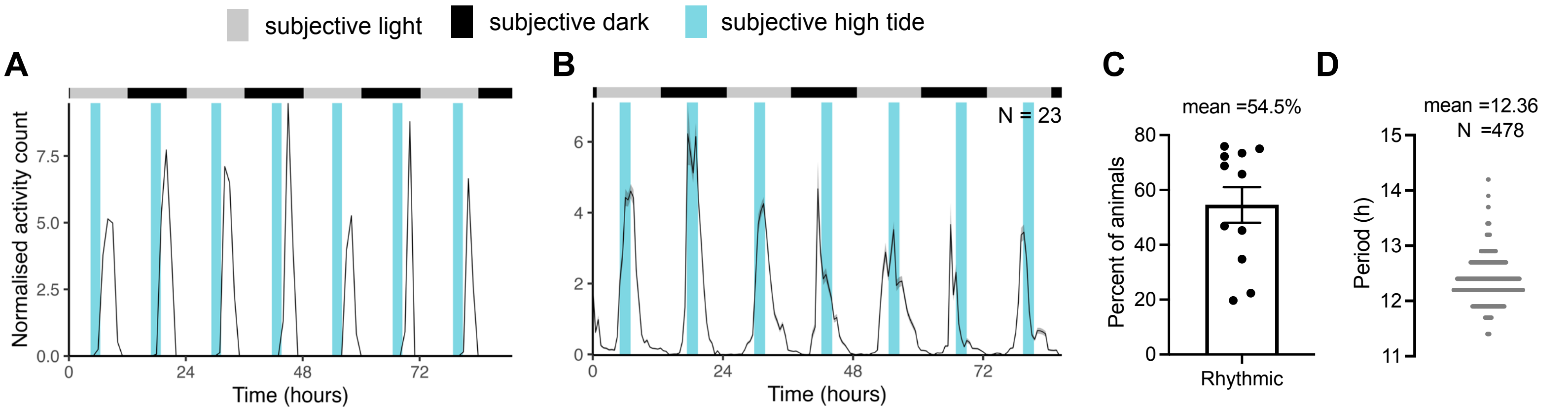

subjective light
subjective dark
subjective high tide
A
Normalised activity count
0.0
2.5
5.0
7.5
0
24
48
72
Time (hours)
B
N = 23
0
2
4
6
Time (hours)
C
mean =54.5%
Percent of animals
0
20
40
60
80
Rhythmic
D
mean =12.36
N =478
Period (h)
11
12
13
14
15

Figure S7. Circatidal swimming behaviour of beach-collected *Eurydice pulchra* recorded in free-running laboratory conditions. Related to Figure 3.

(A) Normalised swimming activity of a representative individual *E. pulchra*. Blue shadings indicate subjective high tides on home beach.
(B) Mean normalised swimming activity profile for a group of animals under DD from the same experiment as the individual in A (N =23).
(C) Percentage of animals showing rhythmic swimming behaviour from 11 independent experiments, representing 11 beach collections from 2022 to 2024. Error bars indicate SEM.
(D) Individual periods of beach-caught *E. pulchra* swimming activity under DD across all experiments. Mean (±SEM) is 12.36 ±0.02 h (N =478).

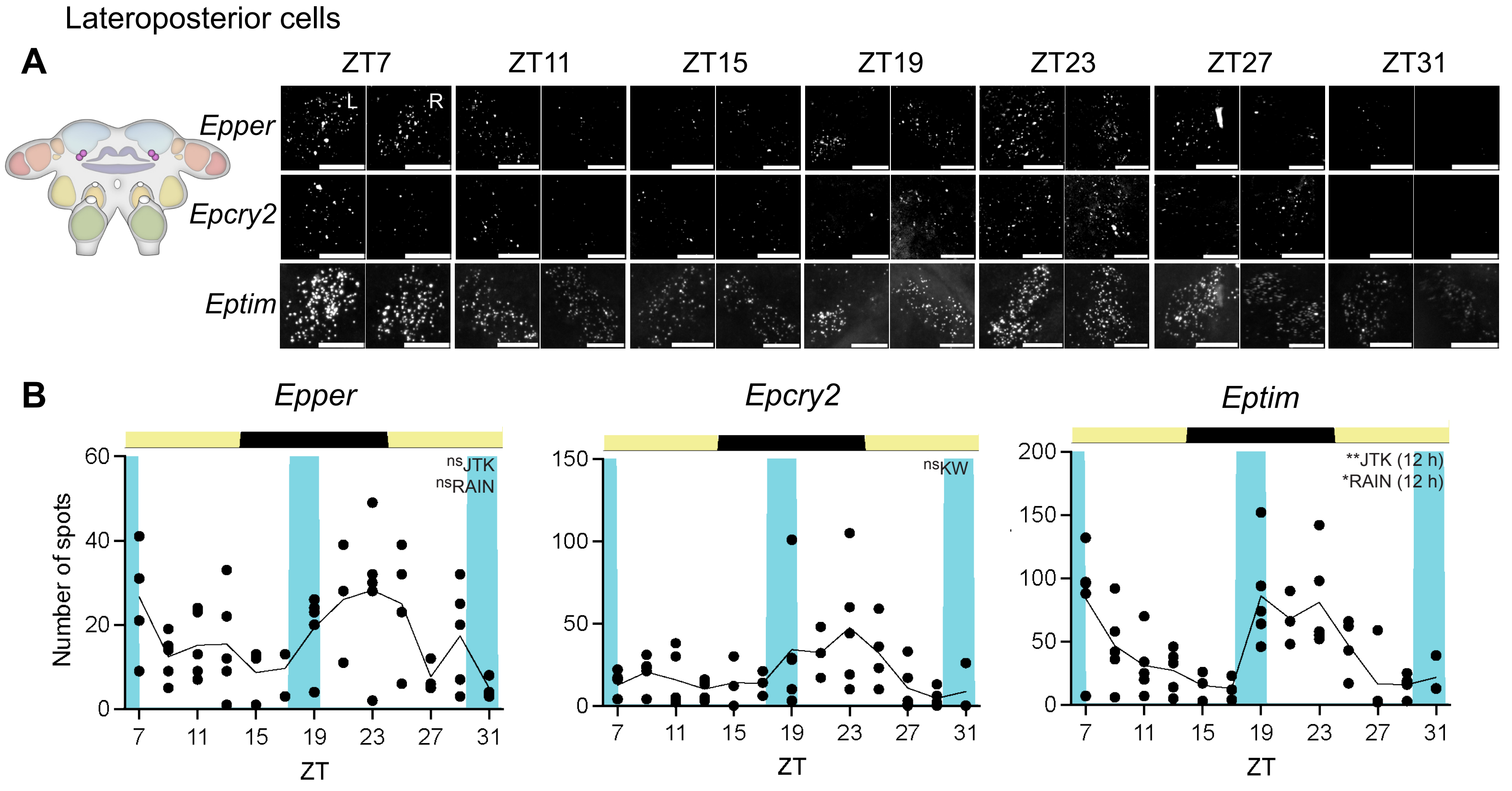

Lateroposterior cells
A
ZT7
ZT11
ZT15
ZT19
ZT23
ZT27
ZT31
L
R
Epper
Epcry2
Eptim
B
Epper
Epcry2
Eptim
Number of spots
nsJTK
nsRAIN
nsKW
**JTK (12 h)
*RAIN (12 h)
ZT

Figure S8. Time course of expression of *per, cry2* and *tim* in lateroposterior cells of tidally rhythmic *E. pulchra* held on a LD cycle. Related to Figure 3.

(A) Left: Cartoon to show location of lateroposterior cell group in the brain and Right: representative maximum intensity Z-projections of *Epper*, *Epcry2* and *Eptim* expression in lateroposterior cells in both hemispheres (L: left, R: right) across ZT (individual HCR-FISH channels in rows).

(B) Plots of mean transcript abundance, quantified as the number of FISH spots, across ZT for *Epper* (left), *Epcry2* (middle) and *Eptim* n (right) in the lateroposterior cells. n =3-5, N =55. Each point is from one brain, total across hemispheres. Inset text indicates statistical tests (JTK-cycle and RAIN) performed to determine 24-h and 12-h rhythmicity with the tested periods in parentheses, following significant time effect by ANOVA or Kruskal-Wallis. p <0.001***, p <0.01**, p ≤0.05*, p >0.05^{ns}. For statistics shown in this figure, refer to Table S2. Scale bars: 10 µm (A).

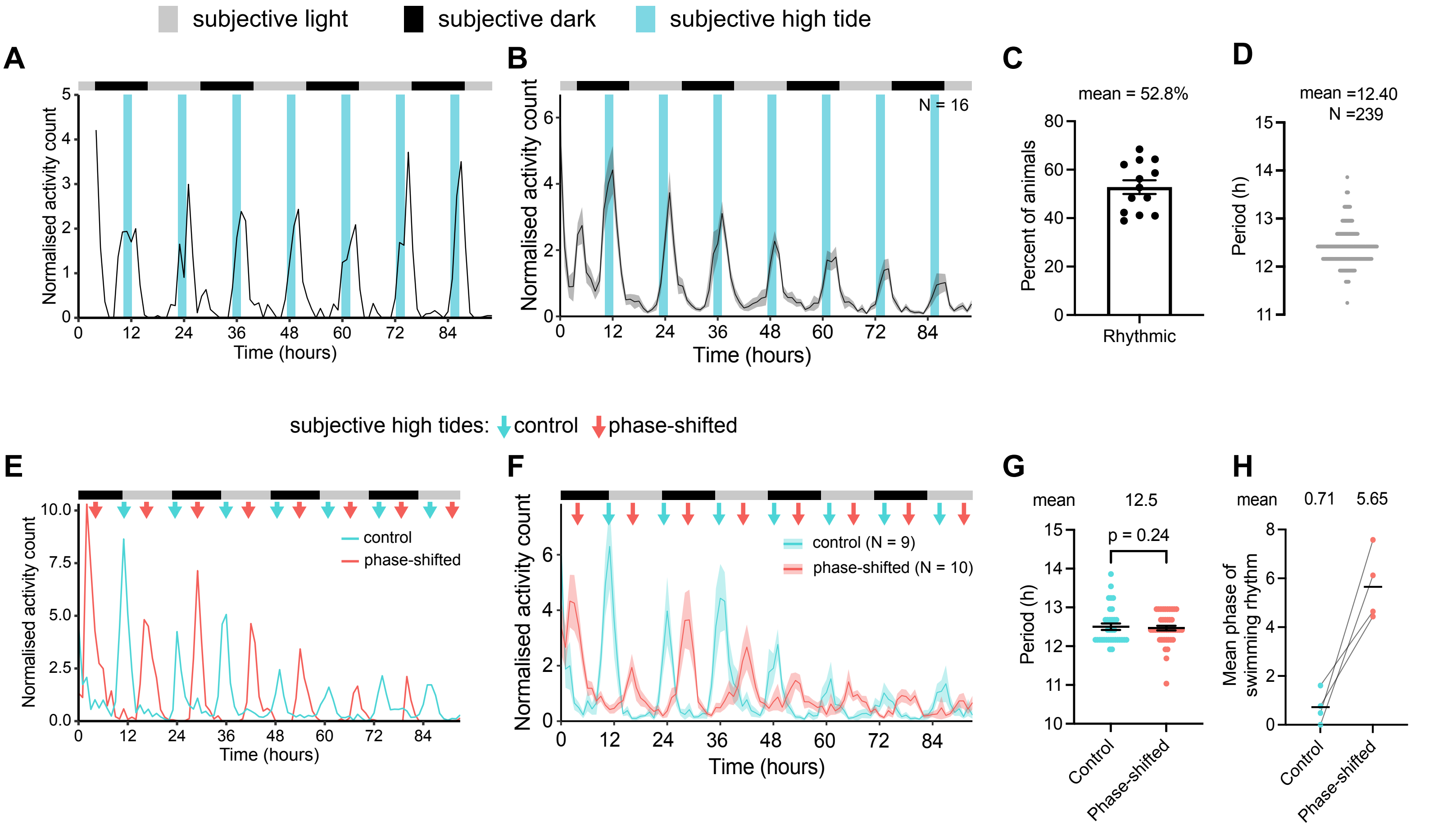

subjective light
subjective dark
subjective high tide
A
B
C
D
Normalised activity count
Time (hours)
N = 16
mean = 52.8%
Percent of animals
Rhythmic
mean =12.40
N =239
Period (h)
subjective high tides: control phase-shifted
E
F
G
H
control
phase-shifted
control (N = 9)
phase-shifted (N = 10)
mean
12.5
p = 0.24
Control
Phase-shifted
0.71
5.65
Mean phase of swimming rhythm

Figure S9. Laboratory-cultured *P. hawaiensis* show circatidal activity rhythms after entrainment to a tidal cycle of 2 hours of mechanical agitation every 12.4 hours simulating high water. Related to Figure 4.

(A) Normalised swimming activity of individual laboratory-cultured *P. hawaiensis* free-running under DD (grey/ black bars), following entrainment to 2 h of agitation every 12.4 h. Blue bars indicate time of subjective tidal agitation.

(B) Mean normalised swimming activity profile for all circatidal rhythmic *P. hawaiensis* under DD (grey/ black bars) from the same experiment as the individual in A (N =16). Blue bars indicate time of subjective tidal agitation, shaded area indicates ±SEM.

(C) Percentage of animals showing a circatidal rhythm in swimming activity after tidal entrainment by agitation. Data from 13 independent experiments (N =456). Error bars indicate SEM.

(D) Individual periods of circatidal locomotor activity under DD of tidally entrained *P. hawaiensis.* Mean (±SEM) is 12.40 ±0.02 h (N =239).

(E) Representative activity recording of two individual laboratory-cultured *P. hawaiensis* free-running under DD (black/ grey bars) following prior tidal entrainment at a period of 12.4 h (cyan, control) or 12.9 h (red, phase-shifted) over 6 days. Arrows indicate times of subjective tidal agitation for the control and phase-shifted animals.

(F) Mean normalised swimming activity profile for all circatidally rhythmic *P. hawaiensis* under DD (grey/ black bars) from the same experiment as the individuals in E ($N_{control}$ =9, $N_{phase\text{-}shifted}$ =10). Arrows indicate times of subjective tidal agitation for the control and phase-shifted groups, shaded area indicates ±SEM.

(G). Individual periods of circatidal activity rhythm of *P. hawaiensis* after tidal entrainment by agitation under control and phase-shifted conditions (4 independent experiments, $N_{control}$ =34, $N_{phase\text{-}shifted}$ =42, Kolmogorov-Smirnov test: D =0.24, p =0.24, n.s.). Horizontal lines indicate mean phase within each group, and error bars indicate SEM.

(H) Mean phase of circatidal activity rhythms of paired control and phase-shifted groups across 4 replicate experiments as in F. The subjective high tide onset of the earliest control group defined phase =0. Horizontal lines indicate mean phase within each group.

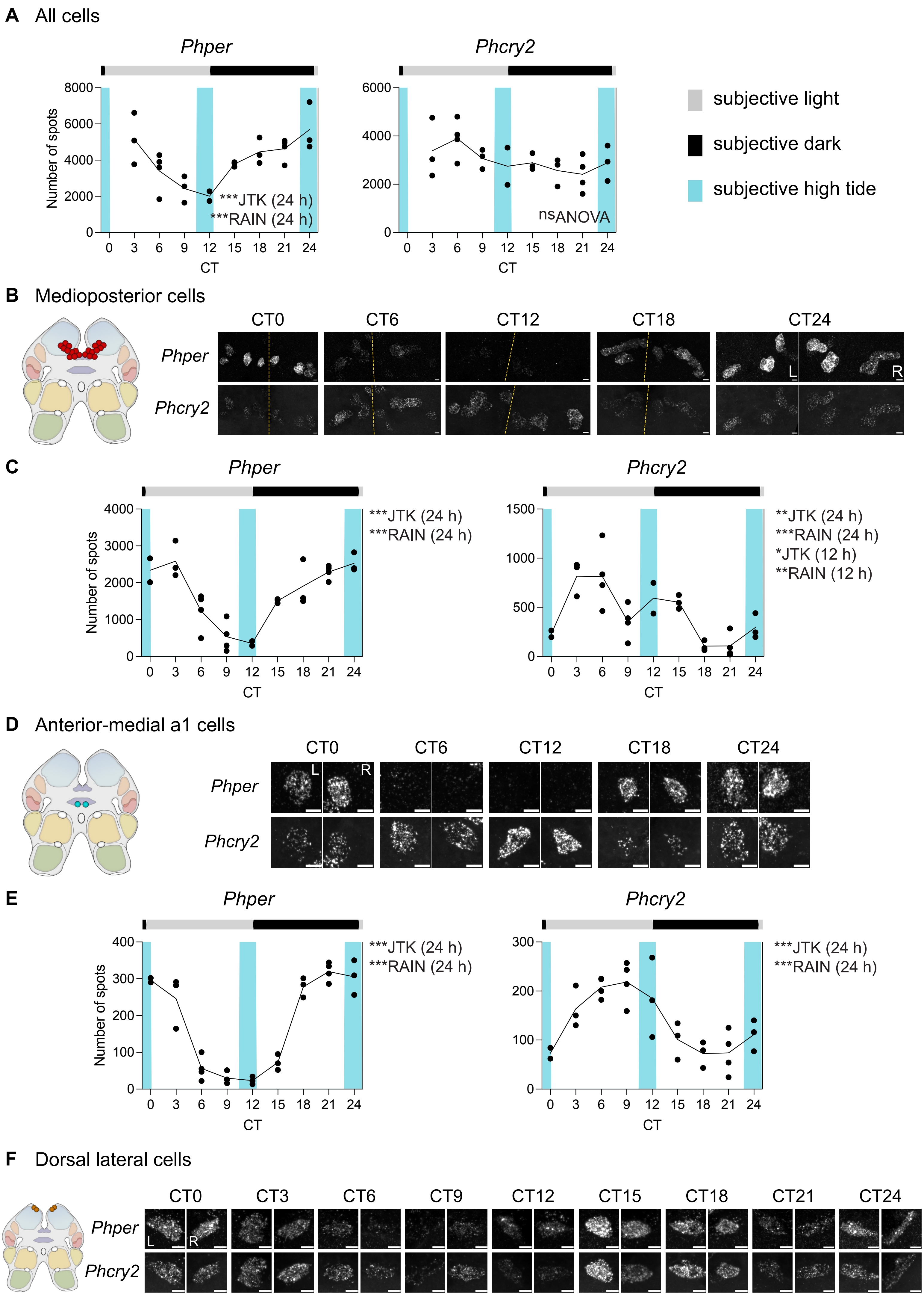

A All cells
Phper
Phcry2
Number of spots
***JTK (24 h)
***RAIN (24 h)
nsANOVA
CT
subjective light
subjective dark
subjective high tide
B Medioposterior cells
CT0
CT6
CT12
CT18
CT24
L
R
C
**JTK (24 h)
***RAIN (24 h)
*JTK (12 h)
**RAIN (12 h)
D Anterior-medial a1 cells
E
F Dorsal lateral cells
CT3
CT9
CT15
CT21

Figure S10. Free-running clock gene expression rhythms in tidally entrained *P. hawaiensis* (tidal phase θ + 1.2 h). Related to Figure 5.

(A) Mean aggregate expression, quantified as the number of FISH spots, of *Phper* and *Phcry2* across all cells plotted across time in tidally entrained animals sampled under DD. n =2-4, N =25.

(B) Left: Cartoon to show location of medioposterior cell group in the brain and Right: representative maximum intensity Z-projections of *Phper* and *Phcry2* expression in the medioposterior cells from both hemispheres (L: left, R: right, yellow dashed lines: midlines) across CT (individual HCR-FISH channels in rows).

(C) Mean transcript abundance, quantified as the number of FISH spots, across CT for *Phper* (left) and *Phcry2* (right)) in the medioposterior cells. n =2-4, N =28.

(D, E) As in B, C for anterior-medial a1 cell group. n =2-4, N =29.

(F) As in B for dorsal-lateral cells exhibiting a tidal pattern of expression (expanded from main Figure 5B).

Each point is from one brain, total across hemispheres. Inset text indicates statistical tests (JTK-cycle and RAIN) performed to determine 24-h and 12-h rhythmicity with the tested periods in parentheses, following significant time effect by ANOVA or Kruskal-Wallis. p <0.001***, p <0.01**, p ≤0.05*, p >0.05^{ns}. KW: Kruskal-Wallis test. For statistics shown in this figure, refer to Table S4. Scale bars: 10 μm (B), 5 μm (D, F).

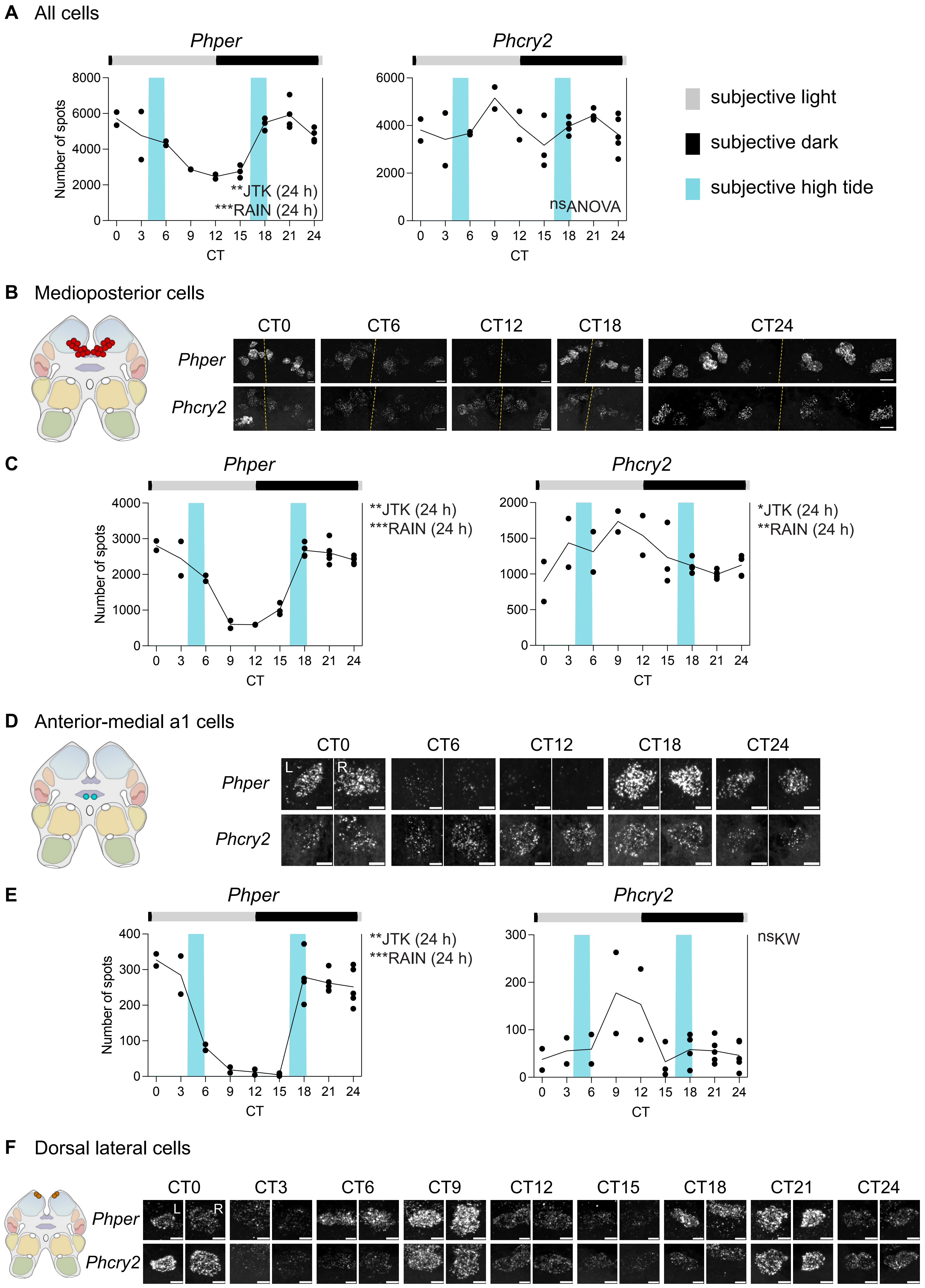
A All cells
Phper
Phcry2
Number of spots
**JTK (24 h)
***RAIN (24 h)
nsANOVA
CT
subjective light
subjective dark
subjective high tide
B Medioposterior cells
CT0
CT6
CT12
CT18
CT24
Phper
Phcry2
C
Phper
Phcry2
**JTK (24 h)
***RAIN (24 h)
*JTK (24 h)
**RAIN (24 h)
Number of spots
CT
D Anterior-medial a1 cells
CT0
CT6
CT12
CT18
CT24
L
R
Phper
Phcry2
E
Phper
Phcry2
**JTK (24 h)
***RAIN (24 h)
nsKW
Number of spots
CT
F Dorsal lateral cells
CT0
CT3
CT6
CT9
CT12
CT15
CT18
CT21
CT24
L
R
Phper
Phcry2

Figure S11. Free-running clock gene expression rhythms in tidally entrained *P. hawaiensis* (tidal phase θ +5.4 h). Related to Figure 5.

(A) Mean aggregate expression, quantified as the number of FISH spots, of *Phper* and *Phcry2* across all cells plotted across time in tidally entrained animals sampled under DD. n =2-5, N =26.

(B) Left: Cartoon to show location of medioposterior cells in the brain and Right: representative maximum intensity Z-projections of *Phper* and *Phcry2* expression in the anterior-medial a1 cells from both hemispheres (L: left, R: right, yellow dashed lines: midlines) across CT (individual HCR-FISH channels in rows).

(C) Mean expression, quantified as the number of FISH spots, across CT for *Phper* (left) and *Phcry2* (right) in the anterior-medial a1 cells. n =2-5, N =27.

(D, E) As in B, C for anterior-medial a1 cell group. n =2-5, N =27.

(F) As in B for dorsal-lateral cells exhibiting a tidal pattern of expression (expanded from main Figure 5D).

Each point is from one brain, total across hemispheres. Inset text indicates statistical tests (JTK-cycle and RAIN) performed to determine 24-h and 12-h rhythmicity with the tested periods in parentheses, following significant time effect by ANOVA or Kruskal-Wallis. p <0.001***, p <0.01**, p ≤0.05*, p >0.05^{ns}. KW: Kruskal-Wallis test. For statistics shown in this figure, refer to Table S5. Scale bars: 10 μm (B), 5 μm (D, F).

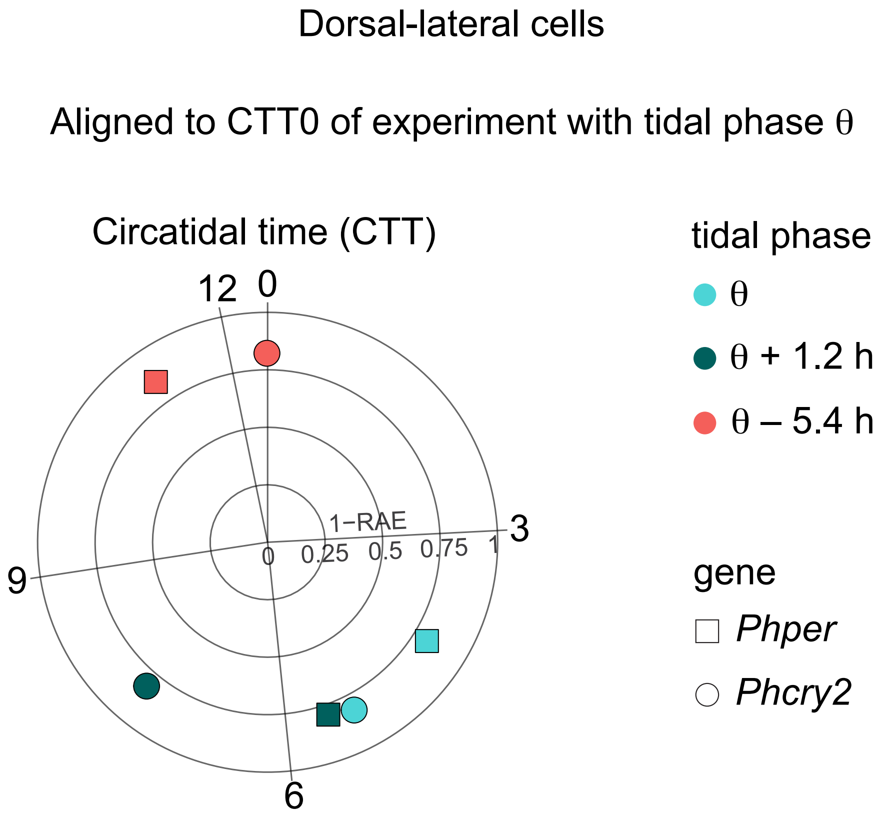

Dorsal-lateral cells
Aligned to CTT0 of experiment with tidal phase θ
Circatidal time (CTT)
12
0
3
6
9
1−RAE
0
0.25
0.5
0.75
1
tidal phase
θ
θ + 1.2 h
θ – 5.4 h
gene
Phper
Phcry2

Figure S12. Acrophase of free-running clock gene expression pattern in the dorsal lateral cells of *P. hawaiensis* entrained to tidal cycles of different phase. Related to Figures 5.

Circular plots of phase of circatidal peak expression of *Phper* and *Phcry2* in the dorsal-lateral cells of *P. hawaiensis* exhibiting circatidal activity rhythms. The phase of each experiment is aligned to circatidal time (CTT) =0 of the experiment with tidal phase θ. Each colour represents an individual experiment at each of different phases (total N =3 X 3). RAE: relative amplitude error.

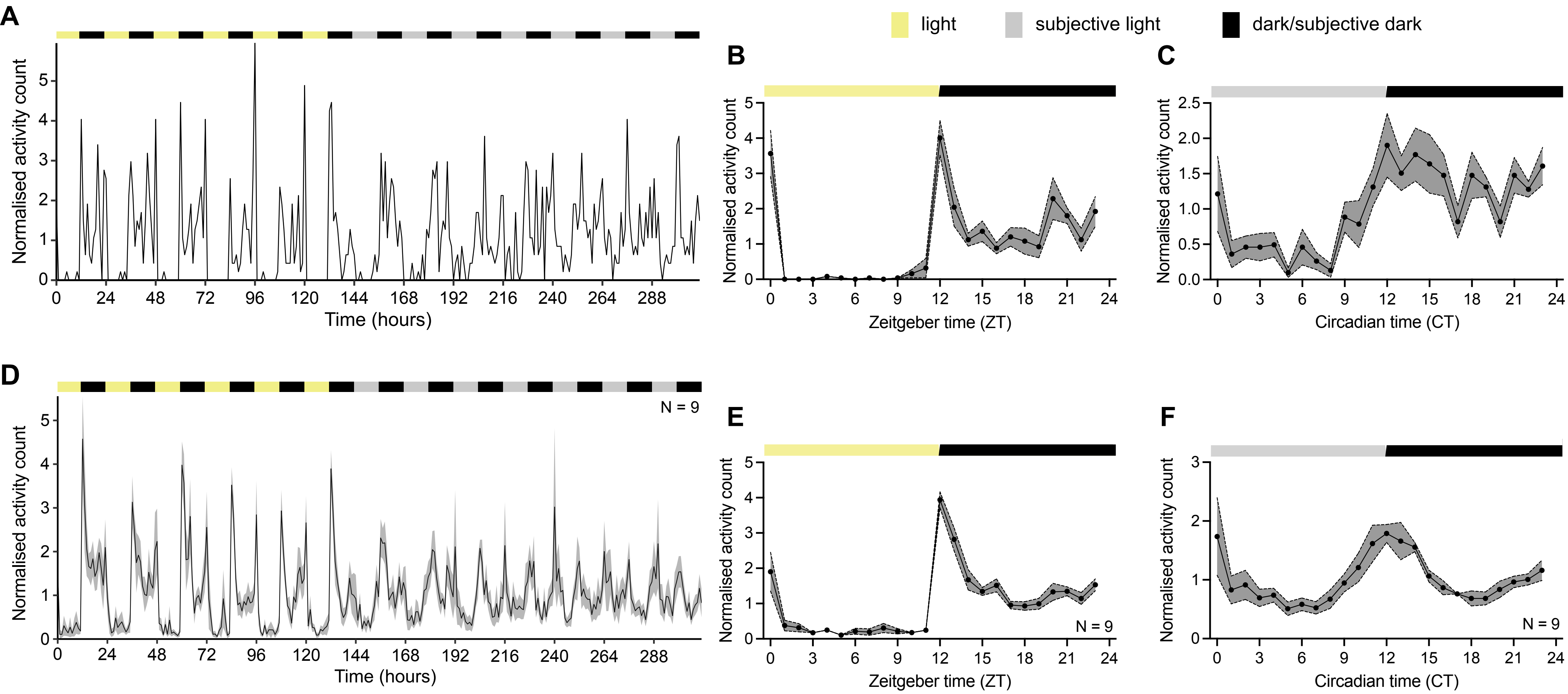
A
B
C
D
E
F
light
subjective light
dark/subjective dark
Normalised activity count
Time (hours)
Zeitgeber time (ZT)
Circadian time (CT)
N = 9

Figure S13. Laboratory-cultured *P. hawaiensis* show circadian activity rhythms after entrainment to LD cycle. Related to Figure 6.

(A) Representative activity recording of individual laboratory-cultured *P. hawaiensis*, which had never been subjected to tidal entrainment, initially maintained under 12 h:12 h L:D cycle (yellow/black bars) and then allowed to free-run under DD (grey/ black bars).

(B) Mean activity profile for the individual from A under LD showing higher activity during night-time. Shaded area indicates ±SEM.

(C) Mean activity profile for the individual from A under DD showing higher activity during subjective night. Shaded area indicates ±SEM.

(D, E and F) as for A, B and C but showing group data as mean ±SEM (shading, N =9).

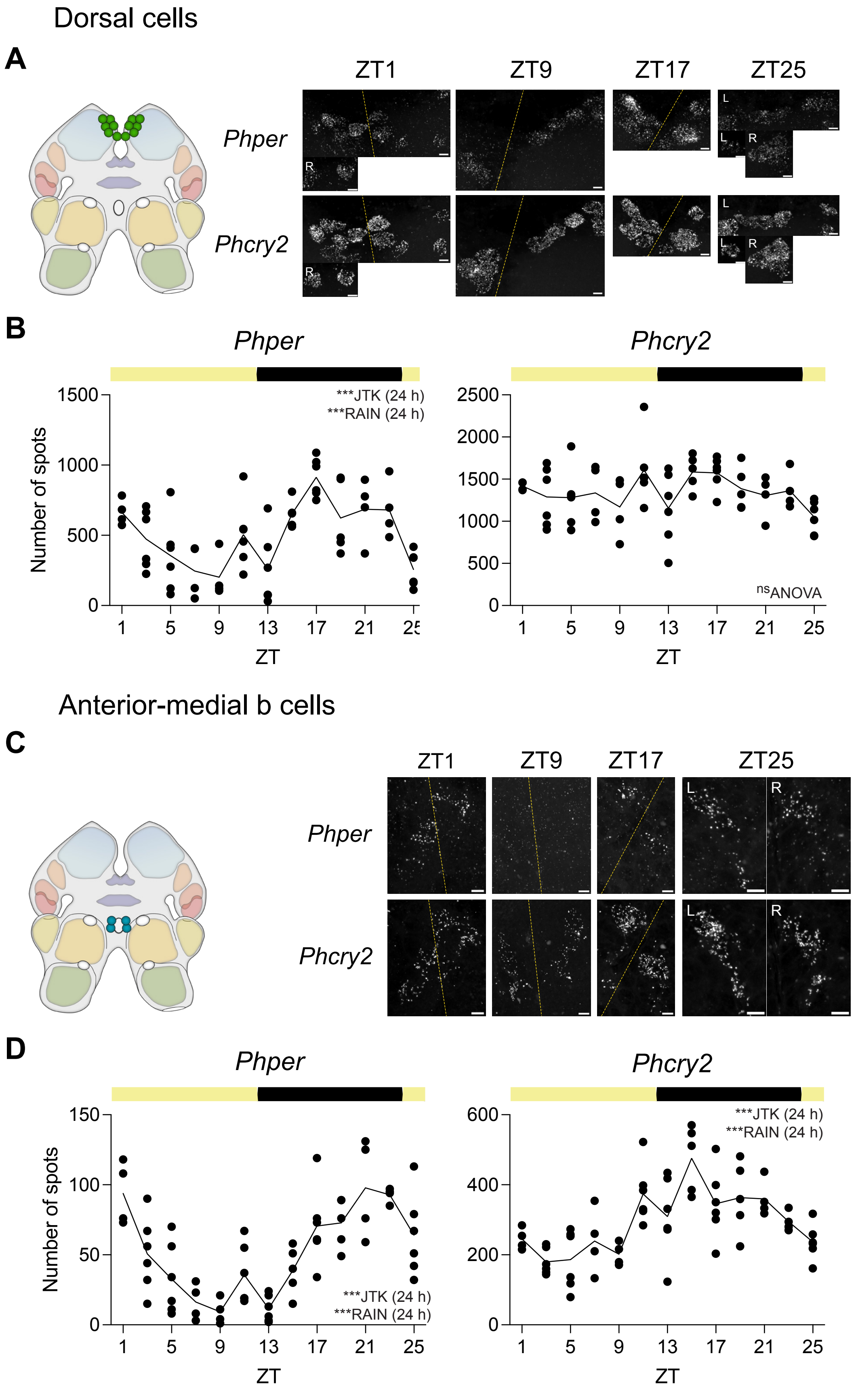

Dorsal cells
A
ZT1
ZT9
ZT17
ZT25
Phper
Phcry2
L
R
B
Phper
Phcry2
***JTK (24 h)
***RAIN (24 h)
nsANOVA
Number of spots
1500
1000
500
0
2500
2000
1
5
9
13
17
21
25
ZT
Anterior-medial b cells
C
ZT1
ZT9
ZT17
ZT25
Phper
Phcry2
L
R
D
Phper
Phcry2
150
100
50
600
400
200
0
***JTK (24 h)
***RAIN (24 h)
Number of spots
ZT

Figure S14. Time course of rhythmic expression of *per* and *cry2* in cells of *P. hawaiensis* synchronised to the LD cycle. Related to Figure 6.

(A) Left: Cartoon to show location of dorsal cell group in the brain and Right: representative maximum intensity Z-projections of *Phper* and *Phcry2* expression in the dorsal cells of both hemispheres (L: left, R: right, yellow dashed lines: midlines) across ZT (individual HCR-FISH channels in rows).

(B) Mean expression, quantified as the number of FISH spots, of *Phper* (left) and *Phcry2* in the dorsal cells plotted across daily time. n =4-6, N =66.

(C, D) as in A, B for anterior-medial b cell group. n =4-6, N =66.

Each point is from one brain, total across hemispheres. Inset text indicates statistical tests (JTK-cycle and RAIN) performed to determine 24-h and 12-h rhythmicity with the tested periods in parentheses, following significant time effect by ANOVA or Kruskal-Wallis. $p < 0.001$***, $p < 0.01$**, $p \leq 0.05$*, $p > 0.05^{ns}$. Scale bars: 5 μm. For statistics shown in this figure, refer to Table S6.

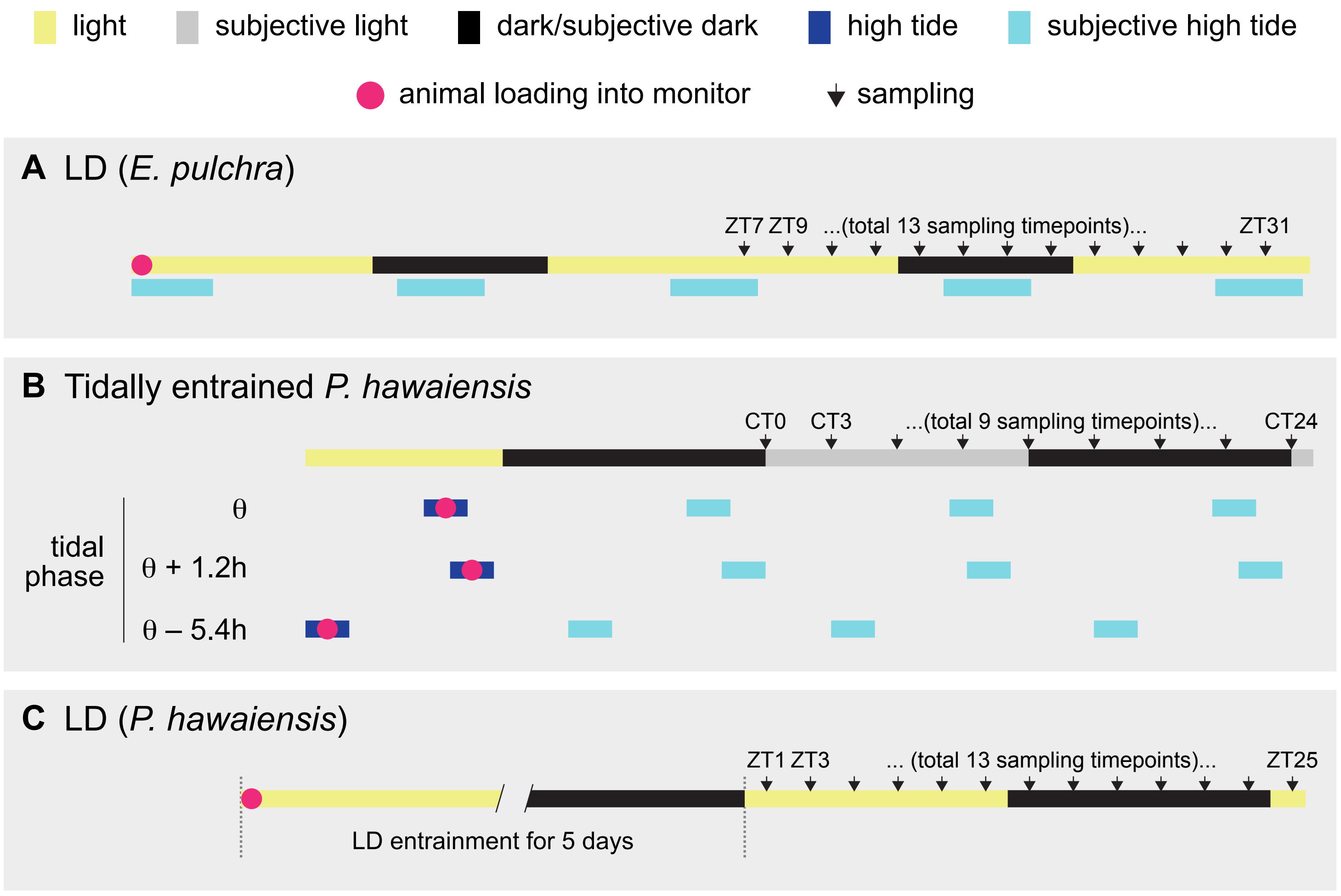
light
subjective light
dark/subjective dark
high tide
subjective high tide
animal loading into monitor
sampling
A LD (E. pulchra)
ZT7 ZT9 ...(total 13 sampling timepoints)... ZT31
B Tidally entrained P. hawaiensis
CT0 CT3 ...(total 9 sampling timepoints)... CT24
tidal phase
θ
θ + 1.2h
θ – 5.4h
C LD (P. hawaiensis)
ZT1 ZT3 ... (total 13 sampling timepoints)... ZT25
LD entrainment for 5 days

Figure S15. HCR-FISH time-course design. Related to Figures 3, 4 , 5 and 6.

(A) Field-collected *E. pulchra* were loaded into activity monitors and maintained under 16 h: 8 h LD cycles. At ZT7 on the day after loading, animals showing circatidal rhythmicity in their swimming activity were sampled every 2 hours, for a total of 13 timepoints.

(B) Laboratory-cultured *P. hawaiensis* which had been subjected to tidal cycles of mechanical agitations were loaded into activity monitors whilst being shaken. At CT0 on the day after loading, animals showing peaks in activity coinciding with the subjective high tide during the night before were sampled every 3 hours, for a total of 9 timepoints.

Time-course design is also described in Table S10.

(C) Laboratory-cultured *P. hawaiensis* that had never been tidally entrained were loaded into activity monitors and maintained under 12 h: 12 h LD cycles for 5 days. Animals showing 24-h rhythmicity over the 5-day LD entrainment period were sampled every 2 hours beginning at ZT1 on day 6, for a total of 13 timepoints.

Time-course design is also described in Table S10.

E. pulchra				*P. hawaiensis*		
Cell group	# of cells	Anatomical location		Cell group	# of cells	Anatomical location
Medioposterior	18	rPBp		Medioposterior	20	rPBp
Medial	10	rPBdp		Medial	6	rPBp
Dorsal	8	rHE/MTp		Dorsal	12	rHE/MTd
Medioanterior	2	rMPa		Dorsal-lateral	4	rHE/MTd
Lateroposterior	4	rMTp		Anterior-lateral	2	rHE/MTa
Dorsoanterior	4	rHE/MTa		Anterior-medial a1	2	rMPa
Lateroanterior	2	rLOva		Anterior-medial a2	2	rMPa
Ventroposterior	8-12	rMPp		Anterior-medial b	4	rMPa
				Anterior-medial c	8-10	rMPa

Table S1. Summary of cell groups enriched for *per* and *cry2* expression in *E. pulchra* and *P. hawaiensis*. Related to Figures 1, 2.

The anatomical location of each cell group was defined as the relative position of their resident cell body rind to anatomical structure of the brain and is abbreviated as follows: r: cell body rind, a: anterior, p: posterior, v: ventral, d: dorsal, HE/MT: hemi-ellipsoid body/ medulla terminalis, PB: protocerebral bridge, MP: medial protocerebrum, LO: lobula.

Groups with daily/ circadian or tidal rhythms across studies. See Tables S2- 6 for statistical details.

Note: because of its location, the ventroposterior cell group in *E. pulchra* could not be imaged in the time-course studies.

Gene	Cell group	Significant variation with time					24-h rhythmicity		12-h rhythmicity	
		Test	df	Test statistic	p-value	Effect size	JTK_cycle	RAIN	JTK_cycle	RAIN
Epper	all clock cells	ANOVA	12	2.590	0.011	0.425	0.003	0.001	1.000	0.852
	medioposterior	ANOVA	12	2.522	0.014	0.431	<0.001	0.001	1.000	0.990
	medial	ANOVA	12	2.617	0.011	0.428	0.022	0.003	1.000	0.711
	large-dorsal	ANOVA	12	2.090	0.041	0.391	0.003	0.192	1.000	1.000
	lateroanterior	KW	12	20.792	0.054	0.187				
	lateroposterior	ANOVA	12	2.200	0.030	0.386	0.459	1.000	1.000	0.999
	dorsoanterior	ANOVA	12	2.346	0.020	0.390	0.602	1.000	0.943	1.000
	dorsal	ANOVA	12	1.718	0.100	0.346				
Epcry2	all clock cells	KW	12	24.662	0.017	0.301	<0.001	<0.001	1.000	0.747
	medioposterior	ANOVA	12	2.563	0.013	0.435	<0.001	<0.001	1.000	0.886
	medial	KW	12	19.532	0.076	0.179				
	large-dorsal	KW	12	26.095	0.010	0.361	<0.001	0.017	1.000	0.996
	lateroanterior	KW	12	18.405	0.104	0.133				
	lateroposterior	KW	12	19.553	0.076	0.176				
	dorsoanterior	KW	12	14.737	0.256	0.061				
	dorsal	KW	12	16.606	0.165	0.118				

Gene	Cell group	Significant variation with time					24-h rhythmicity		12-h rhythmicity	
		Test	df	Test statistic	p-value	Effect size	JTK_cycle	RAIN	JTK_cycle	RAIN
Eptim	all clock cells	KW	12	26.588	0.009	0.347	0.034	0.009	0.034	0.008
	medioposterior	KW	12	20.747	0.054	0.219				
	medial	KW	12	29.999	0.003	0.429	0.170	0.028	0.046	0.005
	large-dorsal	KW	12	17.331	0.138	0.137				
	lateroanterior	KW	12	40.436	<0.001	0.592	0.005	0.001	0.092	0.002
	lateroposterior	KW	12	31.599	0.002	0.456	0.556	1.000	0.009	0.042
	dorsoanterior	KW	12	30.541	0.002	0.412	0.539	0.545	0.169	0.050
	dorsal	KW	12	19.182	0.084	0.184				

Table S2. Statistical tests for all gene-cell group combinations for the HCR-FISH time-course experiment of tidally active, beach-collected *E. pulchra* under LD. Related to Figure 3 and Figure S8. Effect size quantified as eta squared. KW: Kruskal-Wallis test. Cell groups with statistically significant periodicities.

Gene	Cell group	Significant variation with time					24-h rhythmicity		12-h rhythmicity		Cosinor fit	
		Test	df	Test statistic	p-value	Effect size	JTK_cycle	RAIN	JTK_cycle	RAIN	phase	RAE
Phper	all clock cells	ANOVA	8	11.99	<0.001	0.813	<0.001	<0.001	0.299	0.083	22.33	0.16
	mediposterior	ANOVA	8	62.35	<0.001	0.954	<0.001	<0.001	0.381	0.262	22.32	0.07
	anterior-medial a1	ANOVA	8	43.95	<0.001	0.934	<0.001	<0.001	0.388	0.793	22.31	0.06
	anterior-medial b	ANOVA	8	2.253	0.059	0.429						
	anterior-medial c	ANOVA	8	1.856	0.125	0.426						
	dorsal	ANOVA	8	1.962	0.099	0.406						
	anterior-lateral	ANOVA	8	1.090	0.403	0.267						
	anterior-medial a2	ANOVA	8	2.157	0.068	0.408						
	medial-1	ANOVA	8	1.031	0.441	0.256						
	medial-2	ANOVA	8	1.180	0.353	0.291						
	dorsal-lateral	ANOVA	8	6.584	<0.001	0.678	1	0.034	0.006	<0.001	4.20	0.18
Phcry2	all clock cells	ANOVA	8	3.942	0.005	0.589	0.441	0.023	0.087	0.004		
	medioposterior	ANOVA	8	15.13	<0.001	0.835	<0.001	<0.001	0.059	0.002	9.35	0.14
	anterior-medial a1	ANOVA	8	9.814	<0.001	0.758	<0.001	<0.001	0.930	0.567	8.86	0.14
	anterior-medial b	ANOVA	8	1.501	0.209	0.333						
	anterior-medial c	KW	8	7.572	0.476	−0.021						
	dorsal	ANOVA	8	1.391	0.252	0.326						
	anterior-lateral	ANOVA	8	0.7758	0.628	0.205						
	anterior-medial a2	ANOVA	8	0.4122	0.903	0.117						
	medial-1	ANOVA	8	1.387	0.252	0.316						
	medial-2	KW	8	8.827	0.357	0.036						
	dorsal-lateral	ANOVA	8	6.866	<0.001	0.687	1	0.086	0.002	<0.001	5.26	0.18

Table S3. Statistical tests and cosinor analysis (with defined periods of 24-h and/or 12.4-h, whenever appropriate) for all gene-cell group combinations for the HCR-FISH time-course experiment of *P. hawaiensis* entrained to tidal phase θ. Related to Figure 4 and Table S7. Effect size quantified as eta squared. Phase derived from cosinor fit is corrected to Circadian Time (CT) =0 or Circatidal Time (CTT) =0, as appropriate. KW: Kruskal-Wallis test; RAE: relative amplitude error. Cell groups with statistically significant periodicities.

Gene	Cell group	Significant variation with time					24-h rhythmicity		12-h rhythmicity		Cosinor fit	
		Test	df	Test statistic	p-value	Effect size	JTK_cycle	RAIN	JTK_cycle	RAIN	phase	RAE
Phper	all clock cells	ANOVA	7	5.289	0.002	0.685	<0.001	<0.001	1	0.841	23.03	0.18
	medioposterior	ANOVA	8	12.96	<0.001	0.845	<0.001	<0.001	0.772	0.462	22.90	0.11
	anterior-medial a1	ANOVA	8	49.29	<0.001	0.952	<0.001	<0.001	1	0.753	22.17	0.06
	anterior-medial b	KW	8	16.20	0.040	0.304	0.232	0.003	0.772	0.106		
	anterior-medial c	ANOVA	7	1.255	0.335	0.369						
	dorsal	ANOVA	8	0.7518	0.648	0.273						
	anterior-lateral	ANOVA	7	2.212	0.083	0.462						
	anterior-medial a2	ANOVA	8	1.732	0.152	0.410						
	medial-1	ANOVA	8	0.5012	0.84	0.182						
	medial-2	ANOVA	7	0.4214	0.876	0.148						
	dorsal-lateral	ANOVA	8	7.212	<0.001	0.743	0.232	0.003	0.016	<0.001	4.53	0.21
Phcry2	all clock cells	ANOVA	7	1.397	0.269	0.365						
	medioposterior	ANOVA	8	7.967	<0.001	0.770	0.005	<0.001	0.023	0.001	7.01	0.24
	anterior-medial a1	ANOVA	8	6.807	<0.001	0.731	<0.001	<0.001	1	0.773	7.88	0.13
	anterior-medial b	ANOVA	8	2.880	0.026	0.535	0.222	0.014	1	0.460		
	anterior-medial c	ANOVA	7	0.3891	0.894	0.154						
	dorsal	ANOVA	8	0.9256	0.522	0.316						
	anterior-lateral	ANOVA	7	0.6081	0.742	0.191						
	anterior-medial a2	ANOVA	8	1.667	0.168	0.400						
	medial-1	ANOVA	8	0.6999	0.688	0.237						
	medial-2	ANOVA	7	1.691	0.178	0.410						
	dorsal-lateral	ANOVA	8	4.908	0.002	0.663	1	0.215	0.016	<0.001	6.58	0.18

Table S4. Statistical tests and cosinor analysis (with defined periods of 24-h and/ or 12.4-h, whenever appropriate) for all gene-cell group combinations for the HCR-FISH time-course experiment of *Parhyale hawaiensis* entrained to tidal phase θ +1.2 h. Related to

Figure 5, Figure S10 and Table S7. Effect size quantified as eta squared. Phase derived from cosinor fit is corrected to Circadian Time (CT) =0 or Circatidal Time (CTT) =0, as appropriate. KW: Kruskal-Wallis test; RAE: relative amplitude error. Cell groups with statistically significant periodicities.

Gene	Cell group	Significant variation with time					24-h rhythmicity		12-h rhythmicity		Cosinor fit	
		Test	df	Test statistic	p-value	Effect size	JTK_cycle	RAIN	JTK_cycle	RAIN	phase	RAE
Phper	all clock cells	KW	8	19.98	0.010	0.705	0.002	<0.001	0.627	0.25	22.32	0.16
	medioposterior	KW	8	20.39	0.009	0.689	0.002	<0.001	1	0.879	22.57	0.11
	anterior-medial a1	ANOVA	8	23.67	<0.001	0.913	0.005	<0.001	0.829	0.654	22.73	0.12
	anterior-medial b	ANOVA	8	1.912	0.121	0.096						
	anterior-medial c	KW	8	9.893	0.273	0.111						
	dorsal	ANOVA	8	1.808	0.145	0.460						
	anterior-lateral	KW	8	11.99	0.152	0.234						
	anterior-medial a2	KW	8	6.759	0.563	−0.073						
	medial-1	KW	8	11.56	0.172	0.223						
	medial-2	KW	8	7.392	0.495	-0.036						
	dorsal-lateral	ANOVA	8	7.469	<0.001	0.768	1	0.277	<0.001	<0.001	4.40	0.15
Phcry2	all clock cells	ANOVA	8	1.544	0.215	0.421						
	medioposterior	ANOVA	8	2.537	0.048	0.530	0.016	0.001	1	0.84	9.06	0.23
	anterior-medial a1	KW	8	10.06	0.261	0.114						
	anterior-medial b	ANOVA	8	1.129	0.391	0.334						
	anterior-medial c	ANOVA	8	2.131	0.090	0.501						
	medial-1	ANOVA	8	1.497	0.234	0.428						
	medial-2	ANOVA	8	0.8941	0.542	0.296						
	dorsal	KW	8	13.51	0.096	0.324						
	anterior-lateral	ANOVA	8	4.823	0.003	0.694	1	0.203	<0.001	<0.001	3.93	0.17
	anterior-medial a2	KW	8	12.26	0.14	0.251						
	dorsal-lateral	ANOVA	8	8.943	<0.001	0.799	0.758	0.085	<0.001	<0.001	5.59	0.18

Table S5. Statistical tests and cosinor analysis (with defined periods of 24-h and/ or 12.4-h, whenever appropriate) for all gene-cell group combinations for the HCR-FISH time-course experiment of *P. hawaiensis* entrained to tidal phase θ – 5.4 h. Related to Figure 5,

Figure S11 and Table S7. Effect size quantified as eta squared. Phase derived from cosinor fit is corrected to Circadian Time (CT) =0 or Circatidal Time (CTT) =0, as appropriate. KW: Kruskal-Wallis test; RAE: relative amplitude error. Cell groups with statistically significant periodicities.

Gene	Cell group	Significant variation with time					24-h rhythmicity		12-h rhythmicity		Cosinor fit	
		Test	df	Test statistic	p-value	Effect size	JTK_cycle	RAIN	JTK_cycle	RAIN	phase	RAE
Phper	all clock cells	ANOVA	12	14.28	<0.001	0.764	<0.001	<0.001	1	0.250	21.14	0.11
	medioposterior	KW	12	54.45	<0.001	0.801	<0.001	<0.001	1	0.515	21.62	0.07
	anterior-medial a1	KW	12	57.04	<0.001	0.850	<0.001	<0.001	1	0.717	21.05	0.09
	anterior-medial b	ANOVA	12	9.073	<0.001	0.673	<0.001	<0.001	1	0.791	21.55	0.11
	anterior-medial c	ANOVA	12	1.697	0.095	0.285						
	dorsal	ANOVA	12	6.225	<0.001	0.585	<0.001	<0.001	1	0.193	19.11	0.18
	anterior-lateral	KW	12	13.09	0.363	0.021						
	anterior-medial a2	KW	12	20.94	0.051	0.172						
	medial-1	KW	12	24.66	0.017	0.239	1	0.868	1	0.014		
	medial-2	KW	12	20.18	0.064	0.157						
	dorsal-lateral	ANOVA	12	3.973	<0.001	0.474	0.103	0.003	1	0.051		
Phcry2	all clock cells	KW	12	26.16	0.010	0.267	0.05	0.001	1	0.019	11.70	0.36
	medioposterior	ANOVA	12	12.40	<0.001	0.737	<0.001	<0.001	0.014	<0.001	9.03	0.16
	anterior-medial a1	KW	12	41.79	<0.001	0.562	<0.001	<0.001	1	0.219	8.28	0.12
	anterior-medial b	ANOVA	12	6.574	<0.001	0.598	<0.001	<0.001	1	0.580	16.39	0.14
	anterior-medial c	ANOVA	12	2.203	0.026	0.341	0.049	0.009	1	0.034	15.78	0.34
	dorsal	ANOVA	12	1.792	0.074	0.289						
	anterior-lateral	ANOVA	12	0.9371	0.518	0.178						
	anterior-medial a2	KW	12	15.15	0.233	0.061						
	medial-1	ANOVA	12	1.233	0.286	0.218						
	medial-2	ANOVA	12	2.057	0.037	0.322	1	0.951	1	0.989		
	dorsal-lateral	ANOVA	12	1.501	0.153	0.254						

Table S6. Statistical tests and cosinor analysis (with defined periods of 24-h whenever appropriate) for all gene-cell group combinations for the HCR-FISH time-course experiment of *P. hawaiensis* synchronised to and sampled under LD. Related to Figure 6 and Figure

S14. Effect size quantified as eta squared. Phase derived from cosinor fit is corrected to Zeitgeber Time (ZT) =0. KW: Kruskal-Wallis test; RAE: relative amplitude error. Cell groups with statistically significant periodicities.

Gene	Cell group	Mean phase	Mean vector length	Number of experiments
Circadian oscillations				
Phper	All clock cells	22.56	1.00	3
	Medioposterior	22.60	1.00	3
	Anterior-medial a1	22.40	1.00	3
Phcry2	Medioposterior	8.48	0.96	3
	Anterior-medial a1	8.37	0.99	2
Circatidal oscillations				
Phper	Dorsal-lateral	4.38	1.00	3
Phcry2		5.81	0.96	3

Table S7. Circular statistics of gene-cell group combinations that show statistically significant rhythmicity (at 24-h or 12-h) across 3 HCR-FISH time-course experiments of tidally entrained *P. hawaiensis*. Related to Figures 4, 5, 7, Figures S10, 11, Tables S3 -5.

Probe/target genes	NCBI accession #	Probe hairpin	Number of probe pairs	Lot number	Relevant figure(s)
EpBmal1	KC885968.1	B3	20	PRP939	Fig. 1, Fig. S3-4
EpBmal1 additional	KC885968.1	B3	40	RTA402	Fig. 1, Fig. S3-4
EpClk 1-9	KC885973.1	B1	40	RTA400	Fig. 1, Fig. S3
EpClk 1-9 additional	KC885973.1	B1	29	RTE285	Fig. 1, Fig. S3
Epcry2	KC885970.1	B1	20	RTA399	Fig. 1, Fig. S3, 6
Epcry2	KC885970.1	B3	20	RTO453	Fig. 1, Fig. S8
Epper	KC885967.1	B2	20	RTO454	Fig. 1, 3, Fig. S3, 8
Eptim	KC885969.1	B1	20	PRP937	Fig. 1, 3, Fig. S3, 8
PhBmal1	Not applicable. Sequences from Hunt, Mallon & Rosato (2019)	B1	38	RTC080	Fig. 1, Fig. S3-4
Phper		B1/B2	30	RTK407/RTC082	Fig. 1, 4-6, Fig. S3, 10-11, 13
PhClk-fragment 1		B2	40	RTG497	Fig. 1, 4-6 Fig. S3
PhClk-fragment 2		B2	28	RTG498	Fig. 1 Fig. S3
Phcry2		B3	30	RTC083	Fig. 1, 4-6, Fig. S3, 6, 10-11, 13
deGFP	Not applicable. Sequence provided by Molecular Instruments Inc.	B1	12	RTA210	Fig. S3
deGFP		B2	12	PRQ740	Fig. S3

Table S8. The targets, hairpin identities and lot numbers of the HCR-FISH primary probes used to generate the data described in this study. Relate to STAR Methods.

**Probe/target gene**	**NCBI accession #**	**Amplifier**	**ACD cat number**
Epper-C4	KC885967.1	C4	578211-C4
EpClk5-C1	KC885973.1	C1	578151-C1
EpBmal1-C2	KC885968.1	C2	578191-C2
-ve control			

* Negative control probe is designed against the DapB gene of *Bacillus subtilis* strain

Table S9. The names, hairpin identities and lot numbers of the RNAscope technology FISH probes. Related to STAR Methods.

Experiment	Sampling condition	First sampling time-point	*N* total animals sampled	Sampling interval	1st subjective high tide onset during sampling	HCR-FISH primary probes	HCR-FISH hairpins (AF: AlexaFluor)	Relevant figures/tables
14h:10h LD-maintained *E. pulchra* (Figure S15A)								
LD (*E. pulchra*)	LD	ZT7	65	2	ZT18.4	*Eptim*-B1, *Epper*-B2, *Epcry2*-B3	B1-AF647, B2-AF546, B3-AF488	Figure 3, Figure S8, Table S2
Tidally entrained *P. hawaiensis* (Figure S15B)								
Tidal θ *P. hawaiensis*	DD	CT0	37	3	CT9.2	*Phper*-B2, *Phcry2*-B3	B2-AF546, B3-AF488,	Figure 4, Table S3
Tidal θ + 1.2h *P. hawaiensis*	DD	CT0	35	3	CT10.4	*Phper*-B2, *Phcry2*-B3	B2-AF647, B3-AF488,	Figure 5, Figure S10, Table S4
Tidal θ – 5.4h *P. hawaiensis*	DD	CT0	35	3	CT3.8	*Phper*-B2, *Phcry2*-B3	B2-AF647, B3-AF488,	Figure 5, Figure S11, Table S5
12h:12h LD-synchronised *P. hawaiensis* (Figure S15C)								
LD (*P. hawaiensis*)	LD	ZT1	79	2	N.A.	*Phper*-B1, *Phcry2*-B3	B1-AF647, B4-AF647	Figure 6, Figure S13, Table S6

Table S10. FISH time-course designs for investigating clock gene expression rhythms. Related to Figures 3-6, Figures S10, S11, S13, S15 and Tables S2-S6.
