# Supplementary material for: Expression of clock genes tracks daily and tidal time in brains of intertidal crustaceans Eurydice pulchra and Parhyale hawaiensis
Source: Curr Biol. Author manuscript; Available in PMC 2025 Jul 30. (PMC7617966; doi:10.1016/j.cub.2025.04.047)
Supplement: Supplemental information [file EMS207343-supplement-Supplemental_information.pdf]

SUPPLEMENTAL INFORMATION

Supplemental information can be found online at https://doi.org/10.1016/j.cub.2025.04.047.
